# The tRNA discriminator base defines the mutual orthogonality of two distinct pyrrolysyl-tRNA synthetase/tRNA^Pyl^ pairs in the same organism

**DOI:** 10.1093/nar/gkac271

**Published:** 2022-04-25

**Authors:** Haolin Zhang, Xuemei Gong, Qianqian Zhao, Takahito Mukai, Oscar Vargas-Rodriguez, Huiming Zhang, Yuxing Zhang, Paul Wassel, Kazuaki Amikura, Julie Maupin-Furlow, Yan Ren, Xun Xu, Yuri I Wolf, Kira S Makarova, Eugene V Koonin, Yue Shen, Dieter Söll, Xian Fu

**Affiliations:** BGI-Shenzhen, Shenzhen, 518083, China; BGI-Shenzhen, Shenzhen, 518083, China; College of Life Sciences, University of Chinese Academy of Sciences, Beijing, 100049, China; BGI-Shenzhen, Shenzhen, 518083, China; College of Life Sciences, University of Chinese Academy of Sciences, Beijing, 100049, China; Department of Molecular Biophysics and Biochemistry, Yale University, New Haven, CT 06511, USA; Department of Molecular Biophysics and Biochemistry, Yale University, New Haven, CT 06511, USA; BGI-Shenzhen, Shenzhen, 518083, China; College of Life Sciences, University of Chinese Academy of Sciences, Beijing, 100049, China; BGI-Shenzhen, Shenzhen, 518083, China; Sino-Danish College, University of the Chinese Academy of Sciences, Beijing, China; Department of Microbiology and Cell Science, Institute of Food and Agricultural Sciences, University of Florida, Gainesville, FL 32611, USA; Department of Molecular Biophysics and Biochemistry, Yale University, New Haven, CT 06511, USA; Department of Microbiology and Cell Science, Institute of Food and Agricultural Sciences, University of Florida, Gainesville, FL 32611, USA; Genetics Institute, University of Florida, Gainesville, FL 32611, USA; BGI-Shenzhen, Shenzhen, 518083, China; BGI-Shenzhen, Shenzhen, 518083, China; National Center for Biotechnology Information, National Library of Medicine, National Institutes of Health, Bethesda, MD 20894, USA; National Center for Biotechnology Information, National Library of Medicine, National Institutes of Health, Bethesda, MD 20894, USA; National Center for Biotechnology Information, National Library of Medicine, National Institutes of Health, Bethesda, MD 20894, USA; BGI-Shenzhen, Shenzhen, 518083, China; Guangdong Provincial Key Laboratory of Genome Read and Write, Shenzhen 518120, China; Shenzhen Institute of Synthetic Biology, Shenzhen Institutes of Advanced Technology, Chinese Academy of Sciences, Shenzhen 518055, China; Department of Molecular Biophysics and Biochemistry, Yale University, New Haven, CT 06511, USA; Department of Chemistry, Yale University, New Haven, CT 06511, USA; BGI-Shenzhen, Shenzhen, 518083, China; Guangdong Provincial Key Laboratory of Genome Read and Write, Shenzhen 518120, China

## Abstract

Site-specific incorporation of distinct non-canonical amino acids into proteins via genetic code expansion requires mutually orthogonal aminoacyl-tRNA synthetase/tRNA pairs. Pyrrolysyl-tRNA synthetase (PylRS)/tRNA^Pyl^ pairs are ideal for genetic code expansion and have been extensively engineered for developing mutually orthogonal pairs. Here, we identify two novel wild-type PylRS/tRNA^Pyl^ pairs simultaneously present in the deep-rooted extremely halophilic euryarchaeal methanogen *Candidatus* Methanohalarchaeum thermophilum HMET1, and show that both pairs are functional in the model halophilic archaeon *Haloferax volcanii*. These pairs consist of two different PylRS enzymes and two distinct tRNAs with dissimilar discriminator bases. Surprisingly, these two PylRS/tRNA^Pyl^ pairs display mutual orthogonality enabled by two unique features, the A73 discriminator base of tRNA^Pyl^2 and a shorter motif 2 loop in PylRS2. *In vivo* translation experiments show that tRNA^Pyl^2 charging by PylRS2 is defined by the enzyme's shortened motif 2 loop. Finally, we demonstrate that the two HMET1 PylRS/tRNA^Pyl^ pairs can simultaneously decode UAG and UAA codons for incorporation of two distinct noncanonical amino acids into protein. This example of a single base change in a tRNA leading to additional coding capacity suggests that the growth of the genetic code is not yet limited by the number of identity elements fitting into the tRNA structure.

## INTRODUCTION

Accurate translation of the genetic code depends on several molecular determinants in a tRNA species (identity elements) that are responsible for the recognition by the cognate aminoacyl-tRNA synthetase (aaRS) leading to correct aminoacyl-tRNA (aa-tRNA) formation (1). One important tRNA identity element, the 4^th^ nucleotide from the 3′-end was recognized early on (2) and named the discriminator base; in combination with several other nucleotides/identity elements (mainly in the anticodon and the acceptor helix), it is involved in providing acylation accuracy for at least eighteen aaRSs (1).

Pyrrolysine (Pyl) is the 22^nd^ genetically encoded amino acid found in many archaea and certain bacteria (3). Pyrrolysyl-tRNA synthetase (PylRS) together with tRNA^Pyl^ supply Pyl-tRNA^Pyl^ for incorporation of Pyl into proteins in response to the UAG codon (4). Because many PylRS variants possess remarkably relaxed amino acid specificity (5), and the enzyme does not recognize the tRNA anticodon as identity element (6), PylRS became the tRNA synthetase of choice for synthetic biologists to expand the genetic code with noncanonical amino acids (5,7). Most PylRS enzymes consist of two functional domains, an N-terminal tRNA-binding domain and a C-terminal catalytic domain (CTD) (8,9). The two domains are either contained in the same polypeptide or occur as separate proteins. Recently, the ΔPylSn class of PylRS enzymes was discovered in certain archaea (10–12). The ΔPylSn lack the N-terminal domain and display strong activity of the catalytic CTD alone (13,14). In the few years since their discovery, ΔPylSn enzymes have become a popular tool for incorporation of noncanonical amino acids (ncAAs) in a variety of organisms (13–21).

*Candidatus* Methanohalarchaeum thermophilum HMET1 is a member of the extreme halophilic methanogens (22); they belong to the class *Methanonatronarchaeia* in which the type species is *Methanonatronarchaeum thermophilum* (22,23). The phylogenetic affinity of this group remains a matter of debate (24–26), but recent phylogenetic analysis suggests that it forms the deepest clade in the *Halobacteriota* superphylum that includes classes *Halobacteria*, *Methanosarcinia*, *Methanomicrobia*, *Archaeoglobi*, and *Methanonatronarchaeia* (27). Regardless of the exact position of this clade, it is becoming increasingly clear that methanogenesis originated early in archaeal evolution, likely in the last common ancestor of archaea, and was later lost independently in extant non-methanogen lineages within TACK superphylum and possibly later within *Halobacteriota* superphylum (25,27,28). Pyrrolysine biosynthesis and incorporation of Pyl into several proteins specific to methanogenesis (e.g. methylamine methyltransferases) is observed in the methanogenic lineages of *Halobacteriota* and *Thermoplasmatota* (super)phyla. Thus, it is currently inferred that the Pyl system originated in the common ancestor of these two phyla which are sister groups in the archaeal phylogeny (29). Most genomes encode a single PylRS gene. One of the few exceptions is the HMET1 genome, which harbors two different PylRS genes.

Here we characterize the unusual properties and demonstrate the mutual orthogonality of the two distinct Pyl incorporation systems in *Candidatus* Methanohalarchaeum thermophilum HMET1.

## MATERIALS AND METHODS

### Plasmids

All plasmids used in this study are listed in Supplementary Table S1. Cassettes encoding PylRS, tRNA^Pyl^ and Flag-SAMP1 or Flag-SAMP1(SAMP1_UAG24_) genes were synthesized and cloned into pJAM202c-based vectors by Gibson assembly. To construct plasmids containing noncognate PylRS/tRNA^Pyl^ pairs, PylRS and tRNA^Pyl^ coding sequences were swapped between different vectors by inserting the noncognate tRNA^Pyl^ gene into the vector devoid of tRNA^Pyl^ via Gibson assembly. Cassette amplification, removal of PylRS or tRNA^Pyl^ gene from expression vectors, and site-directed mutagenesis were done by PCR with primers listed in Supplementary Table S2.

### Strains and culture conditions

Strains used in this study are listed in Supplementary Table S1. *Escherichia coli* (*E. coli*) DH5α was used for routine selection and propagation of plasmid DNA. *E. coli* GM2163 was used to prepare plasmid DNA that are free of Dam and Dcm methylations prior to *H. volcanii* transformation. *E. coli* strains were grown in Luria-Bertani (LB) medium at 37°C and *H. volcanii* cells were grown at 42°C in Hv-YPC by rotary shaking at 200 rpm. Media were supplemented with ampicillin (Amp, 0.1 mg/ml), spectinomycin (Sm, 50 μg/ml), tetracycline (Tet, 12.5 μg/ml), kanamycin (Km, 50 mg/ml), novobiocin (Nv, 0.5 μg/ml), Nϵ-Boc-l-lysine (BocK, 1mM) and 3-iodo-l-Phe (3-I-Phe, 1mM) as needed. Solid medium containing 2.0% (wt/vol) agar was used to culture *H. volcanii* cells. *H. volcanii* colonies were streaked from the -80°C freezer stocks onto Hv-YPC agar plates and freshly isolated colonies of *H. volcanii* were inoculated into 2.5 ml medium and grown to log phase (optical density at 600 nm [OD_600_], 0.6 to 1.2). The log-phase cells were then subcultured into 4.0 ml fresh Hv-YPC medium for future assays.

### *In vivo* activity and solubility assays for PylRS/tRNA^Pyl^ pairs in *E. coli*

To measure the amber suppression activity of PylRS/tRNA^Pyl^ pairs in *E. coli*, pSCW11-PylRS/tRNA^Pyl^ and pBAD-sfGFP(150TAG) plasmids were co-transformed into *E. coli* TOP10 cells via electroporation. The transformed cells were recovered for 1 hour in 500 μl SOC medium at 37°C. Then, 100 μl of the transformed cells was inoculated into 1 mL fresh medium (LB containing 50 μg/ml spectinomycin, 50 μg/ml ampicillin and 0.2% arabinose) in 15 mL culture tube, supplemented with or without 1 mM BocK. OD_600_ and sfGFP fluorescence (λex: 480 nm; λem: 520 nm) were measured during overnight culture at 37°C. For PylRS solubility assay, the *E. coli* BL21(DE3) cells containing different pET28a-PylRS plasmids were incubated at 37°C, grown to OD_600_ at 0.6, followed by protein expression induction by addition of 1 mM IPTG at 20 °C for 16 hr. *E. coli* cells were harvested via centrifugation (5,000 *g* for 10 min at 25 °C) and divided into two portions. One portion was resuspended in 2X reducing SDS loading buffer and boiled for 15 minutes prior to SDS-PAGE analysis, which represents the whole cell lysate portion. To prepare the supernatant extract, the harvested cells of the second portion were lysed by French press homogenization (12,000 lb/in^2^) in lysis buffer A (20 mM Tris-HCl [pH 7.4], 1 mM DTT and 300 mM or 500 mM) supplemented with 1 mg·ml^−1^ DNase I (Tiangen) and 1X protease inhibitor cocktail (Roche). The cell lysate was clarified by centrifugation (20,000 *g* for 30 min at 4 °C) to remove the cell debris. The supernatant faction (20 μl) was collected and mixed with an equal volume of 2X SDS-PAGE loading buffer prior to SDS-PAGE analysis. The amount of soluble PylRS proteins by two different treatments was determined on 12% reducing SDS-PAGE gel and stained with Coomassie G250 dye.

### *In vivo* activity assay for the HMET1 PylRS/tRNA^Pyl^ pairs in *H. volcanii*

To examine the amber suppression activity, *H. volcanii* cells expressing different plasmids containing reporter proteins and different combinations of the wild-type or engineered PylRS/tRNA^Pyl^ (listed in Supplementary Table S1) were re-patched on the Hv-YPC plate, then were grown to log phase (OD_600_ at 0.4 to 0.8) in Hv-YPC medium. The mid-log phase cells (OD_600_, 0.6 to 0.8) were inoculated into 4 ml fresh Hv-YPC medium supplemented Nv, with or without 1mM ncAAs (BocK and 3-I-Phe) and grown to stationary phase (OD_600_, 2.0 to 3.0). Cell pellets were harvested by centrifugation (10,000 *g*, 5 min, 25°C) and used to analyze the expression level of SAMP1 reporter proteins by immunoblotting as described below.

### SDS-PAGE and immunoblotting analysis

Cell pellets (2 OD_600_ units) were resuspended in 100 μl of 2X SDS-PAGE loading buffer by vigorous pipetting and vortexing and were then boiled for 15 minutes prior to separation by SDS-PAGE analysis. The OD_600_ of the cell culture was measured to normalize the loading amount of total cellular proteins (0.1 units per lane) and equivalent levels of protein loading were confirmed by Coomassie G250 staining of parallel gels. Total cellular proteins were separated on 12% reducing SDS-PAGE gel followed by electroblotting onto polyvinylidene difluoride (PVDF) membranes (Amersham) according to the BioRad standard protocol. Flag-tagged proteins were detected by alkaline phosphatase-linked anti-Flag M2 monoclonal antibody (Sigma-Aldrich). Immunoreactive antigens were detected by chemiluminescence using horseradish peroxidase (HRP) substrate (Immobilon).

### Immunoprecipitation

*H. volcanii* cells were harvested by centrifugation (8,000 *g*, 10 min, 25°C) and washed twice with TBS buffer (150 mM NaCl, 50 mM Tris-HCl, pH 7.4). Cell pellets were stored at -20°C until further use. The harvested cells were lysed by French press homogenization (12,000 lb/in^2^) in lysis buffer composed of TBS supplemented with 0.1 mM CaCl_2_, 2 mM MgCl_2_, DNase I and protease inhibitor cocktail. Cell debris was removed by centrifugation (20,000 *g*, 40 min, 4°C) and filtration (0.22 μm) and the clarified cell lysate was then applied to an anti-Flag column pre-equilibrated with TBS. The column was 1 cm in diameter and was filled with 0.2–0.4 ml anti-Flag G1 affinity resin (Genscript). The supernatant was incubated with resin at room temperature for 1 h. The bound proteins were washed in 15 mL TBS prior to elution in 100 μl 2X reducing SDS-PAGE loading buffer. The purified Flag-tagged proteins were separated on 12% reducing SDS-PAGE gel and stained with Coomassie G250 dye. The protein band representing SAMP1(SAMP1_UAG24_)-MoaE conjugate at 50 kDa was cut for further analysis with liquid chromatography-tandem mass spectrometry (LC-MS/MS).

### In-gel digestion and LC-MS/MS analysis

Proteins of interest were first excised from the gel. After destaining, reduction and alkylation, the proteins in gel were digested by 0.02 μg/μl trypsin. The peptides were then extracted by acetonitrile. After drying, the peptides were dissolved in 2% (vol/vol) acetonitrile containing 0.1% (vol/vol) formic acid. A total of 1 μg peptides was injected into an Q Exactive HF hybrid quadrupole-Orbitrap mass spectrometer equipped with an UltiMate 3000 UHPLC (Thermo Scientific). The LC conditions and MS parameters were described previously (30). The raw data was analyzed by Proteome Discoverer 1.4 with the Mascot search algorithm. Mascot was set up to search the database including all cellular proteins of *H. volcanii* DS2, common pollution proteins, SAMP1-MoaE protein with BocK and 20 natural amino acids at G24 residue and SAMP1-MoaE protein with BocK, 3-I-Phe and 20 natural amino acids at G14 and G24 residue assuming the digestion with trypsin. Precursor and fragment mass tolerance was set at 10 ppm and 50 mmu, respectively. Carbamidomethyl of cysteine was specified as a fixed modification. Deamidation of Asn and Gln and oxidation of Met were specified as variable modifications. The false-discovery rate (FDR) was specified at 1.0% to filter the peptides. Finally, the MS/MS spectrums were labeled by pLabel software.

### 3D homology structural modeling and analysis

HMET1 PylRS1 and PylRS2 were comparatively modelled by Phyre2 (Protein Homology/analogY Recognition Engine (31) and 100% of residues modelled at > 90% confidence. Visualization and electrostatic potentials analysis of HMET1 PylRS1 and PylRS2 and *M. mazei* PylRS catalytic domain (PDB 2ZIM) were conducted by UCSF Chimera (32). Superposition of the model structure of HMET1 PylRS1/2 onto *Desulfitobacterium hafniense (D. hafniense)* PylRS-tRNA^Pyl^ complex (PDB 2ZNI) and superposition of crystal structures of *H. volcanii* SAMP1 and UbaA onto *E. coli* MoeB-MoaD complex (PDB 3PO0) were also obtained using UCSF Chimera.

### Sequence and phylogenetic analysis

Amino acid sequences and comparative genomic data were retrieved from the archaeal clusters of orthologous genes (arCOG) database (33). Multiple alignment of PylS proteins was constructed using MUSCLE (34). The maximum-likelihood phylogenetic tree was built using IQ-TREE version 2.1.2 based on the best-fitting model, LG + I + G4 (35). PSI-BLAST (36) with default parameters was used to search for PylS homologs in the NCBI non-redundant (NR) database. To identify genes with in-frame stop codons, arCOG profiles were mapped to six-frame genome translations using PSI-BLAST (36),and then, the genome fragments corresponding to the full-length hit matches were scanned for stop codons (excluding the first and the last 20 codons).

## RESULTS

### Occurrence of the ΔPylSn-type pyrrolysyl-tRNA synthetase among archaea

**Figure 1.**
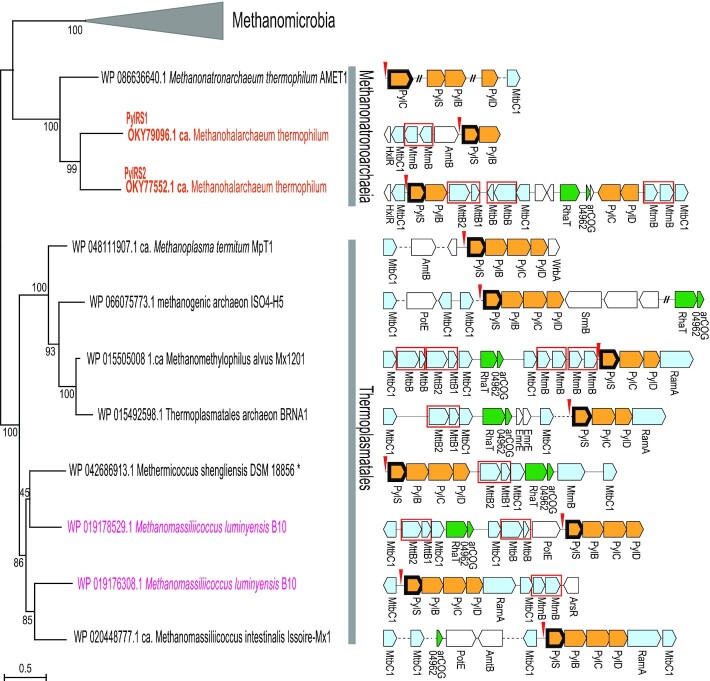
Phylogenetic tree of PylRS proteins and the gene neighborhoods of *pylS* genes. Maximum-likelihood, unrooted phylogenetic tree was built using IQ-tree based on the multiple alignment of PylRS catalytic domain (348 amino-acid sites) with aBayes support values shown for internal branches. The root was placed between *Halobacteriota* and *Thermoplasmatota* phyla according to the established archaeal phylogeny (27). The tree shown in this figure includes only ΔPylSn sequences; the complete tree is provided in the Additional File 1 in Supplementary Information. The leaves are identified by GenBank protein accession numbers and species names. Two species encoding two ΔPylSn proteins are highlighted in orange and magenta for *Candidatus* Methanohalarchaeum thermophilum HMET1 and *Methanomassiliicoccus luminyensis* B10, respectively. Asterisk indicates the sequence from *Methermicoccus shengliensis*, which does not belong to the *Thermoplasmatales* lineage. Genomic neighborhood of *pylS* genes is mapped to each leaf and is shown to the right of the tree. The genes are shown roughly to scale as arrows. If the intergenic distance was shortened for compactness, it is shown by a dashed line or, if genes are not next to each other, using the ‘//’ symbol as a separator. Protein names are provided below the arrows if available. The genes are colored according to their general functions as follows: yellow, genes involved in Pyl biosynthesis; pale blue, genes involved in methanogenesis; green, tightly linked pair of genes coding for putative amino acid transporter RhaT and uncharacterized protein of arCOG04962; white, all other genes. The genes containing in-frame stop codon are marked by red rectangles. Detailed information on these neighborhoods is provided in Supplementary Table S4.

Examination of the archaeal clusters of orthologous genes (arCOGs) (33) revealed that PylRS is encoded in 44 of the 524 archaeal genomes, of which the ΔPylSn enzyme is encoded only in nine complete or nearly complete genomes (Supplementary Table S3). Among the latter, *Methanomassiliicoccus luminyensis* B10 (*Thermoplasmatales*) and *Candidatus* Methanohalarchaeum thermophilum HMET1 (*Methanonatronarchaeia*) encompass two paralogous versions of *ΔPylSn* genes (Figure 1 and Supplementary Table S3). These are the only known organisms that encode two distinct PylRS enzymes (22). Using PylRS from HMET1 (OKY77552.1) as a query, we additionally searched the NCBI non-redundant (NR) database (December 2021) to identify other such genomes, but no sufficiently complete genomes with two or more paralogs of ΔPylSn were found. Therefore, for further analysis, we proceeded with complete genomes from the arCOG database (Supplementary Table S3). Using the multiple amino acid sequence alignment of the catalytic domains of non-redundant PylRS sequences, we reconstructed a phylogenetic tree and mapped the Pyl biosynthesis and incorporation genes *pylS/pylD/pylC* gene neighborhoods to the branches of this tree (Figure 1, Supplementary Table S4 and Additional File 1). Generally, the tree topology was compatible with the phylogeny of the respective archaeal species, with monophyletic *Methanosarcinales*, *Methanonatronarchaeia* and *Thermoplasmales*. The single exception was *pylS* from *Methermicoccus shengliensis* DSM 18856, which belongs to the phylum *Methanosarcinales* and appears to represent a rare case of horizontal gene transfer from a phylogenetically distant archaeal lineage and displacement of Pyl biosynthesis genes. These observations suggest that duplications of *pylS* genes and tRNA^Pyl^ genes (*pylT*) encoded upstream of *pylS* occurred independently in HMET1 and *M. luminyensis* B10.

Further analysis of HMET1 and *M. luminyensis* B10 genomes led to the identification of different sets of genes that appear to be duplicated in addition to *pylS*. In HMET1, the duplicated genes include the Pyl biosynthesis *pylB* gene encoding (2R,3R)-3-methylornithine synthase, *mtmB* encoding monomethylamine methyltransferase and *mtbC1* encoding the methylamine corrinoid protein, whereas in *M. luminyensis* B10, three genes for Pyl biosynthesis (*pylB*, *pylC*, *pylD*), *mtmB* and *mtbC1* are duplicated. Considering that these genes often appear in the vicinity of *pylS*, most likely, in both cases, the duplication involved a large DNA region, with several genes lost or transposed to other locations during subsequent evolution. Next, we analyzed the mono-, bi- and trimethylamine methyltransferase genes (*mtmB*, *mtbB* and *mttB*) containing an in-frame stop codon in the nine genomes encoding ΔPylSn; the number of TAG-containing genes in these genomes ranged from two to six (Supplementary Figure S1). Although the available data are insufficient for rigorous statistical testing, these observations suggest that *pylS* duplication is not tightly linked to duplication(s) of the Pyl encoding genes. Thus, the occurrence of two PylRS enzymes in HMET1 appears not to be necessitated by an increased number of UAG codons in this genome.

### *Candidatus* Methanohalarchaeum thermophilum HMET1 encodes two distinct PylRS/tRNA^Pyl^ pairs

**Figure 2.**
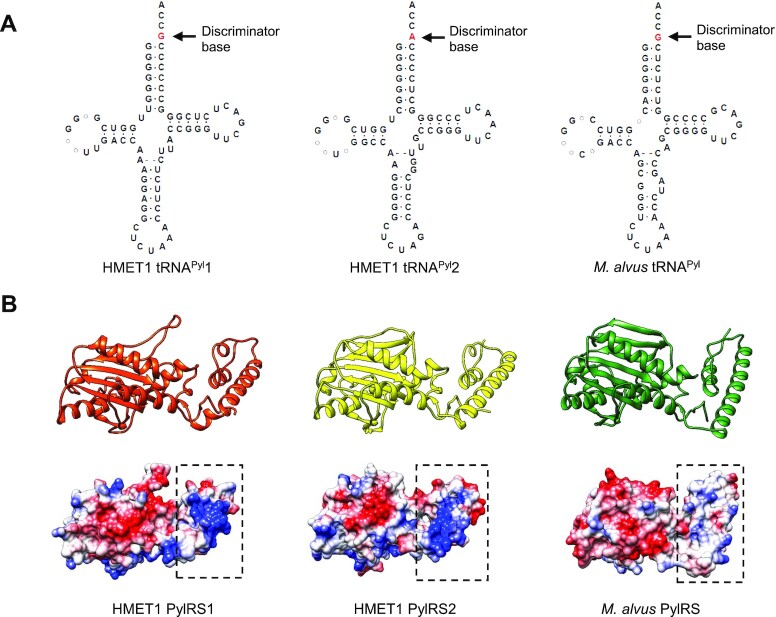
Prediction and structural analysis of the two HMET1 PylRS/tRNA^Pyl^ pairs. (**A**) Inferred cloverleaf structures of HMET1 tRNA^Pyl^1 and tRNA^Pyl^2 compared with the *M. alvus* tRNA^Pyl^. (**B**) 3D structural model of the two HMET1 PylRSs (all residues modeled at > 90% confidence) compared with the X-ray crystal structure of *M. alvus* PylRS (PDB 6JP2). Electrostatic potential of the selected PylRSs is represented by coulombic surface coloring with the unit of the potential colored in the range of values − 10 (red), 0 (white), and 10 (blue) kcal/mol · e using Chimera v 1.12.

Extension of our genomic analysis of *pylS* genes to the cognate tRNA^Pyl^ species led to the remarkable discovery, in the HMET1 strain, of two tRNA^Pyl^ (*pylT*) genes (Figure 2A), with different discriminator bases (position 73 of the tRNA) (2). Like all other known tRNA^Pyl^ species, HMET1 tRNA^Pyl^1 uses the discriminator base G, whereas HMET1 tRNA^Pyl^2 has A as discriminator base. The *pylT* genes are located adjacent to the *pylS* genes: *pylT1* and *pylT2* are immediately upstream of *pylS1* and *pylS2*, respectively (Figure 1). These *pylT* genes contain one or two introns with bulge-helix-bulge splicing motifs that presumably are removed during processing of the HMET1 pre-tRNA^Pyl^ (37,38) (Supplementary Figure S2). HMET1 tRNA^Pyl^1 resembles the canonical tRNA^Pyl^ except for the presence of an extra nucleotide between the D-arm and the anticodon arm (Figure 2A), similar to the tRNA^Pyl^ of methanogenic archaeon ISO4-G1 (39). In contrast, tRNA^Pyl^2 contains base substitutions in the variable loop, in addition to the noncanonical A73 (Figure 2A). Could PylRS1 with tRNA^Pyl^1 and PylRS2 with tRNA^Pyl^2 form cognate pairs?

PylRS1 and PylRS2 show 53% sequence identity; the predicted 3D structures of both enzymes are similar and closely resemble the catalytic domain of *Methanomethylophilus alvus* (*M. alvus*) PylRS and other enzymes of the Class II aaRS family as demonstrated by homology modeling (Figure 2B). HMET1, found in hypersaline environments, contains enzymes that often have reduced levels of hydrophobic residues (40). Not surprisingly, the surface of HMET1 PylRSs encompasses more charged amino acids compared with *M. alvus* PylRS (Figure 2B). Taken together, we discovered two novel PylRS/tRNA^Pyl^ pairs that naturally co-exist in the extremophile HMET1.

### Development of a facile system for *in vivo* characterization of the HMET1 PylRS/tRNA^Pyl^ pairs

To determine whether the PylRS/tRNA^Pyl^ pairs are functional *in vivo*, we first investigated the ability of PylRS2/tRNA^Pyl^2 pair to produce superfolder green fluorescent protein (sfGFP) from the *sfGFP(150TAG)* gene in the presence and in the absence of the non-proteinogenic amino acid BocK, a known substrate for many PylRS enzymes. Expression in *E. coli* by *Methanosarcina mazei* (*M. mazei*) and *M. alvus* PylRS/tRNA^Pyl^ pairs produces a large amount of soluble sfGFP (positive control), whereas expression of the HMET1 PylRS1/tRNA^Pyl^1 pair yielded only insoluble PylRS in the supernatant extract after cell lysis (Supplementary Figure S3). This finding is not surprising because halophilic enzymes often aggregate and misfold in low ionic conditions (41). Thus, functional characterization of HMET1 PylRS/tRNA^Pyl^ translation products in the well-established *E. coli* system is unfeasible.

**Figure 3.**
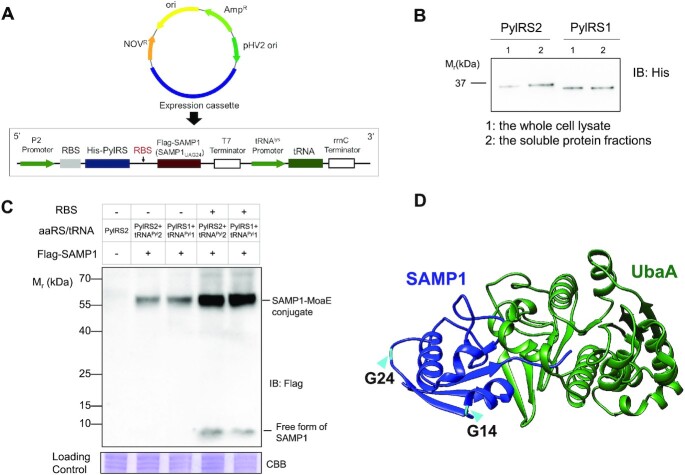
Heterologous protein expression in *H. volcanii*. (**A**) Schematic representation of the shuttle plasmid for co-expression of genes encoding PylRS/tRNA^Pyl^ pairs and SAMP1 protein. Promoters, terminators, and RBS are indicated. To estimate UAG suppression activity of HMET1 PylRS/tRNA^Pyl^ pairs, a SAMP1 gene with UAG as codon 24 (SAMP1_UAG24_) was utilized. (**B**) Soluble PylRS1 and PylRS2 proteins in the supernatant extract after cell lysis were detected via anti-His tag antibody immunoblotting (IB). (**C**) The expression of the wild-type Flag-SAMP1 was detected via anti-Flag antibody IB in cell lysate. The lower band corresponds to the full-length SAMP1 that is not conjugated with other proteins and the upper band corresponds to the SAMP1-MoaE conjugate. (**D**) The predicted structural model of SAMP1-UbaA complex based on the crystal structure of a bacterial MoeB-MoaD complex (1JW9). The glycine 14 and 24 residue (cyan) of the wild-type SAMP1 (PDB 3PO0) for site-specific incorporation of ncAAs are indicated. Experiments were performed in duplicate, and representative images are shown.

Therefore, we decided to use *H. volcanii*, a model halophilic euryarchaeon (42), as the chassis for functional characterization of the two PylRS/tRNA^Pyl^ pairs from HMET1. A system intended for genetic code expansion in *H. volcanii* requires: (i) high level expression of the PylRS/tRNA^Pyl^ pair; and (ii) a reporter protein that can be readily detected for measuring the BocK incorporation in response to an in-frame UAG stop codon. We developed a plasmid-based cassette for co-expressing the PylRS/tRNA^Pyl^ pair and a small archaeal ubiquitin-like modifier protein (SAMP1) as the reporter in *H. volcanii* (Figure 3A). The reasons for the choice of SAMP1 were: (i) SAMP1 is small and a homolog of ubiquitin, which has often been utilized as the reporter protein for genetic code expansion experiments (43), (ii) the known structure of SAMP1 can guide us to select permissive sites for BocK incorporation; (iii) only full-length SAMP1 can modify protein substrates, such as MoaE (the large subunit of molybdopterin synthase), and the SAMP1 conjugate can be readily detected by immunoblotting (44). In contrast, unsuccessful UAG suppression leads to truncated SAMP1 protein, which is unable to modify cellular substrates. Immunoblotting was utilized to estimate UAG suppression of the reporter protein.

For expression of tRNA^Pyl^ in *H. volcanii*, we placed the tRNA coding sequence and 15 nt each of upstream and downstream flanking sequences under control of the constitutive tRNA^Lys^ promoter (45). A polycistronic expression system driven by a strong constitutive promoter was utilized for producing PylRS and the reporter protein SAMP1 (Figure 3A). The recombinant PylRS protein with an N-terminal 6xHis-tag expressed well in *H. volcanii* and was soluble in the supernatant extract after cell lysis (Figure 3B). In order to increase the expression level of the *samp1* gene, a ribosome binding site (RBS) sequence was inserted ahead of the SAMP1 coding sequence (46), resulting in substantially increased abundance of free SAMP1 protein as well as its major conjugated form (SAMP1-MoaE) (Figure 3C).

Next, we sought to determine the permissive site(s) in the SAMP1 protein for site-specific incorporation of BocK. Our structural modeling showed that Gly24 is away from the SAMP1-UbaA (the *H. volcanii* ubiquitin-activating E1 enzyme homolog) interface, and Gly24 is located in the solvent-exposed loop (Figure 3D). Thus, a SAMP1 gene with UAG as codon 24 (SAMP1_UAG24_) provides the reporter protein SAMP1BocK24 where BocK should have a minimal impact on the structure and function of SAMP1. Because SAMP1 was mainly in the conjugated form under our culture conditions (Figure 3C), we evaluated the *in vivo* activity of the HMET1 PylRS/tRNA^Pyl^ pairs by measuring the protein abundance of the SAMP1_UAG24_-MoaE conjugate in the following experiments.

### Both HMET1 PylRS/tRNA^Pyl^ pairs are active and orthogonal in *H. volcanii*

**Figure 4.**
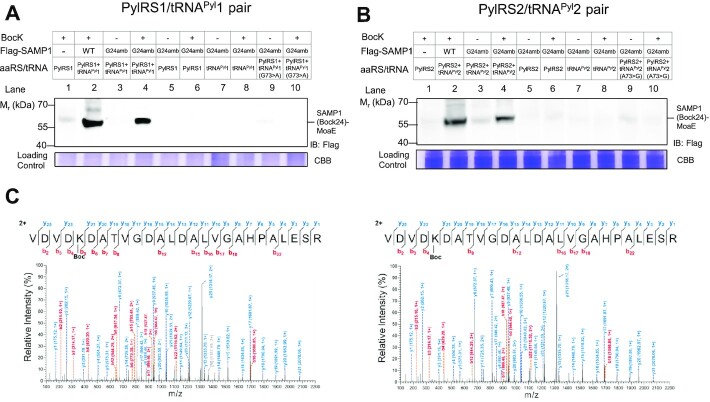
Both HMET1 PylRS/tRNA^Pyl^ pairs are active and orthogonal in *H. volcanii*. (**A-B**) Activity and orthogonality tests of HMET1 PylRS/tRNA^Pyl^ pairs in the presence and absence of BocK when compared to the abundance of wild-type SAMP1-MoaE conjugate formed under the same conditions. SAMP1-MoaE and SAMP1Bock24-MoaE conjugates were detected by anti-Flag antibody immunoblotting in cell lysate. Experiments were performed in triplicates, and representative images are shown. (**C**) High mass accuracy tandem mass spectrometry MS/MS unambiguously confirmed site-specific incorporation of BocK mediated by PylRS1/tRNA^Pyl^1 (left) and PylRS2/tRNA^Pyl^2 (right). Representative MS/MS spectra of BocK-containing peptides derived by collision-induced dissociation of the doubly charged precursor after trypsin digestion of the purified SAMP1Bock24-MoaE conjugate. The y-ion (colored blue) and b-ion (colored red) series detected are indicated.

In order to test whether the two HMET1 PylRS/tRNA^Pyl^ pairs are functional and orthogonal in *H. volcanii*, we transformed plasmids encoding the PylRS/tRNA^Pyl^ pairs and SAMP1_UAG24_, and cultured the cells in the presence or in the absence of 1 mM BocK. The ability to form UAG suppressor BocK-tRNA^Pyl^ was assayed by monitoring the production of the SAMP1Bock24-MoaE conjugate via anti-Flag immunoblotting. The results demonstrated that both HMET1 PylRS/tRNA^Pyl^ pairs facilitated efficient amber suppression as assessed by comparing the abundance of the produced SAMP1-MoaE conjugate to that formed by the wild-type under the same conditions (Figure 4A-B, lanes 4 versus 2). We observed minimal UAG readthrough by HMET1 PylRS/tRNA^Pyl^ pairs in the absence of BocK (Figure 4A-B, lane 3) or when the HMET1 PylRS or tRNA^Pyl^ were omitted (Figure 4A-B, lanes 5 to 8). Thus, both HMET1 PylRS/tRNA^Pyl^ pairs are orthogonal with respect to the aaRS enzymes and tRNA species endogenously present in *H. volcanii*.

To further validate BocK incorporation, the reporter protein was analyzed with LC-MS/MS. The SAMP1BocK24 and SAMP1Bock24-MoaE conjugate were purified by immunoprecipitation, and they represent the two major bands on the SDS-PAGE gel (Supplementary Figure S4). The conjugate band was extracted from the gel and then digested by trypsin and analyzed with tandem MS. A series of b and y ions unambiguously indicate that BocK was incorporated at the UAG-specified position 24 (Figure 4C). This result confirms that native HMET1 PylRS/tRNA^Pyl^ pairs incorporate BocK into proteins via genetic code expansion in *H. volcanii*.

The discriminator base G73 is a major tRNA^Pyl^ identity element (6). Because HMET1 tRNA^Pyl^1 contains the canonical G73 while tRNA^Pyl^2 carries A73, we tested the effect of swapping the N73 base (between tRNA^Pyl^1 and tRNA^Pyl^2) on UAG suppression. The G73A mutation in tRNA^Pyl^1 decreased suppression efficiency markedly (Figure 4A, lanes 9–10), which is consistent with a previous result using a G73A *Methanosarcina barkeri* tRNA^Pyl^ variant that reduced suppression efficiency by ≈ 70% (6). Interestingly, the A73G mutation in tRNA^Pyl^2 also led to a drastic decrease in amber suppression efficiency (Figure 4B, lanes 9–10). These findings support the notion that PylRS1 with tRNA^Pyl^1, and PylRS2 with tRNA^Pyl^2 are cognate pairs and that the nature of the discriminator base alone determines the mutual orthogonality of tRNA^Pyl^1 and tRNA^Pyl^2 (Figure 1).

### The two HMET1 PylRS/tRNA^Pyl^ pairs are mutually orthogonal

**Figure 5.**
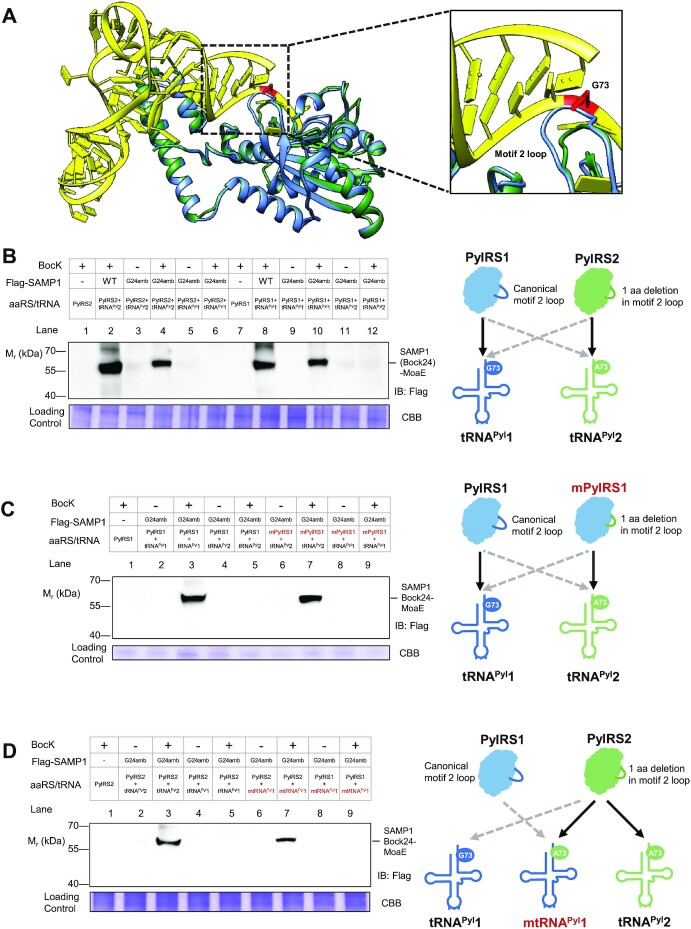
The A73 discriminator of tRNA^Pyl^2 and a shorter motif 2 loop in PylRS2 determine the mutual orthogonality between the two HMET1 PylRS/tRNA^Pyl^ pairs. (**A**) 3D structural models of HMET1 PylRS1 and PylRS2 (blue and green) in complex with *Desulfitobacterium hafniense* tRNA^Pyl^ (yellow). Magnified view shows the recognition of the CCA terminus by the motif 2 loop. (**B-D**) Production of SAMP1Bock24-MoaE conjugate was mediated by cognate, non-cognate and engineered PylRS/tRNA^Pyl^ pairs analyzed with anti-Flag immunoblotting (IB) in cell lysate. mPylRS1 (PylRS1 with motif 2 loop from PylRS2) and mtRNA^Pyl^1 (tRNA^Pyl^1 with G73A mutation) are labelled red. Migration of SAMP1Bock24-MoaE conjugate is indicated. Schematic on the right summarizing the activity and functional orthogonality between different cognate, non-cognate and engineered HMET1 PylRS/tRNA^Pyl^ pairs. Black arrows indicate high activity, and dashed grey arrows indicate minimal activity. Experiments were performed in triplicates, and representative images are shown. See Materials and Methods for details.

To better understand the role of the A or G discriminator base in tRNA^Pyl^1 and tRNA^Pyl^2, respectively, we analyzed the predicted structures of PylRS1 and PylRS2 superimposed onto the crystal structure of the *Desulfitobacterium hafniense* PylRS-tRNA^Pyl^ complex (47). We were especially interested in the aaRS–tRNA interaction surface close to the discriminator base, focusing on the enzyme's motif 2 loop that is proximal to N73 and plays an essential role in the recognition of the tRNA CCA terminus. To our surprise, we found that the PylRS2 motif 2 loop is shorter than that of PylRS1 (Figure 5A).

Next, we aimed to study whether the two PylRS/tRNA^Pyl^ pairs are mutually orthogonal in their aminoacylation specificity when expressed in *H. volcanii*. We swapped the PylRS and tRNA^Pyl^ coding sequences between expression vectors producing PylRS/tRNA^Pyl^ pairs and the SAMP1BocK24 reporter protein. Minimal readthrough of the amber codon was observed when the HMET1 PylRS paired with its non-cognate tRNA^Pyl^ in the presence or in the absence of BocK (Figure 5B, lanes 5–6 and 11–12). This result demonstrates that these two HMET1 PylRS/tRNA^Pyl^ pairs are mutually orthogonal in *H. volcanii*. To further validate this result, we rationally transplanted each unique feature (A73 or shorter motif 2 loop) from the PylRS2/tRNA^Pyl^2 pair to the PylRS1/tRNA^Pyl^1 pair. The PylRS1 with the short motif 2 loop from PylRS2 and tRNA^Pyl^1 with G73A mutation are designated as mPylRS1 and mtRNA^Pyl^1, respectively (Figure 5C-D). We compared the production of the SAMP1BocK24-MoaE conjugate by these engineered PylRS/tRNA^Pyl^ pairs to that mediated by HMET1 cognate PylRS/tRNA^Pyl^ pairs. Surprisingly, mPylRS1/tRNA^Pyl^2 and PylRS2/mtRNA^Pyl^1 pairs showed UAG suppression efficiency comparable to that of HMET1 cognate PylRS1/tRNA^Pyl^ pairs (Figure 5C-D, lanes 7 versus 3). In contrast, no SAMP1Bock24-MoaE conjugate was detected in the presence of engineered mPylRS1/tRNA^Pyl^1 and PylRS1/mtRNA^Pyl^1 pairs (Figure 5C-D, lane 9). These results demonstrate that the shorter motif 2 loop in PylRS2 combined with the discriminator base A73 in tRNA^Pyl^2 determine the mutual orthogonality of the two PylRS/tRNAPyl pairs in HMET1.

### Motif 2 loop length of PylRS2 determines its ability to acylate tRNA^Pyl^2

**Figure 6.**
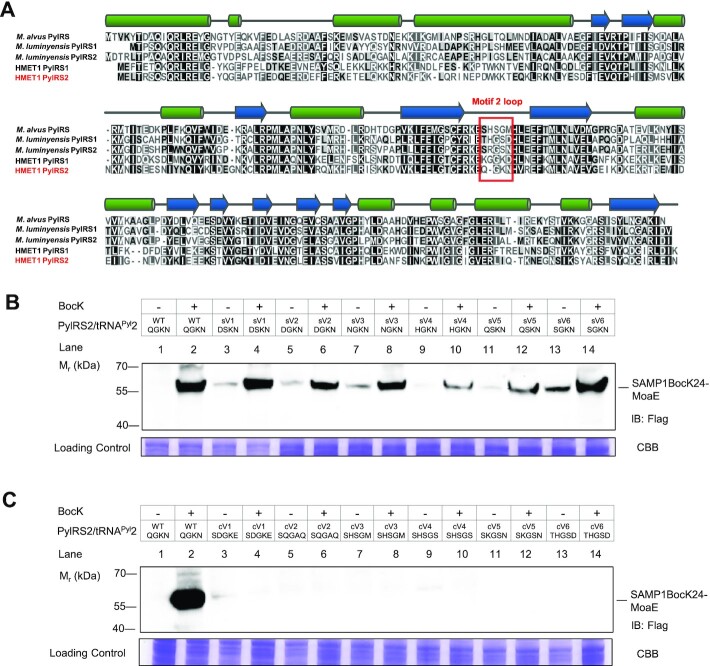
Motif 2 loop length of PylRS2 determines its ability to acylate tRNA^Pyl^2. (**A**) Multiple amino acid sequence alignment of ΔPylSn enzymes from HMET1, *M. luminyensis* B10 and *M. alvus*. The predicted secondary structure elements including α-helices, β-sheets and random coils are indicated above the PylRS sequence. Residues corresponding to the motif 2 loop of PylRS are highlighted in red. (**B**) Production of SAMP1Bock24-MoaE conjugate by PylRS2 variants (sV1-6) co-expressed with HMET1 tRNA^Pyl^2 analyzed with anti-Flag IB in cell lysate. The sV1-6 are PylRS2 variants with a shortened motif 2 loop (one amino acid deletion). (**C**) Production of SAMP1Bock24-MoaE conjugate by PylRS2 variants (cV1-6) co-expressed with HMET1 tRNA^Pyl^2 analyzed with anti-Flag IB in cell lysate. The cV1-V6 are PylRS2 variants with the full-length canonical motif 2 loop as found in other six ΔPylSn enzymes from *M. mazei*, *D. hafniense*, *M. alvus*, *Candidatus* Methanoplasma termitum MpT1, *Candidatus* Methanomassiliicoccus intestinalis Issoire-Mx1 and *Methanomassiliicoccus luminyensis* B10. The amino acid sequence of motif 2 loop in PylRS2 variants (sV1-6 and cV1-6) is shown in the table on top of the immunoblot images. Migration of SAMP1Bock24-MoaE conjugate is indicated. Experiments were performed in duplicate, and representative images are shown. See Materials and Methods for details.

To better understand the relationship of the short motif 2 loop in PylRS2 and the discriminator base A73 in tRNA^Pyl^2, we constructed and examined a multiple amino acid sequence alignment of HMET1 PylRS1 and PylRS2 with the other known C-terminal domain-only PylRS enzymes. The most remarkable observed difference was that HMET1 PylRS2 contains a shorter (by one amino acid residue) motif 2 loop (Figure 6A). Next, we sought to demonstrate that this single amino acid deletion accounts for its activity towards tRNA^Pyl^2. We constructed a series of PylRS2 variants with a shortened motif 2 by rational design (sV1-sV6). We also generated a series of PylRS2 variants with the full-length canonical motif 2 loop as found in other PylRSs (cV1-6). We compared the production of the SAMP1BocK24-MoaE conjugate by these engineered PylRS/tRNA^Pyl^ pairs to that mediated by the wild-type PylRS2/tRNA^Pyl^2 pair. All PylRS2 variants (sV1-6) with one amino acid deletion in the motif 2 loop could still function with tRNA^Pyl^2, although these PylRS2/tRNA^Pyl^ pairs varied in UAG suppression efficiency (Figure 6B, lanes 4, 6, 8, 10, 12 and 14). In contrast, we observed minimal amber codon readthrough by engineered PylRS/tRNA^Pyl^ pairs consisting of ‘full-length’ PylRS2 variants (cV1-6) and tRNA^Pyl^2 in the presence of BocK (Figure 6C, lanes 4, 6, 8, 10, 12 and 14), compared to the wild-type PylRS2/tRNA^Pyl^2 pair (Figure 6C, lane 2). These findings suggest that the length of motif 2 loop in PylRS2 determines the capacity of these enzyme variants to acylate the non-canonical tRNA^Pyl^2 (with the discriminator base A73).

In the absence of BocK, the UAG readthrough efficiency of SAMP1_UAG24_ by tRNA^Pyl^2 and PylRS variants (sV1-6) was low, but non-negligible (Figure 6B, lanes 3, 5, 7, 9, 11 and 13). To determine the nature of the mis-incorporated canonical amino acids, the relevant SAMP1-MoaE conjugate products formed by the PylRS2 variants sV1 and sV6 (Figure 6B, lanes 3 and 13, respectively) were isolated and analyzed by LC-LC/MS. Lys and Gln were found to be mis-incorporated when PylRS2 variant sV1 (DSKN) was used (Supplementary Figure S5), and Glu, Lys and Gln were identified using PylRS2 variant sV6 (SGKN) (Supplementary Table S5 and Supplementary Figure S6). To answer the question whether these mis-incorporated amino acids are carried by tRNA^Pyl^2 to the ribosome, we undertook an experiment in the absence of HMET1 tRNA^Pyl^2. Only minimal UAG readthrough was observed with PylRS2 variants (sV1-sV6) in the presence or in the absence of BocK (Supplementary Figure S7A and Supplementary Figure S7B, lanes 9–14 versus lanes 3–8). Thus, it appears that tRNA^Pyl^2 is mainly responsible for the observed low level misincorporation. As shown above, the PylRS2/tRNA^Pyl^2 pair is completely orthogonal with respect to the endogenous *H. volcanii* aaRSs and tRNAs (Figure 5B and Supplementary Figure S7B lane1); thus, we suggest that engineering of the PylRS2 motif 2 could impair binding to tRNA^Pyl^2, thus allowing endogenous aaRSs to charge canonical amino acids (e.g. Lys or Gln) onto tRNA^Pyl^2.

### Encoding two distinct ncAAs into one protein by the mutually orthogonal HMET1 PylRS/tRNA^Pyl^ pairs

**Figure 7.**
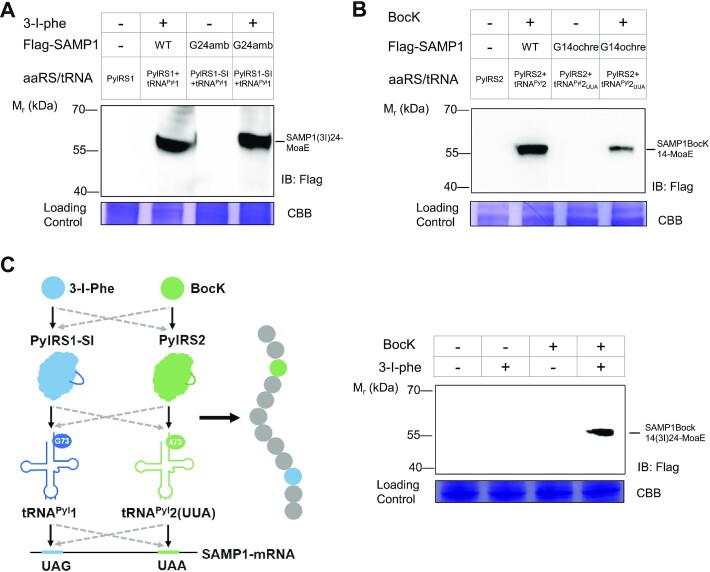
Encoding two distinct ncAAs using mutually orthogonal HMET1 PylRS/tRNA^Pyl^ pairs. (**A**) Production of SAMP1(3I)24-MoaE conjugate by PylRS1-SI/tRNA^Pyl^1 pair analyzed by anti-Flag immunoblotting (IB) in cell lysate. (**B**) Production of the SAMP1BocK14-MoaE conjugate by PylRS2/tRNA^Pyl^2_UUA_ pair analyzed by anti-Flag IB in cell lysate. (**C**) Detection of purified SAMP1(3I)14Bock24-MoaE conjugate produced by PylRS1-SI/tRNA^Pyl^1 and PylRS2/tRNA^Pyl^2_UUA_ pairs. Total 100 ml *H. volcanii* cells expressing the one-plasmid system were grown in the presence and absence of 3-I-phe and BocK followed by the anti-Flag immunoprecipitation to enrich the SAMP1(3I)14Bock24-MoaE conjugate. Migration of SAMP1(3I)14Bock24-MoaE conjugate is indicated. The same amount of cellular proteins were utilized for immuno-enrichment of Flag-tagged proteins confirmed by Coomassie blue staining of the input. Experiments were performed in duplicates, and representative images are shown. See Materials and Methods for details.

As a demonstration of the orthogonality of the two PylRS/tRNA^Pyl^ systems, we decided to incorporate two distinct ncAAs into a single polypeptide. To achieve this goal, engineering of the amino acid-binding pocket of PylRS1 and PylRS2 for selective incorporation of two different ncAAs, and construction of a tRNA^Pyl^ species able to suppress UAA or UGA were required. The repertoire of ncAAs that can be acylated to tRNA^Pyl^ by PylRS variants has been greatly expanded in recent years so that PylRS variants exist that are selective for several ncAA substrates but exclude others (5). For instance, in previous work, we constructed a highly active PylRS mutant (IFRS; Asn346Ser/Cys348Ile) that is selective for 3-iodo-l-Phe (3-I-Phe) but rejects BocK (48). From the alignment of the amino acid-binding pockets of HMET1 PylRS1 and IFRS, we identified Asn163 and Val165 in PylRS1 as the residues corresponding to Asn346 and Cys348 in IFRS, and introduced the corresponding Asn163Ser and Val165Ile amino acid substitutions into HMET1 PylRS1 (PylRS1-SI). The PylRS1-SI/tRNA^Pyl^1 pair exhibited strong amber suppression in a 3-I-Phe-dependent manner as measured by the production of a SAMP1(3I)24-MoaE conjugate (Figure 7A). Given that PylRS lacks anticodon recognition (6) and our PylRS/tRNA^Pyl^ pairs would decode diverse codons (49), we next mutated the tRNA^Pyl^2 anticodon to generate tRNA^Pyl^2_UUA_ which would facilitate BocK incorporation into SAMP1 at the position directed by the UAA codon. Based on the analysis of the SAMP1 structure, the Gly14 codon was mutated to UAA for BocK incorporation (Figure 3D). We transformed *H. volcanii* with plasmids encoding the HMET1 PylRS2/tRNA^Pyl^2_UUA_ pair and SAMP1(_UAA14_), and then cultured the cells in the presence or absence of 1 mM BocK. The PylRS2/tRNA^Pyl^2_UUA_ pair exhibited ochre suppression in a BocK-dependent manner (Figure 7B). Only a small amount of SAMP1BocK14-MoaE conjugate was formed by the PylRS2/tRNA^Pyl^2_UUA_ pair, which is consistent with the previous finding that UAA/UGA mediated suppression is inefficient in *H. volcanii* (45). Thus, we now developed PylRS/tRNA^Pyl^ pairs for site-specific incorporation in *H. volcanii* of 3-I-Phe and BocK directed by UAG and UAA codons, respectively.

To demonstrate simultaneous incorporation of 3-I-Phe and BocK into a single protein in response to two distinct codons in *H. volcanii*, we chose a one-plasmid system encoding two copies of SAMP1_UAA14, UAG24_ and one copy of PylRS1-SI/tRNA^Pyl^1 and PylRS2/tRNA^Pyl^2_UUA_ (Supplementary Figure S8). *H. volcanii* cells expressing the one-plasmid system were grown in the presence or absence of 3-I-Phe and BocK followed by the anti-Flag immunoprecipitation to enrich the reporter proteins. Production of a SAMP1(3I)14Bock24-MoaE conjugate was dependent on the addition of both ncAAs (Figure 7C). Further LC-MS/MS analysis of the purified reporter protein confirmed that BocK and 3-I-Phe were incorporated at the TAA and TAG-directed positions respectively (Supplementary Figure S9). This result demonstrates that the HMET1 PylRS/tRNA^Pyl^ pairs function in the same heterologous *H. volcanii* cell to incorporate distinct ncAAs into a single polypeptide programmed by different stop codons. Thus, mutual orthogonality, most likely, was the original property of these PylRS/tRNA^Pyl^ pairs in HMET1.

A different scenario has been reported previously where PylRS/tRNA pairs from different archaea were shown to be mutually orthogonal based on their tRNA recognition mode. This allowed the rational design of two orthogonal PylRS/tRNA pairs with distinct amino acid specificities that can operate together in a host cell (13,17).

## DISCUSSION

### Potential applications of HMET1 PylRS/tRNA^Pyl^-derived pairs in archaea

A variety of PylRS/tRNA^Pyl^-derived pairs have been developed in bacteria and eukaryotic cells to incorporate useful Pyl derivatives into proteins of interest (50), enabling many important applications including identification of weak protein-protein interactions in living cells (51), generation of physiologically relevant proteins with authentic post-translational modifications (e.g. acetylation) (52), and protein labelling for cellular imaging (53). However, genetic incorporation of ncAA into protein mediated by engineered aaRS/tRNA pairs in archaea, the third domain of life, has never been reported before. As shown in this work, it is facile to generate designated PylRS/tRNA^Pyl^ pairs in *H. volcanii* by transplanting previously reported mutations into HMET1 PylRS/tRNA^Pyl^ pairs. We envision HMET1 PylRS/tRNA^Pyl^-derived pairs could be further engineered and optimized to enrich the toolbox for dissecting fundamental biological processes in archaea.

Halophilic enzymes are of major interest to many industrial applications as they serve as efficient catalysts to produce biomaterials and biofuels in low water activity conditions (54). For instance, halophilic pyrophosphatase displays superior catalytic activity at high concentrations of organic solvents (55). However, expression of halophilic enzymes in heterologous hosts, such as *E. coli*, is often problematic because these proteins tend to misfold and aggregate in a low ionic strength environment. Here we demonstrate that *H. volcanii* can function as a facile chassis to characterize novel enzymes, discovered by mining metagenomic and microbial genomic data, from halophiles that are usually difficult or impossible to culture. In particular, novel aaRS candidates identified in the microbial dark matter (56,57) could be good targets. Furthermore, the ability to genetically encode ncAAs has greatly expanded structural and functional repertoire of proteins. Examples include catalytic efficiency and thermal stability of proteins improved by genetic code expansion (58–60). Thus, introducing ncAAs into proteins of interest in *H. volcanii in vivo* using the HMET1 PylRS/tRNA^Pyl^-derived pairs can be expected to open a new avenue for halophilic protein engineering and biotechnological applications.

### Why have two PylRS/tRNA^Pyl^pairs in the same organism?

A simple argument suggests that two *ΔPylSn* gene copies could be needed for efficient translation of in-frame UAG stop codons with Pyl in the relevant genes in the HMET1 genome. However, the data in Supplementary Figure S1 indicate that this is not the case because the HMET1 genome contains an average number of such genes compared with the seven other archaea that only possess a single *ΔPylSn* copy. These two PylRS enzymes might not be expressed under the same growth conditions, but rather, in response to different environmental cues, ensuring the presence of at least one active PylRS/tRNA^Py^ pair at all times during the growth cycle. The fact the HMET1 PylRS/tRNA^Pyl^ pairs are mutually orthogonal is far more interesting. Although the translation of UAG codons with BocK suggests that both HMET1 PylRS enzymes recognize Pyl, their *in vivo* amino acid specificities in HMET1 cells remain to be determined biochemically. The possibility remains that one HMET1 PylRS/tRNA^Pyl^ pair evolved a different amino acid specificity, necessitating the simultaneous presence of the two mutually orthogonal pairs.

#### Which of the two PylRS/tRNA^Pyl^ is ancestral?

The duplication of the *ΔPylSn* gene in HMET1, followed by the emergence of two orthogonal ΔPylSn/tRNA^Pyl^ pairs, is so far unique among the available genomes. The only detectable ΔPylSn protein in the current databases with a deletion in the motif 2 loop is KXB05636.1, a truncated PylRS with 62% sequence identity to HMET1 PylRS2 (61). This protein is encoded in a contig from a candidate division MSBL1 archaeon SCGC-AAA382A03 that also belongs to *Methanonatronarchaeia*, suggesting that the duplication event occurred at some point in the evolution of this class of archaea. Furthermore, the tree built using *pylT* genes does not show any major deviations from the *pylS* tree (Supplementary Figure S10), suggesting that *pylT* was a part of the duplicated region, evolving in parallel with the cognate PylRS (Figure 1). Comparison of the *pylS* gene neighborhoods (Supplementary Table S4) suggests that PylRS2 is the ancestral form because it is encoded in the vicinity of the pair of genes encoding the putative permease RhaT and an uncharacterized protein from arCOG04962 (Figure 1). The latter pair of tightly linked genes is present in similar neighborhoods in the majority of Thermoplasmatales genomes that contain a single *pylS* gene, so this gene arrangement is likely to be ancestral. If this is the case, the duplication of a large region encoding *pylS*, *pylB* and several other genes, including methylamine methyltransferases with an in-frame UAG codon, most likely, occurred before the deletion in the PylRS2 motif 2 loop. A similar independent duplication that occurred in the lineage of *M. luminyensis* B10 did not result in analogous changes in either ΔPylSn or the cognate tRNA^Pyl^. It remains to be determined whether the ΔPylSn/tRNA^Pyl^ pairs in this species are mutually orthogonal.

#### Why is the PylRS2/tRNA^Pyl^2 pair absent in the overwhelming majority of Pyl encoding organisms?

As mentioned above, apart from HMET1, only a candidate division MSBL1 archaeon SCGC-AAA382A03 (61) may have a PylRS2-like enzyme (with a 1 aa deletion). Likewise, the presence of a discriminator A73 tRNA^Pyl^ so far was detected only in these two organisms (61). Why did PylRS identity switch to a G73 discriminator base? A possible reason might be the increased substrate specificity conferred by the G73 discriminator base given that the majority of tRNAs use the A73 identity element. For PylRS enzymes (*e.g. Desulfitobacterium hafniense*) a G73→A73 switch lowers the catalytic efficiency ∼100 fold (62).

### tRNA identity and genetic code

The mutually orthogonal PylRS/tRNAPyl pairs in HMET1 provide the clearest example yet for the importance of a single base change in the discriminator position leading to a concurrent identity change.

The co-existence and natural orthogonality of the two HMET1 PylRS/tRNA pairs also offer unique insights into the prospect of a still expanding genetic code. Studies based on the conspicuous absence of the tRNA^Gly^_ACC_ species from eukaryotic genomes suggested that genetic code expansion halted due to functional and structural constraints in the tRNA structure that limit the effective use of newly evolved tRNA identities (63). However, the HMET1 PylRS/tRNA pairs showcase that new tRNA identities can emerge with minimal changes of a tRNA if an aaRS partner is able to adapt to those changes. Similar cases were recently reported for discriminator base variants of a methanogen tRNA^Cys^ coupled with a cysteinyl-tRNA synthetase variant with a mutated small loop (64), and for a *Streptomyces* tRNA with Ala anticodon that is exclusively acylated with Pro by a noncanonical prolyl-tRNA synthetase (65). Thus, simultaneous/tandem duplication of tRNA and aaRS genes might lead to orthogonal aaRS/tRNA pairs able to evolve novel amino acid specificities.

## DATA AVAILABILITY

The mass spectrometry raw data have been deposited to the ProteomeXchange Consortium under the dataset identifier PXD027053 as well as CNGB Sequence Archive (CNSA) of China National GeneBank DataBase (CNGBdb) with accession number CNP0001963.

## Supplementary Material

gkac271_Supplemental_File

## Notes

Present address: Qianqian Zhao, Department of Rheumatology and Immunology, Shenzhen People's Hospital, The Second Clinical Medical College of Jinan University, Shenzhen, 518020, China; Integrated Chinese and Western Medicine Postdoctoral Research Station, Jinan University, Guangzhou, 510632, China.

Present address: Takahito Mukai, Department of Life Science, College of Science, Rikkyo University, 3-34-1 Nishi-Ikebukuro, Toshima-ku, Tokyo 171-8501, Japan.

Present address: Kazuaki Amikura, Department of Interdisciplinary Space Science, Institute of Space and Astronautical Science, Japan Aerospace Exploration Agency, Kanagawa, Japan.
