# Supplementary material for: The tRNA discriminator base defines the mutual orthogonality of two distinct pyrrolysyl-tRNA synthetase/tRNAPyl pairs in the same organism
Source: Nucleic Acids Res. 2022 Apr 25;50(8):4601–15. doi: 10.1093/nar/gkac271 (PMC9071458; doi:10.1093/nar/gkac271)
Supplement: gkac271_Supplemental_File [file gkac271_supplemental_file.pdf]

# SUPPLEMENTARY INFORMATION

## The tRNA discriminator base defines the mutual orthogonality of two distinct pyrrolysyl-tRNA synthetase/tRNA$^{Pyl}$ pairs in the same organism

Haolin Zhang, Xuemei Gong, Qianqian Zhao, Takahito Mukai, Oscar Vargas-Rodriguez, Huiming Zhang Yuxing Zhang, Paul Wassel, Kazuaki Amikura, Julie Maupin-Furlow, Yan Ren, Xun Xu, Yuri I. Wolf, Kira S. Makarova, Eugene V. Koonin, Yue Shen, Dieter Söll, Xian Fu

Content:

**Supplementary Figures 1-10**

**Supplementary Table 1** ………………………………………………… p 1
**Supplementary Table 2** ………………………………………………… p 3
**Supplementary Table 3** ………………………………………………… p 6
**Supplementary Table 4** ………………………………………………… p 8
**Supplementary Table 5** …………………………………………………p 47
**Supplemental References** ……………………………………………....p 48
**Additional File 1 (includes original tree in Newick format)** …………p 48

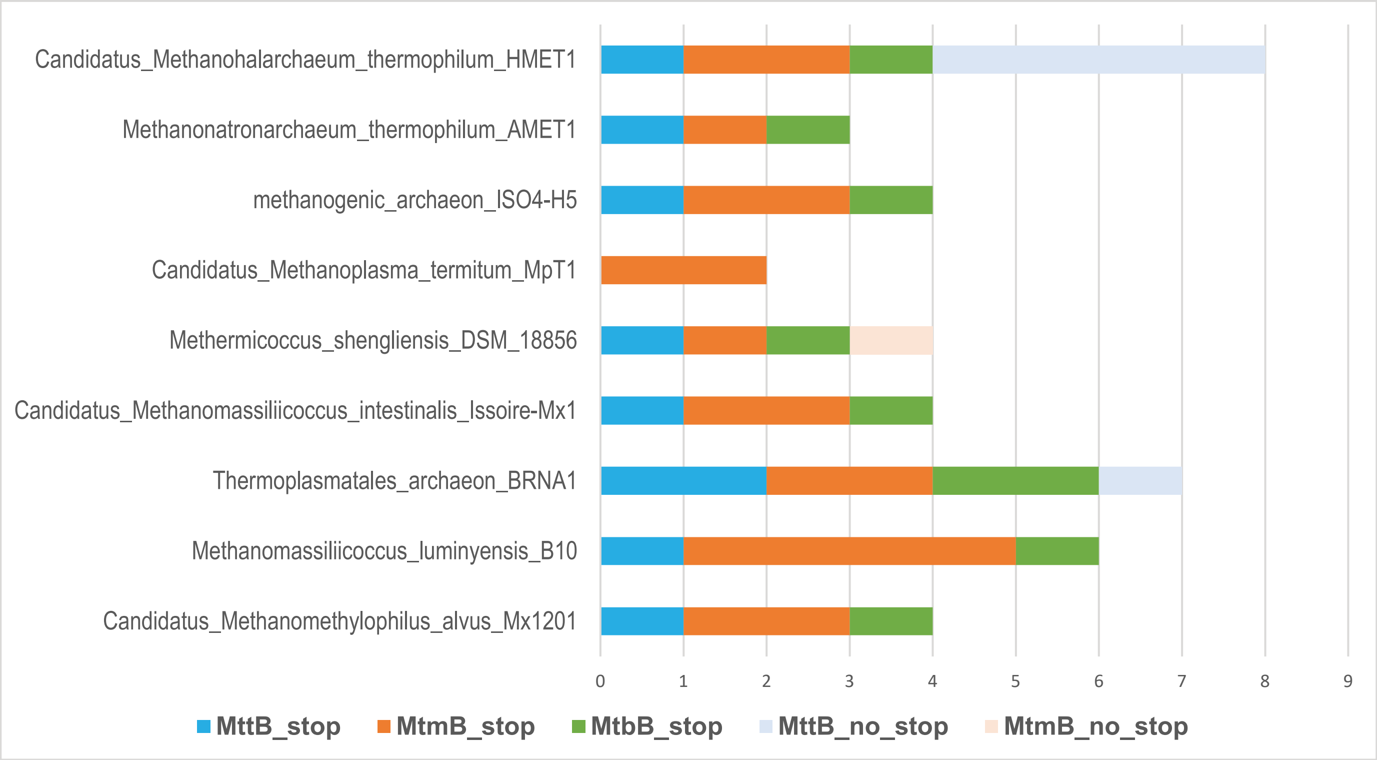


**Fig. S1.** The number of in-frame stop codons in methylamine methyltransferases in nine genomes with ΔPylSn genes. The MttB_stop (blue), MtmB_stop (orange) and MtbB_stop (green) represent the methyltransferase genes (mtmB, mtbB and mttB) that contain an in-frame stop codon. The MttB_no_stop (light blue) and MtmB_no_stop (light orange) represent the methyltransferase genes (mttB and mtmB) that contain no in-frame stop codon.

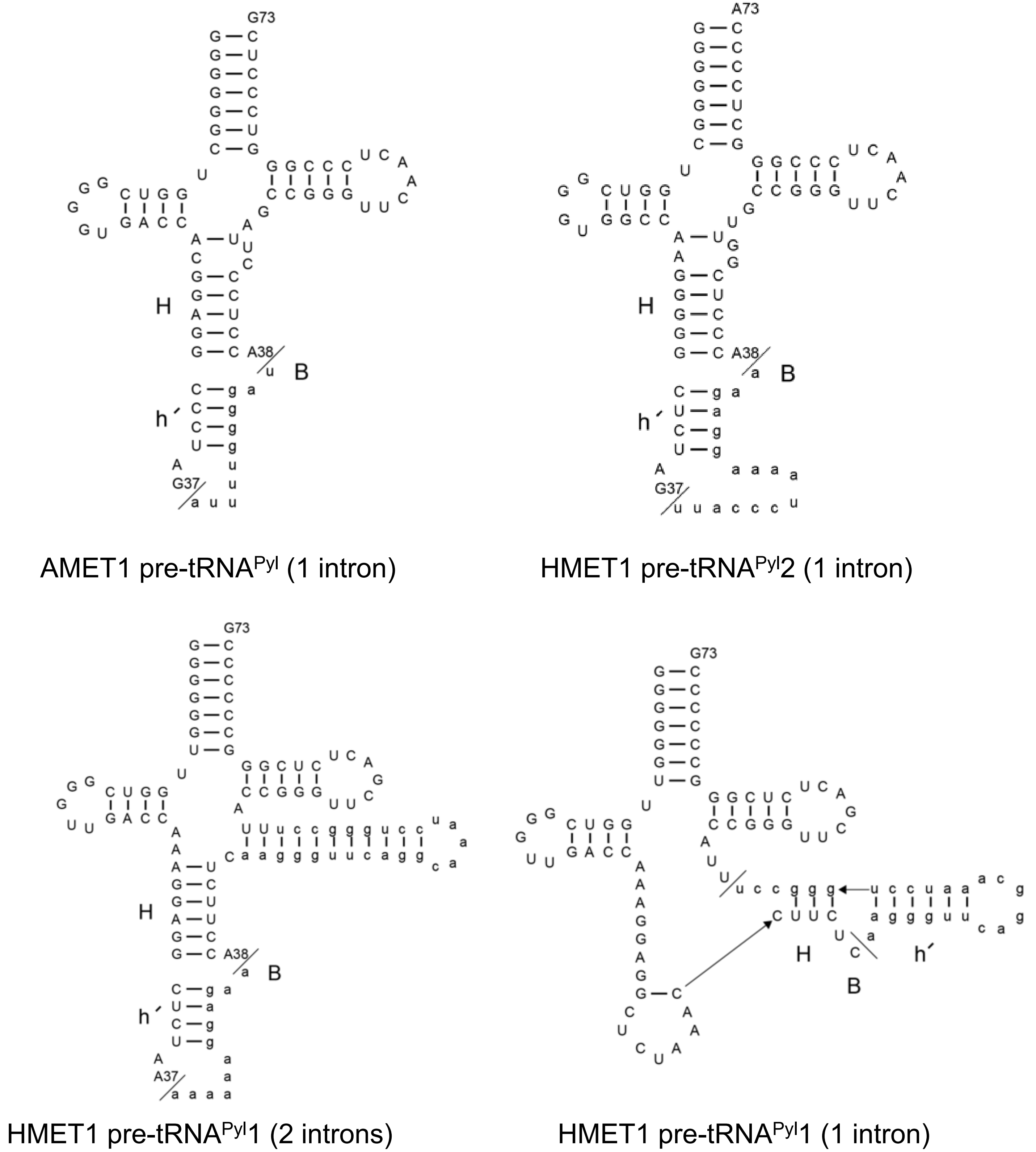


**Fig. S2.** Splicing motifs present in three haloarchaeal pre-$tRNA^{Pyl}$ species. These pre-tRNAs have a bulge-helix-bulge (BHB) type intron in the anticodon arm. In addition, the pre-$tRNA^{Pyl}$1 of HEMT1 was predicted to have a second intron. The nucleotides excised by tRNA-splicing endonucleases are indicated with small letters, while the splicing sites are indicated with diagonal lines.

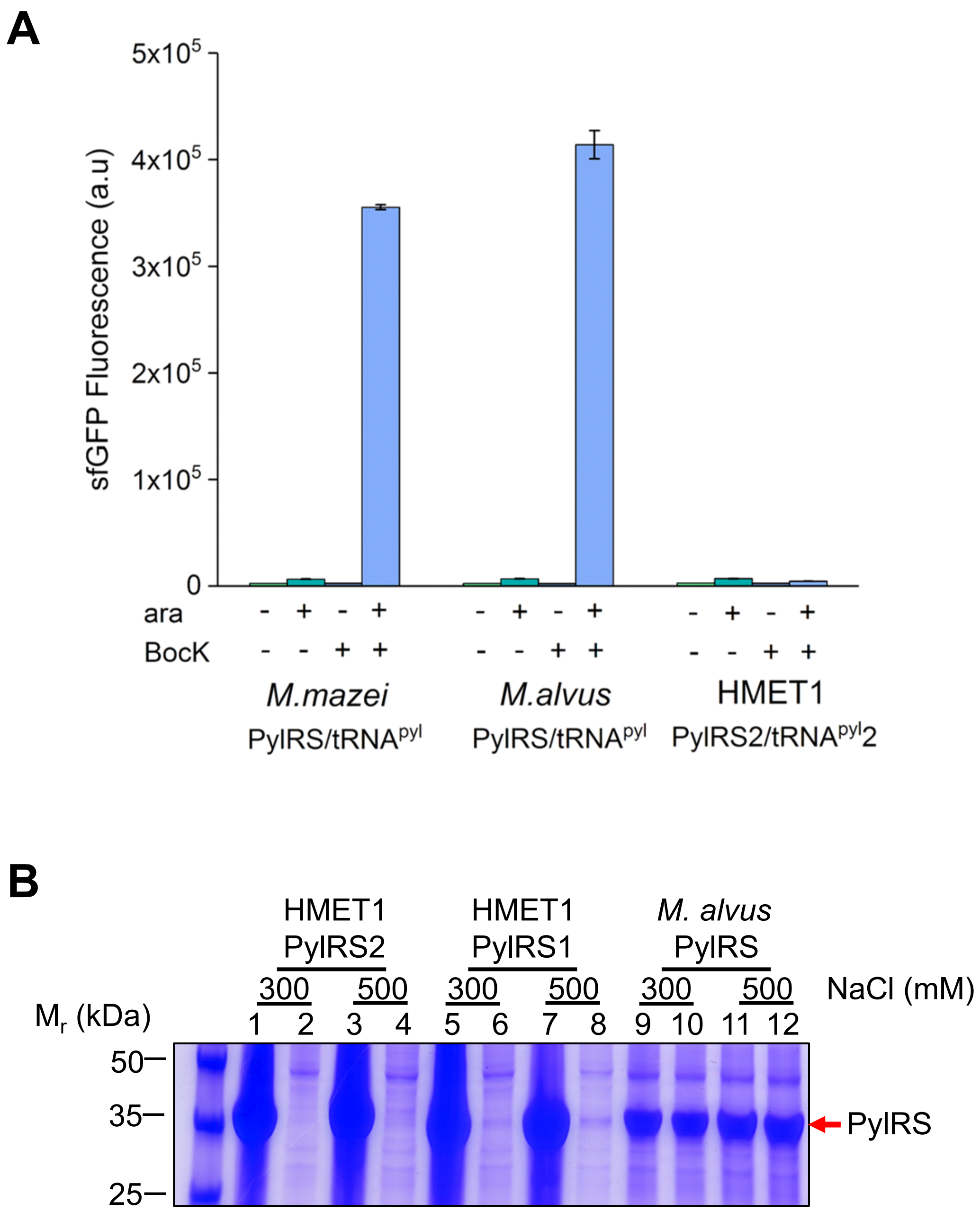


**Fig. S3.** Activity and solubility tests for HMET1 PylRS/tRNA$^{Pyl}$ pair in *Escherichia coli*. (A) Investigating the ability of PylRS2/tRNA$^{Pyl}$2 pair to produce superfolder green fluorescent protein (sfGFP) from the *sfGFP(150TAG)* gene in the presence and absence of BocK and arabinose; the error bars show the mean and ±s.d. of three biological replicates. (B) Solubility assay of PylRSs in *E. coli* BL21(DE3) expressing *plyS* gene in the pET28a plasmid; lane 1, 3, 5, 7, 9, 11 represent the whole cell lysate and lane 2, 4, 6, 8, 10, 12 represent supernatant extract.

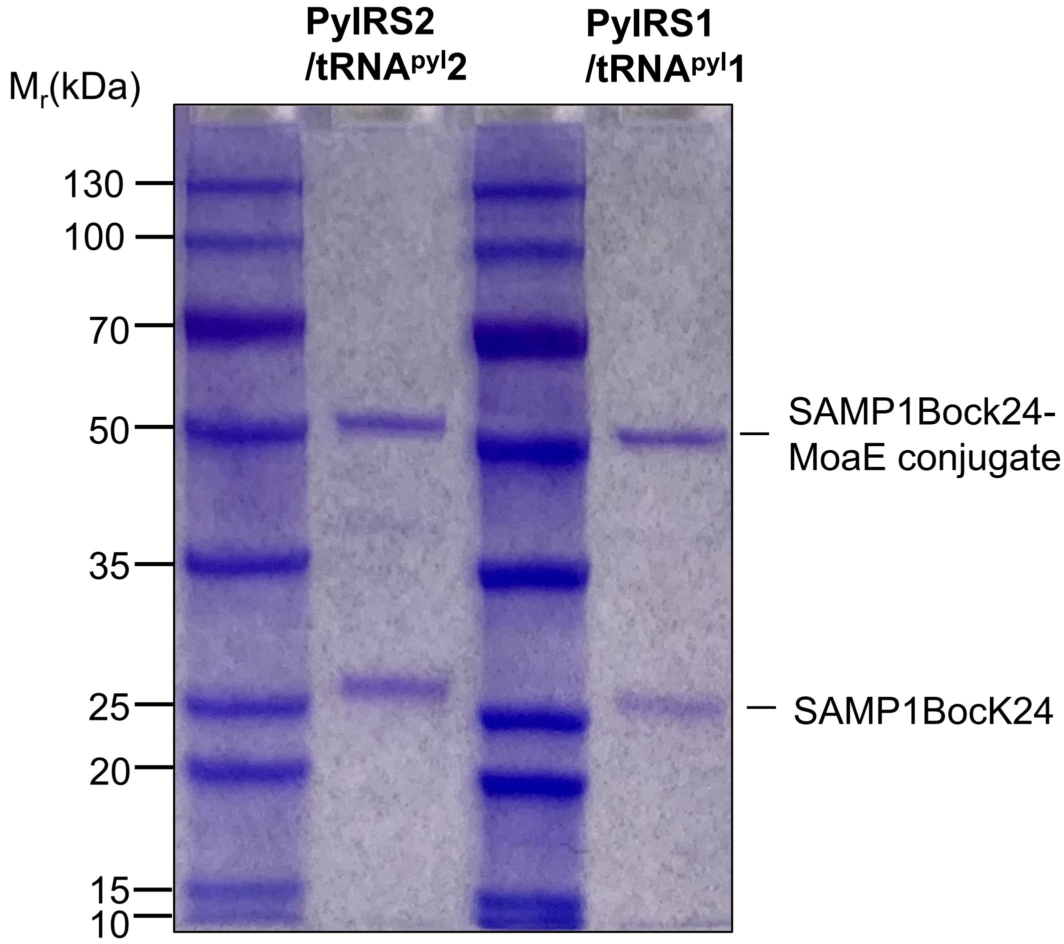


**Fig. S4.** SDS-PAGE profile of SAMP1BocK24 and SAMP1Bock24-MoaE conjugate purified by anti-Flag immunoprecipitation. The SAMP1Bock24-MoaE conjugate band migrating at 50 was cut for further analysis via tandem mass spectrometry.

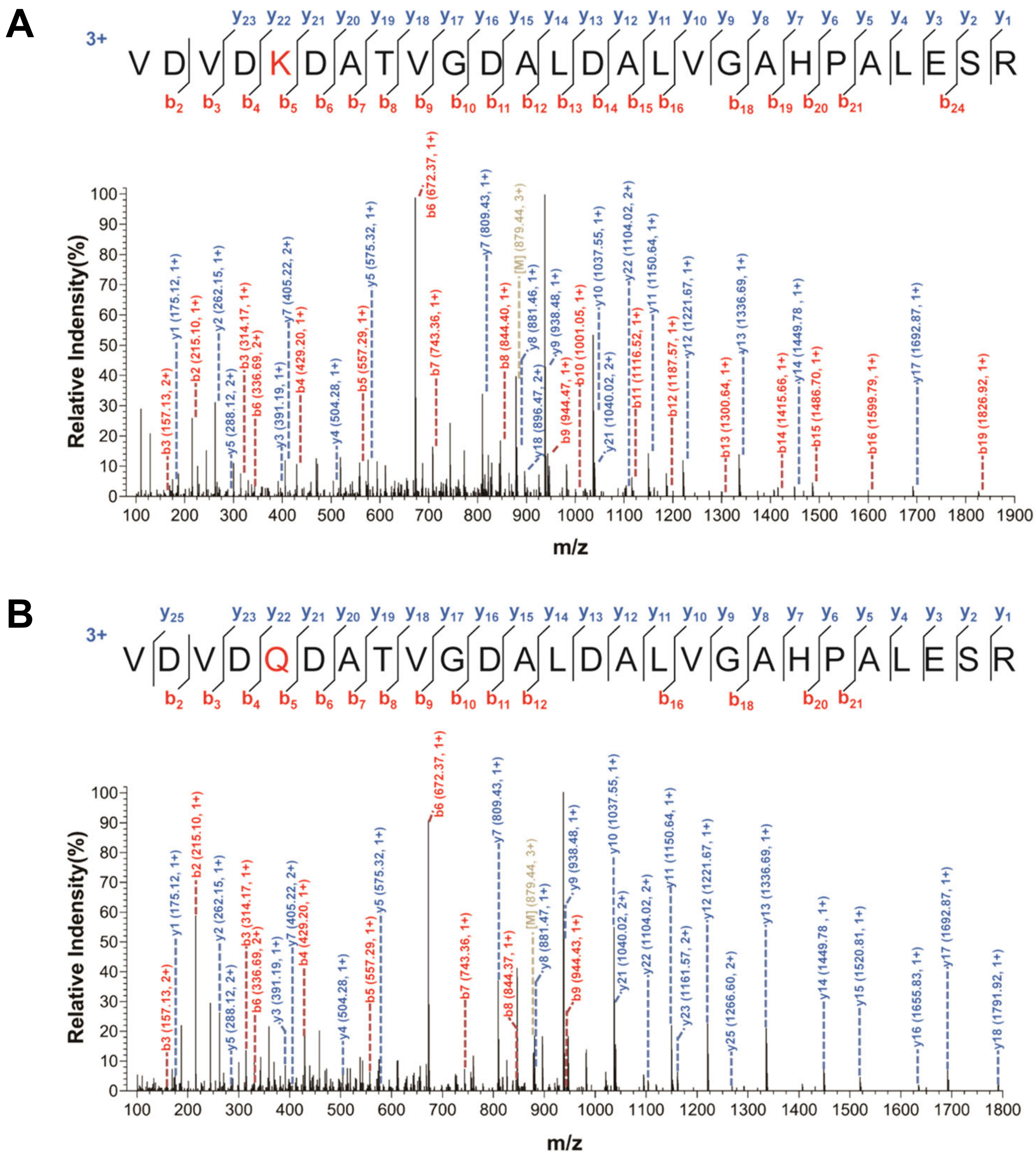


**Fig. S5.** Representative MS/MS spectra of peptides corresponding to relevant SAMP1-MoaE conjugate products purified from *H. volcanii* cells co-expressing PylRS2 variants sV1 (DSKN) and $tRNA^{Pyl}2$ in the absence of BocK. (A-B) The incorporation of the natural amino acid at targeted position was indicated by red color. The y-ion (colored blue) and b-ion (colored red) series detected are indicated.

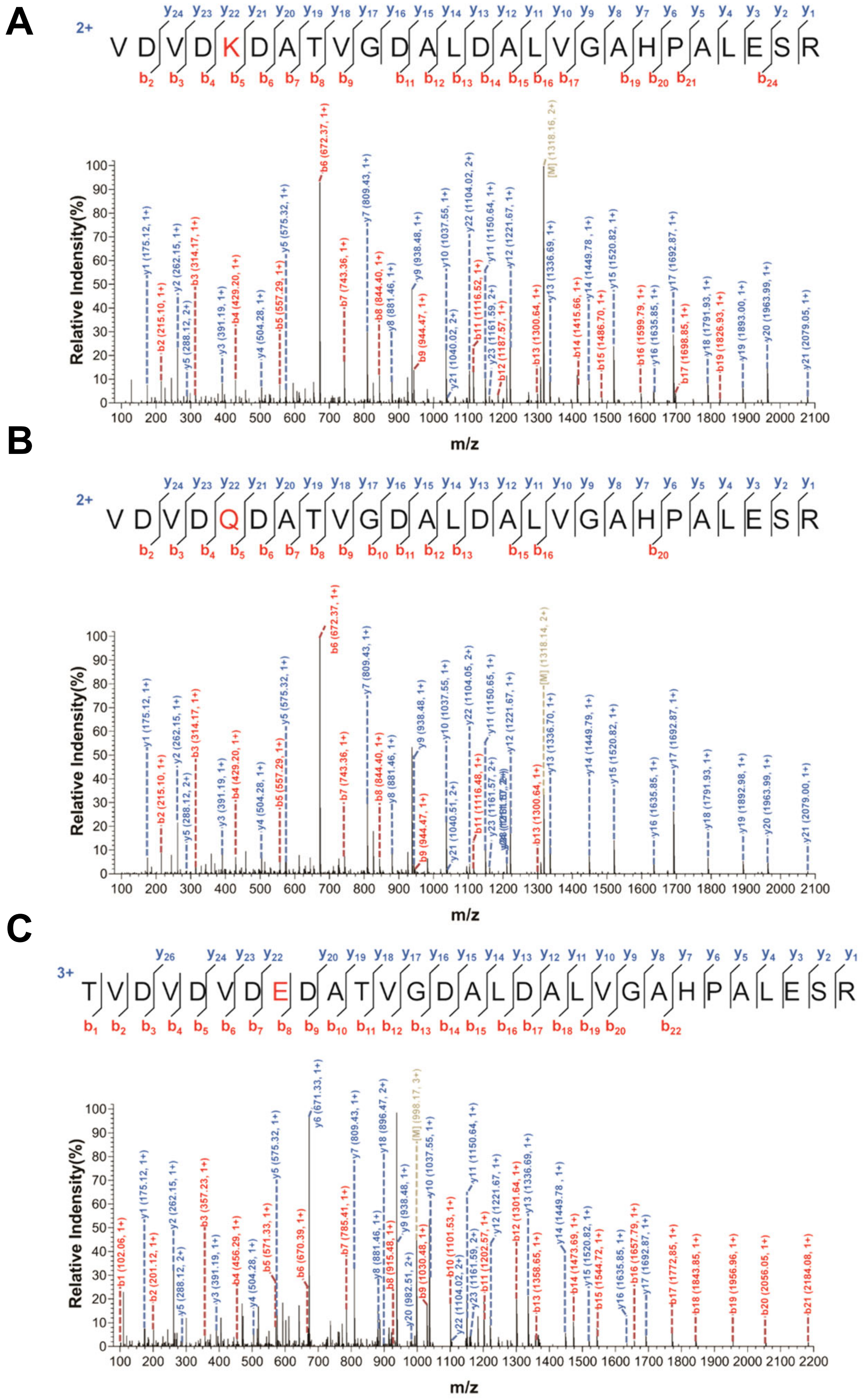


**Fig. S6.** Representative MS/MS spectra of peptides corresponding to relevant SAMP1-MoaE conjugate products purified from *H. volcanii* cells co-expressing PylRS2 variants sV6 (SGKN) and $tRNA^{Pyl}2$ in the absence of BocK. (A-C) The incorporation of the natural amino acid at targeted position was indicated by red color. The y-ion (colored blue) and b-ion (colored red) series detected are indicated.

A

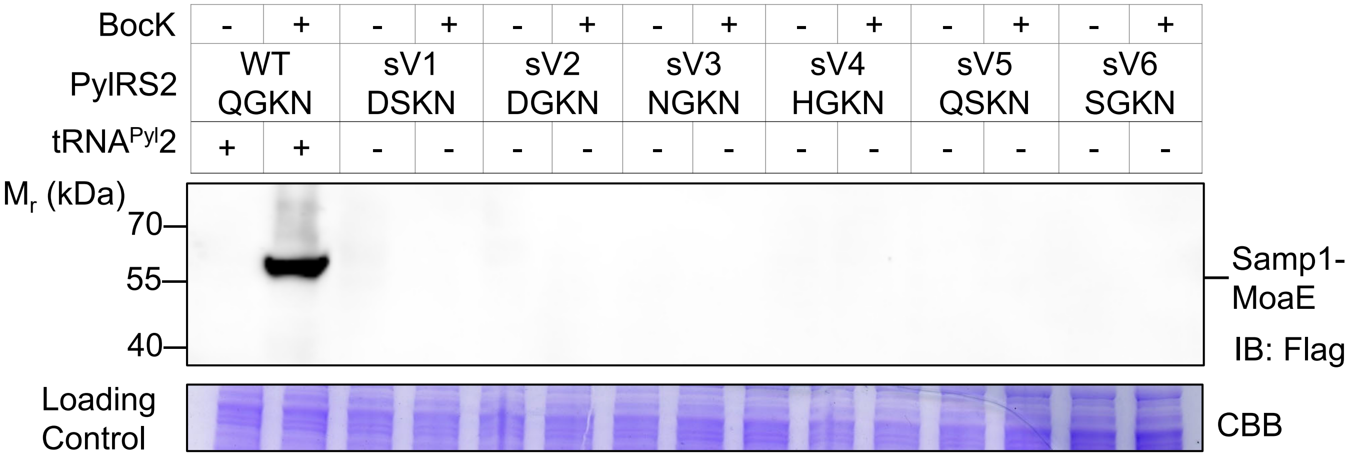


B

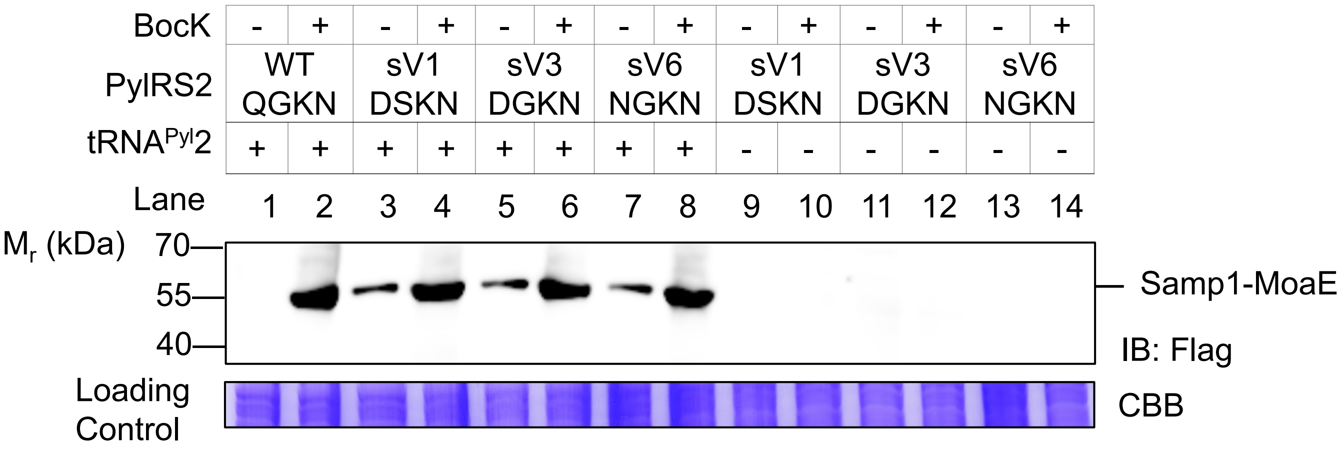


**Fig. S7.** Production of SAMP1-MoaE conjugate products mediated by HMET1 PylRS2/tRNA$^{Pyl}$2-derived pairs. (A) *H. volcanii* cells expressing wild-type HMET1 PylRS2/tRNA$^{Pyl}$2 pair and PylRS2 variants (sV1-sV6). (B) *H. volcanii* cells expressing HMET1 PylRS2 variants (sV1, sV3, sV6) with or without tRNA$^{Pyl}$2. Strains were grown in Hv-YPC medium, supplied with 1mM BocK. Cell lysate was separated by reducing 12% SDS-PAGE and analyzed by anti-Flag immunoblotting (IB) and coomassie blue (CB) staining as indicated on bottom left. Migration of the molecular weight markers (Mr) is indicated on the left. Migration of the SAMP1-MoaE conjugate products is indicated on the right. See Methods for details.

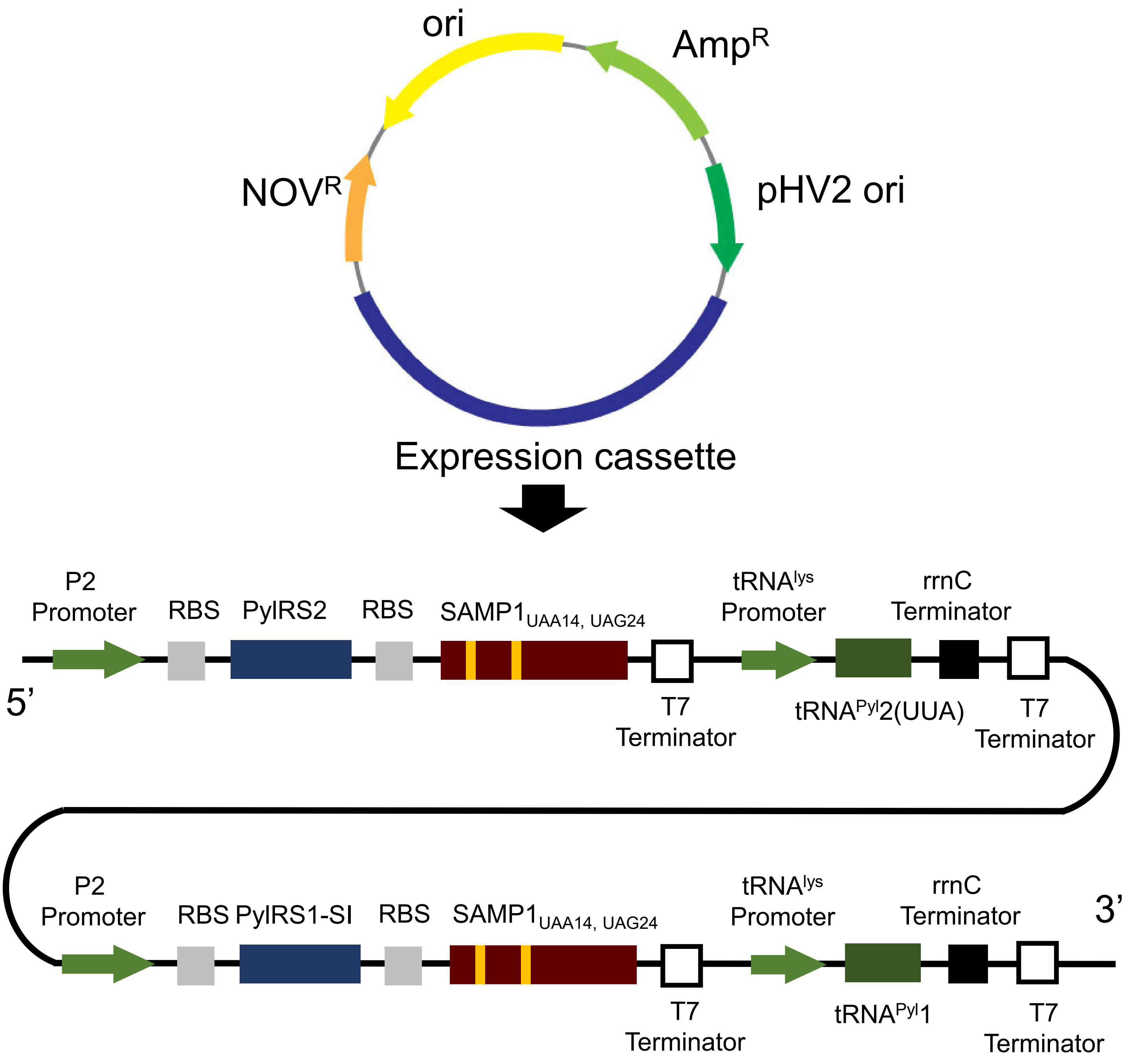


**Fig. S8.** Development of the one-plasmid system for simultaneous incorporation of 3-I-phe and BocK into a single protein in response to UAG and UAA stop codons in *H. volcanii*. Schematic representation of the shuttle plasmid that contains the expression cassette encoding two copies of $SAMP1_{UAA14,\ UAG24}$ and one copy of PylRS1-SI/$tRNA^{Pyl}1$ and PylRS2/$tRNA^{Pyl}2_{UUA}$ pairs. Promoters, terminators and RBS are indicated.

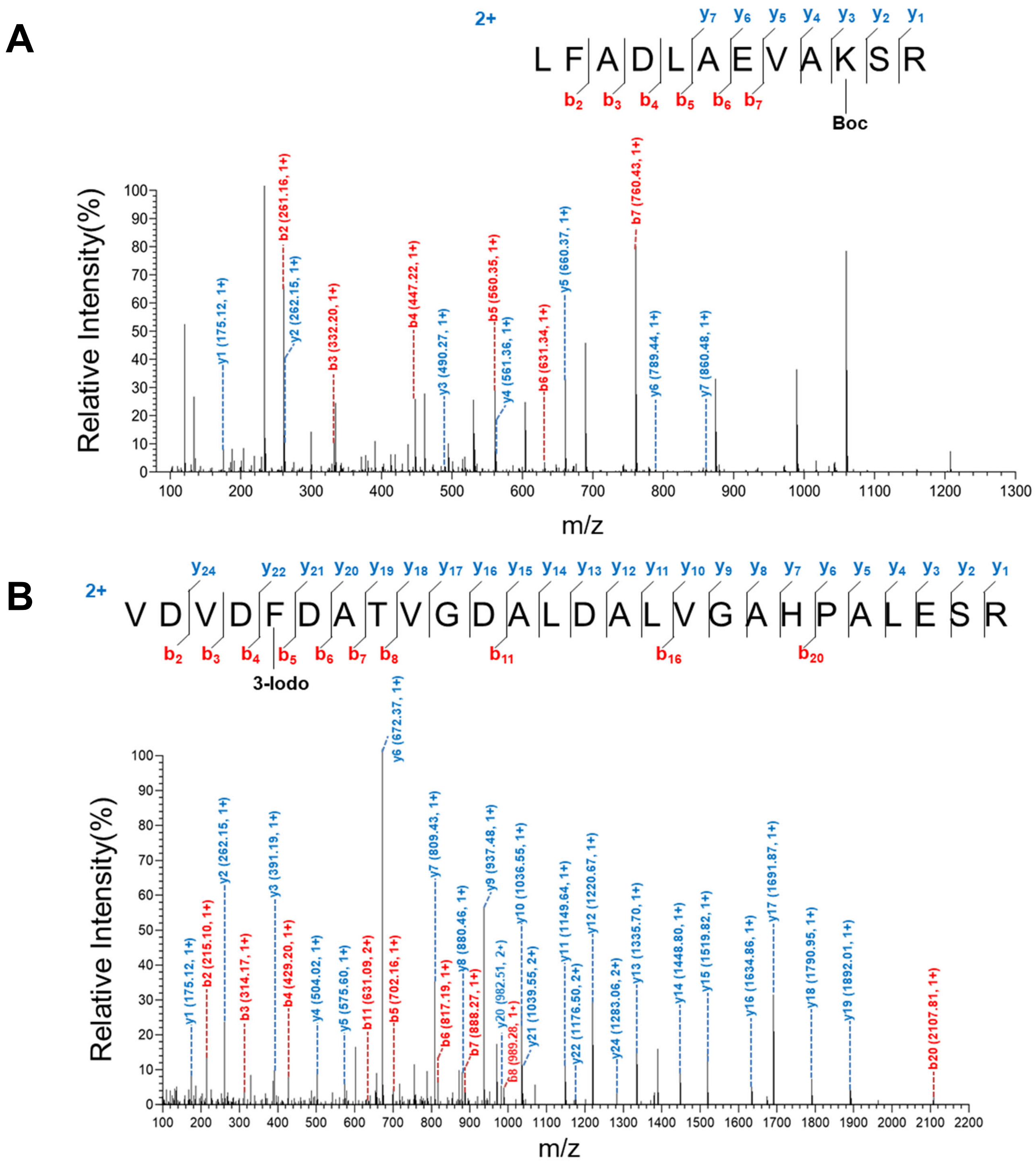


**Fig. S9.** Representative MS/MS spectra of BocK and 3-I-Phe-containing peptides corresponding to SAMP1(3I)14Bock24-MoaE purified from *H. volcanii* cells. 1 liter stationary cells ($OD_{600}$, 2.0 to 3.0) were used for purification of reporter protein via anti-FLAG immunoprecipitation. The y-ion (colored blue) and b-ion (colored red) series detected are indicated.

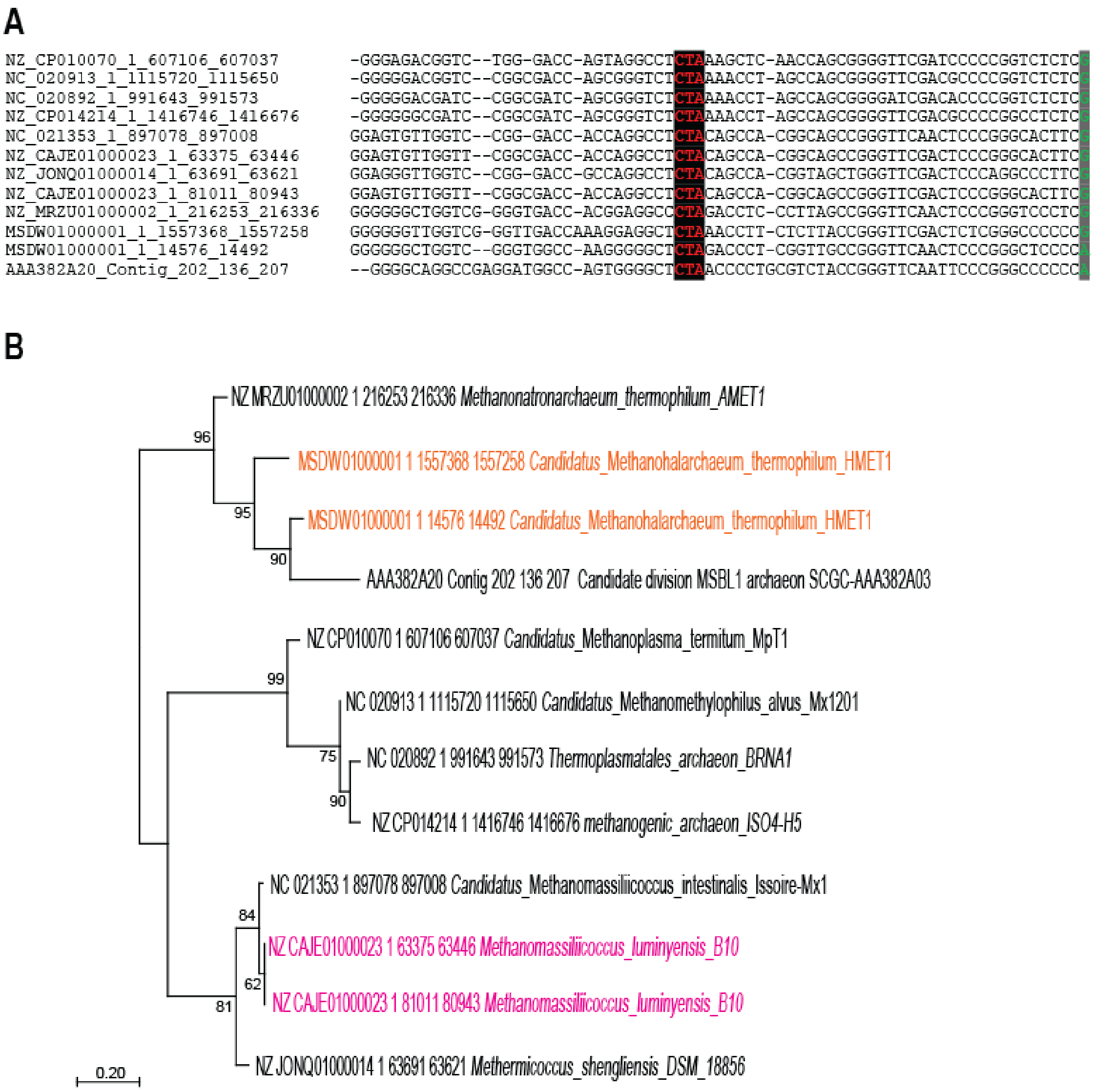


**Fig. S10. Multiple alignment and phylogenetic analysis of tRNAPyl sequences** (A) Multiple alignment of tRNAPyl genes for species encoding ΔPylSn proteins. The sequences are denoted by nucleotide accession and coordinates of the respective genes. The CTA anticodons (red letters, black background) and discriminator bases (green letters, gray background) are indicated. (B) Maximum-likelihood tree was built using IQ-TREE version 2.1.2 based on best-fitting model HKY+F+G4 (35) based on multiple alignment shown on the panel A. The root was placed between Halobacteriota and Thermoplasmatota phyla according to the established archaeal phylogeny (27). The leaves are identified by GenBank nucleotide accession, coordinates of the gene and species names. The sequence for Candidate division MSBL1 archaeon SCGC-AAA382A03 is ref. (61). Tree leaves corresponding to *Candidatus* Methanohalarchaeum thermophilum HMET1 and *Methanomassiliicoccus luminyensis* B10 are highlighted in orange and magenta, respectively.

# Supplementary Tables

**Table S1. List of strains and plasmids used in this study**.

| **Strain, plasmid** | **Description**[a] | **Source or reference** |
|---|---|---|
| **Strains:** | | |
| ***E. coli*** | | |
| DH5α | $F^-$ *80dlacZ M15 (lacZYA-argF) U169 recA1 endA1hsdR17(rk-, mk+) phoAsupE44 -thi-1 gyrA96 relA1* | Clontech Laboratories, Inc. |
| Stellar | $F^-$ *endA1, supE44, thi-1, recA1, relA1, gyrA96, phoA, Φ80d lacZΔ M15, Δ (lacZYA - argF) U169, Δ (mrr - hsdRMS - mcrBC), ΔmcrA, λ–* | Clontech Laboratories, Inc. |
| GM2163 | $F^-$ *ara-14 leuB6 fhuA31 lacY1 tsx78 glnV44 galK2 galT22 mcrA dcm-6 hisG4 rfbD1 rpsL136 dam13*::Tn*9 xylA5 mtl-1 thi-1 mcrB1 hsdR2* | New England Biolabs |
| TOP10 | F- *mcr*A Δ(*mrr-hsd*RMS-*mcr*BC) φ80 *lac*Z*ΔM15* Δ*lac*X74 *rec*A1 *ara*Δ139 Δ(*ara-leu*)7697 *gal*U *gal*K *rps*L ($Str^R$) *end*A1 *nup*G | Shanghai Weidi Biotechnology Co, Ltd |
| ***H. volcanii*** | | |
| H26 | DS70 *ΔpyrE2* | (1) |
| **Plasmids:** | | |
| pJAM202c | $Ap^r$; $Nv^r$; *Hfx. volcanii-E. coli* shuttle plasmid, empty vector | (2) |
| pXF201 | $Ap^r$; $Nv^r$; pJAM202c carries HMET1 *pylRS1* | This study |
| pXF202 | $Ap^r$; $Nv^r$; pJAM202c carries HMET1 *pylRS2* | This study |
| pXF214 | $Ap^r$; $Nv^r$; pJAM202c carries $P2_{rrnA}$-*flag-samp1*, HMET1 *pylRS2* and *$tRNA^{Pyl}$2* | This study |
| pXF216 | $Ap^r$; $Nv^r$; pJAM202c carries $P2_{rrnA}$-*flag-samp1*, HMET1 *pylRS1* and *$tRNA^{Pyl}$1* | This study |
| pXF242 | $Ap^r$; $Nv^r$; pJAM202c carries $P2_{rrnA}$-*RBS-flag-samp1*, HMET1 *pylRS2* and *$tRNA^{Pyl}$2* | This study |
| pXF243 | $Ap^r$; $Nv^r$; pJAM202c carries $P2_{rrnA}$-*RBS-flag-samp1*, HMET1 *pylRS1* and *$tRNA^{Pyl}$1* | This study |
| pXF249 | $Ap^r$; $Nv^r$; pJAM202c carries $P2_{rrnA}$-*RBS-flag-samp1*$_{UAG24}$, HMET1 *pylRS2* and *$tRNA^{Pyl}$2* | This study |
| pXF266 | $Ap^r$; $Nv^r$; pJAM202c carries $P2_{rrnA}$-*RBS-flag-samp1*$_{UAG24}$, HMET1 *pylRS1* and *$tRNA^{Pyl}$1* | This study |
| pXF271 | $Ap^r$; $Nv^r$; pJAM202c carries $P2_{rrnA}$-*RBS-flag-samp1*$_{UAG24}$, HMET1 *pylRS2* and *$tRNA^{Pyl}$2 (A73>G)* | This study |
| pXF274 | $Ap^r$; $Nv^r$; pJAM202c carries $P2_{rrnA}$-*RBS-flag-samp1*$_{UAG24}$, HMET1 *pylRS1* and *$tRNA^{Pyl}$2* | This study |
| pXF275 | $Ap^r$; $Nv^r$; pJAM202c carries $P2_{rrnA}$-*RBS-flag-samp1*$_{UAG24}$, HMET1 *pylRS2* and *$tRNA^{Pyl}$1* | This study |
| pXF278 | $Ap^r$; $Nv^r$; pJAM202c carries $P2_{rrnA}$-*RBS-flag-samp1*$_{UAG24}$ and HMET1 *pylRS1* | This study |
| pXF279 | $Ap^r$; $Nv^r$; pJAM202c carries $P2_{rrnA}$-*RBS-flag-samp1*$_{UAG24}$ and HMET1 *pylRS2* | This study |
| pXF282 | $Ap^r$; $Nv^r$; pJAM202c carries $P2_{rrnA}$-*RBS-flag-samp1*$_{UAG24}$ and HMET1 *$tRNA^{Pyl}$1* | This study |
| pXF283 | $Ap^r$; $Nv^r$; pJAM202c carries $P2_{rrnA}$-*RBS-flag-samp1*$_{UAG24}$ and HMET1 *$tRNA^{Pyl}$2* | This study |
| pXF310 | $Ap^r$; $Nv^r$; pJAM202c carries $P2_{rrnA}$-*RBS-flag-samp1*$_{UAG24}$, HMET1 *pylRS2* and *$mtRNA^{Pyl}$1* | This study |
| pXF311 | $Ap^r$; $Nv^r$; pJAM202c carries $P2_{rrnA}$-*RBS-flag-samp1*$_{UAG24}$, HMET1 *mPylRS1* and *$tRNA^{Pyl}$2* | This study |
| pXF319 | $Ap^r$; $Nv^r$; pJAM202c carries $P2_{rrnA}$-*RBS-flag-samp1*$_{UAG24}$, HMET1 *pylRS1* and *$mtRNA^{Pyl}$1* | This study |
| pXF333 | $Ap^r$; $Nv^r$; pJAM202c carries $P2_{rrnA}$-*RBS-flag-samp1*$_{UAG24}$, HMET1 *pylRS2*(sV1) and *$tRNA^{Pyl}$2* | This study |

| | | |
|---|---|---|
| pXF334 | Ap$^r$; Nv$^r$; pJAM202c carries P2$_{rrnA}$-*RBS-flag-samp1*$_{UAG24}$, HMET1 *pylRS2* (sV2) and *tRNA*$^{Pyl}$*2* | This study |
| pXF335 | Ap$^r$; Nv$^r$; pJAM202c carries P2$_{rrnA}$-*RBS-flag-samp1*$_{UAG24}$, HMET1 *pylRS2* (sV3) and *tRNA*$^{Pyl}$*2* | This study |
| pXF336 | Ap$^r$; Nv$^r$; pJAM202c carries P2$_{rrnA}$-*RBS-flag-samp1*$_{UAG24}$, HMET1 *pylRS2* (sV4) and *tRNA*$^{Pyl}$*2* | This study |
| pXF337 | Ap$^r$; Nv$^r$; pJAM202c carries P2$_{rrnA}$-*RBS-flag-samp1*$_{UAG24}$, HMET1 *pylRS2* (sV5) and *tRNA*$^{Pyl}$*2* | This study |
| pXF338 | Ap$^r$; Nv$^r$; pJAM202c carries P2$_{rrnA}$-*RBS-flag-samp1*$_{UAG24}$, HMET1 *pylRS2* (sV6) and *tRNA*$^{Pyl}$*2* | This study |
| pXF339 | Ap$^r$; Nv$^r$; pJAM202c carries P2$_{rrnA}$-*RBS-flag-samp1*$_{UAG24}$, HMET1 *pylRS2* (cV1) and *tRNA*$^{Pyl}$*2* | This study |
| pXF340 | Ap$^r$; Nv$^r$; pJAM202c carries P2$_{rrnA}$-*RBS-flag-samp1*$_{UAG24}$, HMET1 *pylRS2* (cV2) and *tRNA*$^{Pyl}$*2* | This study |
| pXF341 | Ap$^r$; Nv$^r$; pJAM202c carries P2$_{rrnA}$-*RBS-flag-samp1*$_{UAG24}$, HMET1 *pylRS2* (cV3) and *tRNA*$^{Pyl}$*2* | This study |
| pXF342 | Ap$^r$; Nv$^r$; pJAM202c carries P2$_{rrnA}$-*RBS-flag-samp1*$_{UAG24}$, HMET1 *pylRS2* (cV4) and *tRNA*$^{Pyl}$*2* | This study |
| pXF343 | Ap$^r$; Nv$^r$; pJAM202c carries P2$_{rrnA}$-*RBS-flag-samp1*$_{UAG24}$, HMET1 *pylRS2* (cV5) and *tRNA*$^{Pyl}$*2* | This study |
| pXF344 | Ap$^r$; Nv$^r$; pJAM202c carries P2$_{rrnA}$-*RBS-flag-samp1*$_{UAG24}$, HMET1 *pylRS2* (cV6) and *tRNA*$^{Pyl}$*2* | This study |
| pXF347 | Sm$^r$; pBAD carries P$_{ara}$-*sfGFP (N150amb)* | This study |
| pXF348 | Sm$^r$; pBAD carries P$_{ara}$-*sfGFP (WT)* | This study |
| pXF349 | Ap$^r$; pSCW11 carries P$_{lpp}$-*pylRS*-P$_{proK}$-*pylT*, *M. alvus pylRS* and *tRNA*$^{Pyl}$ | This study |
| pXF350 | Ap$^r$; pSCW11 carries P$_{lpp}$-*PylRS*-P$_{proK}$-*pylT*, HMET1 *pylRS2* and *tRNA*$^{Pyl}$*2* | This study |
| pXF358 | Km$^r$; pET28(a) carries P$_{T7}$-HMET1 *pylRS2* | This study |
| pXF359 | Km$^r$; pET28(a) carries P$_{T7}$-HMET1 *pylRS*1 | This study |
| pXF365 | Ap$^r$; Nv$^r$; pJAM202c carries P2$_{rrnA}$-*RBS-flag-samp1*$_{UAG24}$, HMET1 *mPylRS1* and *tRNA*$^{Pyl}$*1* | This study |
| pXF366 | Ap$^r$; Nv$^r$; pJAM202c carries P2$_{rrnA}$-*RBS-flag-samp1*$_{UAG24}$, HMET1 *pylRS2* (sV1) | This study |
| pXF367 | Ap$^r$; Nv$^r$; pJAM202c carries P2$_{rrnA}$-*RBS-flag-samp1*$_{UAG24}$, HMET1 *pylRS2* (sV2) | This study |
| pXF368 | Ap$^r$; Nv$^r$; pJAM202c carries P2$_{rrnA}$-*RBS-flag-samp1*$_{UAG24}$, HMET1 *pylRS2* (sV3) | This study |
| pXF369 | Ap$^r$; Nv$^r$; pJAM202c carries P2$_{rrnA}$-*RBS-flag-samp1*$_{UAG24}$, HMET1 *pylRS2* (sV4) | This study |
| pXF370 | Ap$^r$; Nv$^r$; pJAM202c carries P2$_{rrnA}$-*RBS-flag-samp1*$_{UAG24}$, HMET1 PylRS2 (sV5) | This study |
| pXF371 | Ap$^r$; Nv$^r$; pJAM202c carries P2$_{rrnA}$-*RBS-flag-samp1*$_{UAG24}$, HMET1 *pylRS2* (sV6) | This study |
| pXF411 | Ap$^r$; Nv$^r$; pJAM202c carries P2$_{rrnA}$-*RBS-flag-samp1*$_{UAA14}$, HMET1 *pylRS*2 and *tRNA*$^{Pyl}$*2*$_{UUA}$ | This study |
| pXF376 | Ap$^r$; Nv$^r$; pJAM202c carries P2$_{rrnA}$-*RBS-flag-samp1*$_{UAG24}$, HMET1 *pylRS1-SI* and *tRNA*$^{Pyl}$1 | This study |
| pXF379 | Ap$^r$; pSCW11 carries P$_{lpp}$-*pylRS*-P$_{proK}$-*pylT*, *M. mazei pylRS* and *tRNA*$^{Pyl}$ | This study |
| pXF382 | Km$^r$; pET28(a) carries P$_{T7}$-*M. alvus pylRS* | This study |
| pXF398 | Ap$^r$; Nv$^r$; pJAM202c carries P2$_{rrnA}$-*RBS-flag-samp1*$_{UAA14,\ UAG24}$, HMET1 *pylRS1-SI*, *tRNA*$^{Pyl}$1, *pylRS2* and *tRNA*$^{Pyl}$*2*$_{UUA}$ | This study |

[a]Ap$^r$, ampicillin resistance; Nv$^r$, novobiocin resistance; Km$^r$, kanamycin resistance. Sm$^r$, Spectinomycin resistance. SAMP1, HVO_2619;

**Table S2. List of primers used in this study**

| Primer Pair | Primer Sequence (5'-3')[a] | Description; construct generated |
|---|---|---|
| HMET1 PylRS FW | 5'-ACGAACTCGCGCTGTTCCCG -3' | pXF242/243 backbone amplification primers; pXF249 and pXF266 |
| HMET1 PylRS RV | 5'-CCTTTCGGGCTTTGTTAGCAGC -3' | |
| SAMP FW | 5'-GCTGCTAACAAAGCCCGAAAGG -3' | Primers that amplificate the Flag-SAMP1 sequences contain site mutations; pXF249 and pXF266 |
| SAMP RV | 5'-CGGGAACAGCGCGAGTTCGT -3' | |
| loop Pyl2to1 FW | 5'-**AAAGGCGGCAAGGACCACCTCAA**cgagttcaagatgctcaacgccgtc -3' | Primers change the motif 2 loop from RKEQGKNHVR(6aa) to RKEKGGKDHLN(7aa); pXF270 |
| loop Pyl2to1 RV | 5'-**GTTGAGGTGGTCCTTGCCGCCTTT**ctccttgcggaagcaggtcc -3' | |
| loop Pyl1to2 FW | 5'-**GAGCAGGGGAAGAACCACGTCCG**cgagttcaagatgctcaacgccgtc -3' | Primers change the motif 2 loop from RKEKGGKDHLN(7aa) to RKEQGKNHVR(6aa); pXF311 |
| loop Pyl1to2 RV | 5'-**CTCGCGGACGTGGTTCTTCCCCTG**ctccttgcggaagcaggtcccg -3' | |
| 73A>G FW | 5'-ctgactatggaattaatccttagCGAAAG -3' | Point mutate 73A of tRNA2 to G; pXF271 |
| 73A>G RV | 5'-ctaaggattaattccatagtcag**C**GGGGAGCCC -3' | |
| PylRS Remove FW | 5'-tcgacgaactctgaacctatgaatcgcggctgctaacaaagcccgaaagg -3' | Remove PylRS1 from pXF249 and PylRS2 from pXF266; pXF282 and pXF283 |
| PylRS Remove RV | 5'-cctttcgggctttgttagcagccgcgattcataggttcagagttcgtcga -3' | |
| tRNA Remove FW | 5'-gccggatatagttcctcctttcagccacccttccatctcgtgcc -3' | Remove tRNA1 from pXF249 and tRNA2 from pXF266; pXF278 and pXF279 |
| tRNA Remove RV | 5'-ggcacgagatggaagggtggctgaaaggaggaactatatccggc -3' | |
| 73G>A FW | 5'-gttcgactctcgggccccccaTTCCCAAATCCAAAAATCCTT**A** -3' | Point mutate 73G of tRNA1 to A; pXF310 |
| 73G>A RV | 5'-ggggcccgagagtcgaacCCGGTAAGAGAAGG -3' | |
| Sv1 FW | 5'-tcgggacctgcttccgcaaggag**GACAGCAAGAAC**cacgtccgcgagttcaagatgctcaa -3' | Primers change the motif 2 loop from RKEQGKNHVR to RKEDSKNHVR; pXF333 |
| Sv1 RV | 5'-ttgagcatcttgaactcgcggacgtg**GTTCTTGCTGTC**ctccttgcggaagcaggtcccga -3' | |
| Sv2 FW | 5'-ctcgggacctgcttccgcaaggag**GACGGCAAGAAC**cacgtccgcgagttcaagatgctcaa -3' | Primers change the motif 2 loop from RKEQGKNHVR to RKEDGKNHVR; pXF334 |
| Sv2 RV | 5'-ttgagcatcttgaactcgcggacgtg**GTTCTTGCCGTC**ctccttgcggaagcaggtcccgag -3' | |
| Sv3 FW | 5'-tcgggacctgcttccgcaaggaga**ACGGCAAGAAC**cacgtccgcgagttcaagatgctcaa -3' | Primers change the motif 2 loop from RKEQGKNHVR to RKENGKNHVR; pXF335 |
| Sc3 RV | 5'-ttgagcatcttgaactcgcggacgtg**GTTCTTGCCGTT**ctccttgcggaagcaggtcccga -3' | |
| Sv4 FW | 5'-tcgggacctgcttccgcaaggag**CACGGCAAGAAC**cacgtccgcgagttcaagatgctcaa -3' | Primers change the motif 2 loop from RKEQGKNHVR to RKEHGKNHVR; pXF336 |

| | | |
|---|---|---|
| Sv4 RV | 5'-ttgagcatcttgaactcgcggacgtg**GTTCTTGCCGTG**ctccttgcggaagcaggtcccga -3’ | |
| Sv5 FW | 5'-tcgggacctgcttccgcaaggag**CAGAGCAAGAAC**cacgtccgcgagttcaagatgctcaa -3’ | Primers change the motif 2 loop from RKEQGKNHVR to RKEQSKNHVR; pXF337 |
| Sv5 RV | 5'-ttgagcatcttgaactcgcggacgtg**GTTCTTGCTCTG**ctccttgcggaagcaggtcccga -3’ | |
| Sv6 FW | 5'-tcgggacctgcttccgcaaggaga**GCGGCAAGAAC**cacgtccgcgagttcaagatgctcaa -3’ | Primers change the motif 2 loop from RKEQGKNHVR to RKESGKNHVR; pXF338 |
| Sv6 RV | 5'-ttgagcatcttgaactcgcggacgtg**GTTCTTGCCGCT**ctccttgcggaagcaggtcccga -3’ | |
| Cv1 FW | 5'-tcgggacctgcttccgcaaggaga**GCGACGGCAAGGAG**cacctcaacgagttcaagatgctcaa -3’ | Primers change the motif 2 loop from RKEQGKNHVR to RKESDKGEHVR; pXF339 |
| Cv1 RV | 5'-ttgagcatcttgaactcgttgaggtg**CTCCTTGCCGTCG**ctctccttgcggaagcaggtcccga -3’ | |
| Cv2 FW | 5'-tcgggacctgcttccgcaaggagag**CCAGGGCGCCCAG**cacctcaacgagttcaagatgctcaa -3’ | Primers change the motif 2 loop from RKEQGKNHVR to RKESQGAQHVR; pXF340 |
| Cv2 RV | 5'-ttgagcatcttgaactcgttgaggtg**CTGGGCGCCCTGG**ctctccttgcggaagcaggtcccga -3’ | |
| Cv3 FW | 5'-tcgggacctgcttccgcaaggaga**GCCACAGCGGCATG**cacctcaacgagttcaagatgctcaa -3’ | Primers change the motif 2 loop from RKEQGKNHVR to RKESHSGMHVR pXF341 |
| Cv3 RV | 5'-tcgggacctgcttccgcaaggaga**CATGCCGCTGTGG**ctctccttgcggaagcaggtcccga -3’ | |
| Cv4 FW | 5'-tcgggacctgcttccgcaaggaga**GCCACAGCGGCAGC**cacctcaacgagttcaagatgctcaa -3’ | Primers change the motif 2 loop from RKEQGKNHVR to RKESHSGSHVR; pXF342 |
| Cv4 RV | 5'-tcgggacctgcttccgcaaggaga**GCTGCCGCTGTGG**ctctccttgcggaagcaggtcccga -3’ | |
| Cv5 FW | 5'-tcgggacctgcttccgcaaggaga**GCAAGGGCAGCAAC**cacctcaacgagttcaagatgctcaa -3’ | Primers change the motif 2 loop from RKEQGKNHVR to RKESKGSNHVR; pXF343 |
| Cv5 RV | 5'-tcgggacctgcttccgcaaggaga**GTTGCTGCCCTTG**ctctccttgcggaagcaggtcccga -3’ | |
| Cv6 FW | 5'-tcgggacctgcttccgcaaggaga**CGCACGGCAGCGACcacctcaacgagttcaagatgctcaa** -3’ | Primers change the motif 2 loop from RKEQGKNHVR to RKETHGSDHVR; pXF344 |
| Cv6 RV | 5'-tcgggacctgcttccgcaaggaga**GTCGCTGCCGTGCGT**ctccttgcggaagcaggtcccga -3’ | |
| UAA FW | 5'- gccaagggggct**tta**gaccctcg -3’ | Primers change the anticodon of tRNA2 from UAG to UAA |
| UAA RV | 5'- cgagggtc**taa**agcccccttggc -3’ | |
| LSAIE FW | 5'-aacgagttcaagatgctg**agc**gcc**att**gaactgggcaacttcaaggacaa -3’ | Primers engineer the active site of PylRS2 to charge 3-I-Phe onto tRNA2; pXF376 |
| LSAIE RV | 5'-ttgtccttgaagttgcccagttc**aat**ggc**gct**cagcatcttgaactcgttgaggtggtccTTGCCGCCTTT -3’ | |
| G14 FW | 5'-TGTTCGCCGACCTCGCGGAAGTCGCC**TAA**TCGCGCACCGTTCGGGTCGACGTCGA -3’ | Point mutate G14 of Samp1 to TAA |

| | | |
|---|---|---|
| G14 RV | 5'-TCGACGTCGACCCGAACGGTGCGCG**ATT**AGGCGACTTCCGCGAGGTCGGCGAACA -3’ | |
| Cassette insert FW | 5'-CTGAAAGGAGGAACTATATCCGGCCGTCGACGAACTCTGAACCTATGAAT -3’ | Primers amplificate the cassette; pXF398 |
| Cassette insert RV | 5'-GGTGAGTACTTAACGCGGGACTTCAACGCCCCGAGTTCACTTTCACTCC -3’ | |
| Cassette insert FW | 5'-GGAGTGAAAGTGAACTCGGGGCGTTGAAGTCCCGCGTTAAGTACTCACC -3’ | Primers amplificate the backbone that excludes the cassette; pXF398 |
| Cassette insert RV | 5'-ATTCATAGGTTCAGAGTTCGTCGACGGCCGGATATAGTTCCTCCTTTCAG -3’ | |
| pBAD FW<br>pBAD RV | 5’-TAAAGCTCGAGATCTGCAGC-3’<br>5’-CATGGTTAATTCCTCCTGTTAG-3’ | pBAD-pCDF backbone amplification primers; pXF347 and pXF348 |
| pSCW11 FW<br>pSCW11 RV | 5’-GGTTCAGGTGGCACTTTTCG-3’<br>5’-CATGGTTAATTCCTCCTGTTAG-3’ | pSCW11 backbone amplification primers; pXF349, pXF350 and pXF379 |
| sfGFP WT FW<br>sfGFP WT RV | 5’-CTAACAGGAGGAATTAACCATGGTTAGCAAAGGTGAAGAAC-3’<br>5’-CAGCTGCAGATCTCGAGCTTTAATGGTGATGATGATGG-3 | Primers that amplificate the wild type sfGFP sequences; pXF347 |
| sfGFP TAA FW<br>sfGFP TAA RV | 5’-GCCATTAGGTGTATATTACCGCCGATAAACAG-3’<br>5’-CGGTAATATACACCTAATGGCTGTTGAAATTATATTCCAG-3 | Primers mutate the N150 of sfGFP into TAA; pXF348 |
| PylRS-tRNA FW<br>PylRS-tRNA RV | 5’-CCCGAAAAGTGCCACCTGAACCCAGCGTTCGATGCTTC-3’<br>5’-CTAGAACTAGTGGATCCCCCCTAACAATGCGCTCATCGTC-3 | Primers that amplificate the $P_{lpp}$-PylRS-$P_{proK}$-tRNA sequences; pXF349, pXF350 and pXF379 |
| PylRS1 FW<br>PylRS1 RW | 5’-GTGCCGCGCGGCAGCCATATGGAATTTACCGAAACAC-3’<br>5’-CGAGTGCGGCCGCAAGCTTTTATTTGATGTCCAGACG-3 | Primers that amplificate the HMET11 PylRS1 sequences; pXF359 |
| PylRS2 FW<br>PylRS2 RW | 5’-GTGCCGCGCGGCAGCCATATGGAACTGACCCGTAGC-3’<br>5’-CGAGTGCGGCCGCAAGCTTTTAGTTAATTTCCAGGCG-3 | Primers that amplificate the HMET11 PylRS2 sequences; pXF358 |
| PylRS FW<br>PylRS RW | 5’-GTGCCGCGCGGCAGCCATATGACCGTGAAATATACCG-3’<br>5’-GAGTGCGGCCGCAAGCTTTTAATTGATTTTGGCACC-3 | Primers that amplificate the *M.alvus* PylRS2 sequences; pXF382 |

[a]Oligonucleotide sequences introduced in primers to facilitate cloning are in lowercase, sites for site-directed mutagenesis are in bold.

**Table S3. PylRS enzymes in arCOG database.**

| Protein accession | PylRS classification | Species |
|---|---|---|
| KUK29526_1 | ΔPylSn | Methanosarcinales_archeaon_56_1174_ |
| WP_019178529_1 | ΔPylSn | Methanomassiliicoccus_luminyensis_B10 |
| OKY77552_1 | ΔPylSn | Candidatus_Methanohalarchaeum_thermophilum_ |
| OKY79096_1 | ΔPylSn | Candidatus_Methanohalarchaeum_thermophilum_ |
| WP_015492598_1 | ΔPylSn | Thermoplasmatales_archaeon_BRNA1 |
| WP_048111907_1 | ΔPylSn | Candidatus_Methanoplasma_termitum_MpT1 |
| WP_066075773_1 | ΔPylSn | methanogenic_archaeon_ISO4-H5 |
| WP_019176308_1 | ΔPylSn | Methanomassiliicoccus_luminyensis_B10 |
| WP_020448777_1 | ΔPylSn | Candidatus_Methanomassiliicoccus_intestinalis_Issoire-Mx1 |
| WP_015505008_1 | ΔPylSn | Candidatus_Methanomethylophilus_alvus_Mx1201 |
| KUK04055_1 | ΔPylSn | Euryarchaeota_archaeon_55_53_ |
| CDF30953_1 | ΔPylSn | Methanoculleus_sp_CAG_1088_ |
| WP_042686913_1 | ΔPylSn | Methermicoccus_shengliensis_DSM_18856 |
| WP_086636640_1 | ΔPylSn | Methanonatronarchaeum_thermophilum_AMET1 |
| OBZ34501_1 | PylS | Methanohalophilus_sp_DAL1_ |
| WP_096712291_1 | PylS | Methanohalophilus_euhalobius_DSM_10369 |
| WP_013036758_1 | PylS | Methanohalophilus_mahii_DSM_5219 |
| ODV50257_1 | PylS | Methanohalophilus_sp_2-GBenrich_ |
| OBZ34613_1 | PylS | Methanohalophilus_sp_DAL1_ |
| WP_072560754_1 | PylS | Methanohalophilus_halophilus_Z-7982 |
| WP_072358806_1 | PylS | Methanohalophilus_portucalensis_FDF-1T |
| WP_048204558_1 | PylS | Methanococcoides_methylutens_MM1 |
| WP_013193804_1 | PylS | Methanohalobium_evestigatum_Z-7303 |
| WP_011500100_1 | PylS | Methanococcoides_burtonii_DSM_6242 |
| WP_054298905_1 | PylS | Methanosarcina_flavescens_E03_2 |
| WP_011305865_1 | PylS | Methanosarcina_barkeri_str_Wiesmoor |
| ALK04970_1 | PylS | Methanosarcina_sp_795_ |
| WP_048157962_1 | PylS | Methanosarcina_sp_Kolksee |
| WP_048167695_1 | PylS | Methanosarcina_thermophila_TM-1 |
| WP_048123171_1 | PylS | Methanosarcina_vacuolata_Z-761 |
| WP_048142694_1 | PylS | Methanosarcina_horonobensis_HB-1_JCM_15518 |
| WP_013898320_1 | PylS | Methanosalsum_zhilinae_DSM_4017 |
| WP_015323491_1 | PylS | Methanomethylovorans_hollandica_DSM_15978 |
| WP_015053640_1 | PylS | Methanolobus_psychrophilus_R15 |
| WP_011020213_1 | PylS | Methanosarcina_acetivorans_C2A |
| WP_048178985_1 | PylS | Methanosarcina_siciliae_C2J |
| WP_023845214_1 | PylS | Methanolobus_tindarius_DSM_2278 |
| WP_011033391_1 | PylS | Methanosarcina_mazei_Go1 |
| WP_048050733_1 | PylS | Methanosarcina_soligelidi_SMA-21 |
| WP_048130001_1 | PylS | Methanosarcina_sp_WH1 |
| WP_048130001_1 | PylS | Methanosarcina_sp_WWM596 |
| WP_048181983_1 | PylS | Methanosarcina_sp_MTP4 |

| | | |
|---|---|---|
| WP_048136948_1 | PylS | Methanosarcina_sp_2_H_T_1A_3 |
| WP_048160059_1 | PylS | Methanosarcina_sp_2_H_T_1A_6 |
| WP_048136948_1 | PylS | Methanosarcina_sp_2_H_T_1A_8 |
| WP_048128743_1 | PylS | Methanosarcina_lacustris_Z-7289 |
| WP_048169762_1 | PylS | Methanosarcina_sp_2_H_A_1B_4 |

**Note:** The shaded sequences were not included in the phylogenetic analysis (Fig. 1, Additional File 1) because they are identical to the proteins from the same set but encoded in the genomes of a better quality.

**Table S4.** Detailed information on genomic neighborhood of *pylS* genes based on the data from arCOG database.

| Protein accession | Gene name | Description | Species |
|---|---|---|---|
| WP_080503037_1 | - | Uncharacterized protein | Methanosarcina_mazei_Go1 |
| WP_080503038_1 | - | Transposase, IS5 family | Methanosarcina_mazei_Go1 |
| WP_080503039_1 | #N/A | #N/A | Methanosarcina_mazei_Go1 |
| WP_011033379_1 | AcrB | Cation/multidrug efflux pump | Methanosarcina_mazei_Go1 |
| WP_011033380_1 | - | Uncharacterized membrane protein | Methanosarcina_mazei_Go1 |
| WP_011033381_1 | PotE | Amino acid transporter | Methanosarcina_mazei_Go1 |
| WP_048041216_1 | MtbC1 | Methanogenic corrinoid protein MtbC1 | Methanosarcina_mazei_Go1 |
| WP_011033385_1 | MtbA | Methylcobalamin:coenzyme M methyltransferase | Methanosarcina_mazei_Go1 |
| WP_011033386_1 | - | Uncharacterized Fe-S clusters-containing protein, contains DUF4445 domain | Methanosarcina_mazei_Go1 |
| WP_011033387_1 | Ppa | Inorganic pyrophosphatase | Methanosarcina_mazei_Go1 |
| WP_011033388_1 | PylD | (3R)-3-methyl-D-ornithyl-N6-L-lysine dehydrogenase, pyrrolysine biosynthesis enzyme | Methanosarcina_mazei_Go1 |
| WP_011033389_1 | PylC | (3R)-3-methyl-D-ornithine:L-lysine ligase, pyrrolysine biosynthesis protein | Methanosarcina_mazei_Go1 |
| WP_011033390_1 | PylB/HcgA | (2R,3R)-3-methylornithine synthase involved in pyrrolysine biosynthesis/Iron-guanylylpyridinol (FeGP) cofactor biosynthesis protein HcgA | Methanosarcina_mazei_Go1 |
| WP_011033391_1 | PylS | Pyrrolysyl-tRNA-synthetase | Methanosarcina_mazei_Go1 |
| WP_011033392_1 | #N/A | #N/A | Methanosarcina_mazei_Go1 |
| WP_011033393_1 | - | Class II terpene cyclase family protein | Methanosarcina_mazei_Go1 |
| WP_048046011_1 | - | Predicted DNA-binding protein | Methanosarcina_mazei_Go1 |
| WP_011033395_1 | - | Uncharacterized protein | Methanosarcina_mazei_Go1 |
| WP_011033396_1 | - | Homolog of Wybutosine (yW) biosynthesis enzyme, Fe-S oxidoreductase | Methanosarcina_mazei_Go1 |
| WP_011033397_1 | UspA | Nucleotide-binding protein, UspA family | Methanosarcina_mazei_Go1 |
| WP_011033398_1 | UspA | Nucleotide-binding protein, UspA family | Methanosarcina_mazei_Go1 |
| WP_011033399_1 | UspA | Nucleotide-binding protein, UspA family | Methanosarcina_mazei_Go1 |
| WP_011033400_1 | UspA | Nucleotide-binding protein, UspA family | Methanosarcina_mazei_Go1 |
| WP_011033401_1 | UspA | Nucleotide-binding protein, UspA family | Methanosarcina_mazei_Go1 |
| | | | Methanosarcina_acetivorans_C2A |
| WP_011020203_1 | MtbC1 | Methanogenic corrinoid protein MtbC1 | Methanosarcina_acetivorans_C2A |
| WP_048064825_1 | #N/A | #N/A | Methanosarcina_acetivorans_C2A |
| WP_011020204_1 | MtbA | Methylcobalamin:coenzyme M methyltransferase | Methanosarcina_acetivorans_C2A |
| WP_011020205_1 | MdaB | NADPH-quinone reductase (modulator of drug activity B) | Methanosarcina_acetivorans_C2A |
| WP_011020206_1 | - | SAM-dependent methyltransferase | Methanosarcina_acetivorans_C2A |
| WP_011020207_1 | - | Transposase | Methanosarcina_acetivorans_C2A |

| | | | |
|---|---|---|---|
| WP_011020208_1 | - | Uncharacterized Fe-S clusters-containing protein, contains DUF4445 domain | Methanosarcina_acetivorans_C2A |
| WP_083755863_1 | WecB | UDP-N-acetylglucosamine 2-epimerase | Methanosarcina_acetivorans_C2A |
| WP_052279090_1 | WecB | UDP-N-acetylglucosamine 2-epimerase | Methanosarcina_acetivorans_C2A |
| WP_011020209_1 | CDA1 | Peptidoglycan/xylan/chitin deacetylase, PgdA/CDA1 family | Methanosarcina_acetivorans_C2A |
| WP_011020210_1 | PylD | (3R)-3-methyl-D-ornithyl-N6-L-lysine dehydrogenase, pyrrolysine biosynthesis enzyme | Methanosarcina_acetivorans_C2A |
| WP_011020211_1 | PylC | (3R)-3-methyl-D-ornithine:L-lysine ligase, pyrrolysine biosynthesis protein | Methanosarcina_acetivorans_C2A |
| WP_011020212_1 | PylB/HcgA | (2R,3R)-3-methylornithine synthase involved in pyrrolysine biosynthesis/Iron-guanylylpyridinol (FeGP) cofactor biosynthesis protein HcgA | Methanosarcina_acetivorans_C2A |
| WP_011020213_1 | PylS | Pyrrolysyl-tRNA-synthetase | Methanosarcina_acetivorans_C2A |
| WP_048064826_1 | #N/A | #N/A | Methanosarcina_acetivorans_C2A |
| WP_011020214_1 | - | Class II terpene cyclase family protein | Methanosarcina_acetivorans_C2A |
| WP_011020215_1 | - | Predicted DNA-binding protein | Methanosarcina_acetivorans_C2A |
| WP_048064827_1 | - | Uncharacterized protein | Methanosarcina_acetivorans_C2A |
| WP_011020217_1 | - | Predicted DNA-binding protein | Methanosarcina_acetivorans_C2A |
| WP_011020218_1 | - | Homolog of Wybutosine (yW) biosynthesis enzyme, Fe-S oxidoreductase | Methanosarcina_acetivorans_C2A |
| WP_011020219_1 | Ftn | Ferritin | Methanosarcina_acetivorans_C2A |
| WP_011020220_1 | UspA | Nucleotide-binding protein, UspA family | Methanosarcina_acetivorans_C2A |
| WP_011020221_1 | - | Uncharacterized protein | Methanosarcina_acetivorans_C2A |
| WP_048064828_1 | - | Uncharacterized membrane protein | Methanosarcina_acetivorans_C2A |
| WP_011499439_1 | - | Predicted NADH:ubiquinone oxidoreductase, subunit RnfB fused to ferredoxin domains | Methanococcoides_burtonii_DSM_6242 |
| | | | Methanococcoides_burtonii_DSM_6242 |
| WP_011500088_1 | MgtC | Mg2+ transport system protein | Methanococcoides_burtonii_DSM_6242 |
| WP_011500089_1 | - | Predicted Fe-Mo cluster-binding protein, NifX family | Methanococcoides_burtonii_DSM_6242 |
| WP_011500090_1 | - | Metal-dependent hydrolase of the beta-lactamase superfamily II | Methanococcoides_burtonii_DSM_6242 |
| WP_011500091_1 | - | Uncharacterized metal-binding protein, DUF1847 family | Methanococcoides_burtonii_DSM_6242 |
| WP_011500092_1 | - | MinD superfamily P-loop ATPase containing an inserted ferredoxin domain | Methanococcoides_burtonii_DSM_6242 |
| WP_083755001_1 | - | MinD superfamily P-loop ATPase containing an inserted ferredoxin domain | Methanococcoides_burtonii_DSM_6242 |
| WP_011500093_1 | - | Predicted Fe-Mo cluster-binding protein, NifX family | Methanococcoides_burtonii_DSM_6242 |
| WP_011500094_1 | - | Predicted Fe-Mo cluster-binding protein, NifX family | Methanococcoides_burtonii_DSM_6242 |
| WP_011500095_1 | UppP | Undecaprenyl pyrophosphate phosphatase | Methanococcoides_burtonii_DSM_6242 |
| WP_011500096_1 | MtbA | Methylcobalamin:coenzyme M methyltransferase | Methanococcoides_burtonii_DSM_6242 |
| WP_011500097_1 | PylD | (3R)-3-methyl-D-ornithyl-N6-L-lysine dehydrogenase, pyrrolysine biosynthesis enzyme | Methanococcoides_burtonii_DSM_6242 |
| WP_011500098_1 | PylC | (3R)-3-methyl-D-ornithine:L-lysine ligase, pyrrolysine biosynthesis protein | Methanococcoides_burtonii_DSM_6242 |

| | | | |
|---|---|---|---|
| WP_011500099_1 | PylB/HcgA | (2R,3R)-3-methylornithine synthase involved in pyrrolysine biosynthesis/Iron-guanylylpyridinol (FeGP) cofactor biosynthesis protein HcgA | Methanococcoides_burtonii_DSM_6242 |
| WP_011500100_1 | PylS | Pyrrolysyl-tRNA-synthetase | Methanococcoides_burtonii_DSM_6242 |
| WP_048063660_1 | HisC | Histidinol-phosphate/aromatic aminotransferase or cobyric acid decarboxylase | Methanococcoides_burtonii_DSM_6242 |
| WP_011500102_1 | - | GTP:adenosylcobinamide-phosphate guanylyltransferase | Methanococcoides_burtonii_DSM_6242 |
| WP_011500103_1 | HisC | Histidinol-phosphate/aromatic aminotransferase or cobyric acid decarboxylase | Methanococcoides_burtonii_DSM_6242 |
| WP_011500104_1 | CbiB | Cobalamin biosynthesis protein CobD/CbiB | Methanococcoides_burtonii_DSM_6242 |
| WP_011500105_1 | PgpA | Phosphatidylglycerophosphatase A fused to adenosylcobinamide amidohydrolase, CbiZ | Methanococcoides_burtonii_DSM_6242 |
| WP_011500106_1 | CobS | Cobalamin-5-phosphate synthase | Methanococcoides_burtonii_DSM_6242 |
| WP_048063389_1 | - | GTP:adenosylcobinamide-phosphate guanylyltransferase | Methanococcoides_burtonii_DSM_6242 |
| WP_011500108_1 | - | Uncharacterized protein | Methanococcoides_burtonii_DSM_6242 |
| WP_011500109_1 | KaiC | KaiC family ATPase implicated in signal transduction | Methanococcoides_burtonii_DSM_6242 |
| WP_011500110_1 | - | Queuine tRNA-ribosyltransferase related protein | Methanococcoides_burtonii_DSM_6242 |
| | | | Methanohalophilus_mahii_DSM_5219 |
| WP_013036745_1 | MgtC | Mg2+ transport system protein | Methanohalophilus_mahii_DSM_5219 |
| WP_013036746_1 | - | Ferritin-like domain | Methanohalophilus_mahii_DSM_5219 |
| WP_013036747_1 | - | Metal-dependent hydrolase of the beta-lactamase superfamily II | Methanohalophilus_mahii_DSM_5219 |
| WP_013036748_1 | - | Uncharacterized metal-binding protein, DUF1847 family | Methanohalophilus_mahii_DSM_5219 |
| WP_013036749_1 | - | MinD superfamily P-loop ATPase containing an inserted ferredoxin domain | Methanohalophilus_mahii_DSM_5219 |
| WP_013036750_1 | - | MinD superfamily P-loop ATPase containing an inserted ferredoxin domain | Methanohalophilus_mahii_DSM_5219 |
| WP_013036751_1 | FrhB | Coenzyme F420-reducing hydrogenase, beta subunit fused to dissimilatory sulfite reductase | Methanohalophilus_mahii_DSM_5219 |
| WP_013036752_1 | - | Predicted Fe-Mo cluster-binding protein, NifX family | Methanohalophilus_mahii_DSM_5219 |
| WP_013036753_1 | - | Predicted Fe-Mo cluster-binding protein, NifX family | Methanohalophilus_mahii_DSM_5219 |
| WP_013036754_1 | MtbA | Methylcobalamin:coenzyme M methyltransferase | Methanohalophilus_mahii_DSM_5219 |
| WP_013036755_1 | PylD | (3R)-3-methyl-D-ornithyl-N6-L-lysine dehydrogenase, pyrrolysine biosynthesis enzyme | Methanohalophilus_mahii_DSM_5219 |
| WP_013036756_1 | PylC | (3R)-3-methyl-D-ornithine:L-lysine ligase, pyrrolysine biosynthesis protein | Methanohalophilus_mahii_DSM_5219 |
| WP_048902069_1 | PylB/HcgA | (2R,3R)-3-methylornithine synthase involved in pyrrolysine biosynthesis/Iron-guanylylpyridinol (FeGP) cofactor biosynthesis protein HcgA | Methanohalophilus_mahii_DSM_5219 |
| WP_013036758_1 | PylS | Pyrrolysyl-tRNA-synthetase | Methanohalophilus_mahii_DSM_5219 |
| WP_013036759_1 | HisC | Histidinol-phosphate/aromatic aminotransferase or cobyric acid decarboxylase | Methanohalophilus_mahii_DSM_5219 |
| WP_013036760_1 | - | Signal transduction histidine kinase, contains REC and PAS domains | Methanohalophilus_mahii_DSM_5219 |
| WP_013036761_1 | - | Queuine tRNA-ribosyltransferase related protein | Methanohalophilus_mahii_DSM_5219 |

| | | | |
|---|---|---|---|
| WP_013036762_1 | Sta1 | Viral transcriptional activator, contains HTH domain | Methanohalophilus_mahii_DSM_5219 |
| WP_013036763_1 | - | MFS family permease | Methanohalophilus_mahii_DSM_5219 |
| WP_013036764_1 | LabA | NYN domain, predicted PIN-related RNAse, tRNA/rRNA maturation | Methanohalophilus_mahii_DSM_5219 |
| WP_013036765_1 | - | Aspartate/tyrosine/aromatic aminotransferase | Methanohalophilus_mahii_DSM_5219 |
| WP_013036766_1 | - | DNA-binding transcriptional regulator, Lrp family | Methanohalophilus_mahii_DSM_5219 |
| WP_013036767_1 | RPS8A | Ribosomal protein S8E | Methanohalophilus_mahii_DSM_5219 |
| WP_048902225_1 | Rnz | Ribonuclease Z, beta-lactamase superfamily hydrolase | Methanohalophilus_mahii_DSM_5219 |
| | | | Methanohalobium_evestigatum_Z-7303 |
| WP_013193794_1 | MscS | Small-conductance mechanosensitive channel | Methanohalobium_evestigatum_Z-7303 |
| WP_013193795_1 | - | Uncharacterized protein | Methanohalobium_evestigatum_Z-7303 |
| WP_013193796_1 | UspA | Nucleotide-binding protein, UspA family | Methanohalobium_evestigatum_Z-7303 |
| WP_013193797_1 | UspA | Nucleotide-binding protein, UspA family | Methanohalobium_evestigatum_Z-7303 |
| WP_013193798_1 | CysG | Uroporphyrinogen-III methylase | Methanohalobium_evestigatum_Z-7303 |
| WP_013193799_1 | HemD | Uroporphyrinogen-III synthase | Methanohalobium_evestigatum_Z-7303 |
| WP_013193800_1 | - | Radical SAM superfamily enzyme | Methanohalobium_evestigatum_Z-7303 |
| WP_013193801_1 | - | Desulfoferredoxin | Methanohalobium_evestigatum_Z-7303 |
| WP_013193802_1 | Ftn | Ferritin | Methanohalobium_evestigatum_Z-7303 |
| WP_013193803_1 | ValS | Valyl-tRNA synthetase | Methanohalobium_evestigatum_Z-7303 |
| WP_013193804_1 | PylS | Pyrrolysyl-tRNA-synthetase | Methanohalobium_evestigatum_Z-7303 |
| WP_013193805_1 | MtbA | Methylcobalamin:coenzyme M methyltransferase | Methanohalobium_evestigatum_Z-7303 |
| WP_013193806_1 | SerA | Phosphoglycerate dehydrogenase or related dehydrogenase | Methanohalobium_evestigatum_Z-7303 |
| WP_013193807_1 | - | Uncharacterized protein | Methanohalobium_evestigatum_Z-7303 |
| WP_083810090_1 | - | Predicted HTH domain, homologous to N-terminal domain of RPA1 protein family | Methanohalobium_evestigatum_Z-7303 |
| WP_013193809_1 | RPL18A | Ribosomal protein L18E | Methanohalobium_evestigatum_Z-7303 |
| WP_013193810_1 | RplM | Ribosomal protein L13 | Methanohalobium_evestigatum_Z-7303 |
| WP_013193811_1 | RpsI | Ribosomal protein S9 | Methanohalobium_evestigatum_Z-7303 |
| WP_013193812_1 | RPB10 | DNA-directed RNA polymerase, subunit N (RpoN/RPB10) | Methanohalobium_evestigatum_Z-7303 |
| WP_013193813_1 | Rpo6/RpoZ | DNA-directed RNA polymerase subunit K/omega | Methanohalobium_evestigatum_Z-7303 |
| WP_013193814_1 | RpsB | Ribosomal protein S2 | Methanohalobium_evestigatum_Z-7303 |
| | | | Methanosalsum_zhilinae_DSM_4017 |
| WP_013898310_1 | RplM | Ribosomal protein L13 | Methanosalsum_zhilinae_DSM_4017 |
| WP_013898311_1 | RPL18A | Ribosomal protein L18E | Methanosalsum_zhilinae_DSM_4017 |
| WP_083812323_1 | - | Predicted HTH domain, homologous to N-terminal domain of RPA1 protein family | Methanosalsum_zhilinae_DSM_4017 |
| WP_013898313_1 | - | Uncharacterized protein | Methanosalsum_zhilinae_DSM_4017 |

| | | | |
|---|---|---|---|
| WP_013898314_1 | SerA | Phosphoglycerate dehydrogenase or related dehydrogenase | Methanosalsum_zhilinae_DSM_4017 |
| WP_013898315_1 | MtbA | Methylcobalamin:coenzyme M methyltransferase | Methanosalsum_zhilinae_DSM_4017 |
| WP_013898316_1 | - | Uncharacterized Fe-S clusters-containing protein, contains DUF4445 domain | Methanosalsum_zhilinae_DSM_4017 |
| WP_013898317_1 | - | NhaP-type Na+/H+ and K+/H+ antiporter with a unique C-terminal domain | Methanosalsum_zhilinae_DSM_4017 |
| WP_013898318_1 | UspA | Nucleotide-binding protein,  UspA family | Methanosalsum_zhilinae_DSM_4017 |
| WP_013898319_1 | - | GYD domain, alpha/beta barrel superfamily | Methanosalsum_zhilinae_DSM_4017 |
| WP_013898320_1 | PylS | Pyrrolysyl-tRNA-synthetase | Methanosalsum_zhilinae_DSM_4017 |
| WP_048815492_1 | PylB/HcgA | (2R,3R)-3-methylornithine synthase involved in pyrrolysine biosynthesis/Iron-guanylylpyridinol (FeGP) cofactor biosynthesis protein HcgA | Methanosalsum_zhilinae_DSM_4017 |
| WP_013898322_1 | PylC | (3R)-3-methyl-D-ornithine:L-lysine ligase, pyrrolysine biosynthesis protein | Methanosalsum_zhilinae_DSM_4017 |
| WP_013898323_1 | PylD | (3R)-3-methyl-D-ornithyl-N6-L-lysine dehydrogenase, pyrrolysine biosynthesis enzyme | Methanosalsum_zhilinae_DSM_4017 |
| WP_013898324_1 | IbpA | Molecular chaperone (HSP20 family) | Methanosalsum_zhilinae_DSM_4017 |
| WP_013898325_1 | - | Uncharacterized protein | Methanosalsum_zhilinae_DSM_4017 |
| WP_013898326_1 | NuoL | NADH dehydrogenase subunit  L | Methanosalsum_zhilinae_DSM_4017 |
| WP_013898327_1 | MnhB | Multisubunit Na+/H+ antiporter, MnhB subunit | Methanosalsum_zhilinae_DSM_4017 |
| WP_013898328_1 | MnhC | Multisubunit Na+/H+ antiporter, MnhC subunit | Methanosalsum_zhilinae_DSM_4017 |
| WP_013898329_1 | NuoM | NADH:ubiquinone oxidoreductase subunit 4 (chain M) | Methanosalsum_zhilinae_DSM_4017 |
| WP_013898330_1 | MnhE | Multisubunit Na+/H+ antiporter, MnhE subunit | Methanosalsum_zhilinae_DSM_4017 |
| WP_013898331_1 | MnhF | Multisubunit Na+/H+ antiporter, MnhF subunit | Methanosalsum_zhilinae_DSM_4017 |
| WP_083812326_1 | MnhG | Multisubunit Na+/H+ antiporter, MnhG subunit | Methanosalsum_zhilinae_DSM_4017 |
| WP_013898333_1 | SUA5 | tRNA A37 threonylcarbamoyladenosine synthetase subunit TsaC/SUA5/YrdC | Methanosalsum_zhilinae_DSM_4017 |
| | | | Candidatus_Methanomethylophilus_alvus_Mx1201 |
| WP_015504995_1 | #N/A | #N/A | Candidatus_Methanomethylophilus_alvus_Mx1201 |
| WP_048097823_1 | #N/A | #N/A | Candidatus_Methanomethylophilus_alvus_Mx1201 |
| WP_015504997_1 | #N/A | #N/A | Candidatus_Methanomethylophilus_alvus_Mx1201 |
| WP_048097824_1 | #N/A | #N/A | Candidatus_Methanomethylophilus_alvus_Mx1201 |
| WP_048097825_1 | #N/A | #N/A | Candidatus_Methanomethylophilus_alvus_Mx1201 |
| WP_048098005_1 | - | AAA family ATPase | Candidatus_Methanomethylophilus_alvus_Mx1201 |
| WP_048097826_1 | - | AAA family ATPase | Candidatus_Methanomethylophilus_alvus_Mx1201 |
| WP_048097827_1 | - | Mg-chelatase subunit ChlD | Candidatus_Methanomethylophilus_alvus_Mx1201 |
| WP_015505003_1 | SrmB | Superfamily II DNA and RNA helicase | Candidatus_Methanomethylophilus_alvus_Mx1201 |
| WP_048097828_1 | - | Uncharacterized Fe-S clusters-containing protein, contains DUF4445 domain | Candidatus_Methanomethylophilus_alvus_Mx1201 |
| WP_015505006_1 | PylD | (3R)-3-methyl-D-ornithyl-N6-L-lysine dehydrogenase, pyrrolysine biosynthesis enzyme | Candidatus_Methanomethylophilus_alvus_Mx1201 |
| WP_015505007_1 | PylC | (3R)-3-methyl-D-ornithine:L-lysine ligase, pyrrolysine biosynthesis protein | Candidatus_Methanomethylophilus_alvus_Mx1201 |

| | | | |
|---|---|---|---|
| WP_015505008_1 | PylS | Pyrrolysyl-tRNA-synthetase | Candidatus_Methanomethylophilus_alvus_Mx1201 |
| WP_048098006_1 | MtmB | Monomethylamine methyltransferase | Candidatus_Methanomethylophilus_alvus_Mx1201 |
| WP_048097829_1 | MtmB | Monomethylamine methyltransferase | Candidatus_Methanomethylophilus_alvus_Mx1201 |
| WP_048098007_1 | MtmB | Monomethylamine methyltransferase | Candidatus_Methanomethylophilus_alvus_Mx1201 |
| WP_048097830_1 | MtmB | Monomethylamine methyltransferase | Candidatus_Methanomethylophilus_alvus_Mx1201 |
| WP_015505011_1 | MtbC1 | Methanogenic corrinoid protein MtbC1 | Candidatus_Methanomethylophilus_alvus_Mx1201 |
| WP_015505013_1 | - | Uncharacterized protein | Candidatus_Methanomethylophilus_alvus_Mx1201 |
| WP_015505014_1 | RhaT | Permease of the drug/metabolite transporter (DMT) superfamily | Candidatus_Methanomethylophilus_alvus_Mx1201 |
| WP_015505015_1 | MtbC1 | Methanogenic corrinoid protein MtbC1 | Candidatus_Methanomethylophilus_alvus_Mx1201 |
| WP_048098010_1 | MttB1 | Trimethylamine:corrinoid methyltransferase | Candidatus_Methanomethylophilus_alvus_Mx1201 |
| WP_048097831_1 | MttB2 | Trimethylamine:corrinoid methyltransferase | Candidatus_Methanomethylophilus_alvus_Mx1201 |
| WP_048097832_1 | MtbB | Dimethylamine methyltransferase | Candidatus_Methanomethylophilus_alvus_Mx1201 |
| WP_048097833_1 | MtbB | Dimethylamine methyltransferase | Candidatus_Methanomethylophilus_alvus_Mx1201 |
| WP_015505018_1 | MtbC1 | Methanogenic corrinoid protein MtbC1 | Candidatus_Methanomethylophilus_alvus_Mx1201 |
| WP_048097834_1 | PotE | Amino acid transporter | Candidatus_Methanomethylophilus_alvus_Mx1201 |
| WP_015505020_1 | FrvX | Peptidase M42 family protein | Candidatus_Methanomethylophilus_alvus_Mx1201 |
| WP_015505021_1 | UbiE | Ubiquinone/menaquinone biosynthesis C-methylase UbiE | Candidatus_Methanomethylophilus_alvus_Mx1201 |
| WP_015505022_1 | MenA | 1,4-dihydroxy-2-naphthoate octaprenyltransferase | Candidatus_Methanomethylophilus_alvus_Mx1201 |
| WP_015505023_1 | NapF | Flavodoxin fused to ferredoxin domain | Candidatus_Methanomethylophilus_alvus_Mx1201 |
| WP_015505024_1 | - | Predicted Na+-dependent transporter | Candidatus_Methanomethylophilus_alvus_Mx1201 |
| WP_048097836_1 | - | DNA repair photolyase | Candidatus_Methanomethylophilus_alvus_Mx1201 |
| WP_015505026_1 | Ada | Methylated DNA-protein cysteine methyltransferase | Candidatus_Methanomethylophilus_alvus_Mx1201 |
| WP_015505027_1 | AlkD | 3-methyladenine DNA glycosylase AlkD | Candidatus_Methanomethylophilus_alvus_Mx1201 |
| | | | Methanolobus_psychrophilus_R15 |
| WP_015053620_1 | PotE | Amino acid transporter | Methanolobus_psychrophilus_R15 |
| WP_015053602_1 | MtbC1 | Methanogenic corrinoid protein MtbC1 | Methanolobus_psychrophilus_R15 |
| WP_015053623_1 | MtbC1 | Methanogenic corrinoid protein MtbC1 | Methanolobus_psychrophilus_R15 |
| WP_015053624_1 | MtbA | Methylcobalamin:coenzyme M methyltransferase | Methanolobus_psychrophilus_R15 |
| WP_015053625_1 | MtbA | Methylcobalamin:coenzyme M methyltransferase | Methanolobus_psychrophilus_R15 |
| WP_015053626_1 | - | Desulfoferredoxin | Methanolobus_psychrophilus_R15 |
| WP_015053627_1 | AcrB | Cation/multidrug efflux pump | Methanolobus_psychrophilus_R15 |
| WP_048147553_1 | - | Uncharacterized membrane protein | Methanolobus_psychrophilus_R15 |
| WP_048146974_1 | PotE | Amino acid transporter | Methanolobus_psychrophilus_R15 |
| WP_015053633_1 | MtmB | Monomethylamine methyltransferase | Methanolobus_psychrophilus_R15 |
| WP_015053634_1 | MtbC1 | Methanogenic corrinoid protein MtbC1 | Methanolobus_psychrophilus_R15 |

| | | | |
|---|---|---|---|
| WP_015053635_1 | - | Transcriptional regulator, contains HTH domain | Methanolobus_psychrophilus_R15 |
| WP_015053636_1 | - | Transcriptional regulator, contains HTH domain | Methanolobus_psychrophilus_R15 |
| WP_015053637_1 | - | Signal transduction histidine kinase, contains PAS domains | Methanolobus_psychrophilus_R15 |
| WP_015053638_1 | CheY | Rec and PAS domains | Methanolobus_psychrophilus_R15 |
| WP_015053640_1 | PylS | Pyrrolysyl-tRNA-synthetase | Methanolobus_psychrophilus_R15 |
| WP_015053641_1 | PylB/HcgA | (2R,3R)-3-methylornithine synthase involved in pyrrolysine biosynthesis/Iron-guanylylpyridinol (FeGP) cofactor biosynthesis protein HcgA | Methanolobus_psychrophilus_R15 |
| WP_015053642_1 | PylC | (3R)-3-methyl-D-ornithine:L-lysine ligase, pyrrolysine biosynthesis protein | Methanolobus_psychrophilus_R15 |
| WP_015053643_1 | PylD | (3R)-3-methyl-D-ornithyl-N6-L-lysine dehydrogenase, pyrrolysine biosynthesis enzyme | Methanolobus_psychrophilus_R15 |
| WP_015053644_1 | - | Uncharacterized Fe-S clusters-containing protein, contains DUF4445 domain | Methanolobus_psychrophilus_R15 |
| WP_048147555_1 | HYS2 | Archaeal DNA polymerase II, small subunit/DNA polymerase delta, subunit B | Methanolobus_psychrophilus_R15 |
| WP_015053646_1 | LepB | Signal peptidase I | Methanolobus_psychrophilus_R15 |
| WP_015053647_1 | CDC6 | Cdc6-related protein, AAA superfamily ATPase | Methanolobus_psychrophilus_R15 |
| WP_048146976_1 | MfnA | L-tyrosine decarboxylase, PLP-dependent protein | Methanolobus_psychrophilus_R15 |
| WP_048146978_1 | - | Zn-dependent protease fused to CBS domain | Methanolobus_psychrophilus_R15 |
| WP_015053651_1 | - | Pheromone shutdown protein TraB, contains GTxH motif | Methanolobus_psychrophilus_R15 |
| WP_015053652_1 | - | Transglutaminase-like cysteine protease | Methanolobus_psychrophilus_R15 |
| WP_015053653_1 | Ftr | Formylmethanofuran:tetrahydromethanopterin formyltransferase | Methanolobus_psychrophilus_R15 |
| WP_015053654_1 | - | Uncharacterized membrane protein | Methanolobus_psychrophilus_R15 |
| | | | Methanomethylovorans_hollandica_DSM_15978 |
| WP_015323476_1 | MtbA | Methylcobalamin:coenzyme M methyltransferase | Methanomethylovorans_hollandica_DSM_15978 |
| WP_015323477_1 | - | Desulfoferredoxin | Methanomethylovorans_hollandica_DSM_15978 |
| WP_015323478_1 | AcrB | Cation/multidrug efflux pump | Methanomethylovorans_hollandica_DSM_15978 |
| WP_015323479_1 | - | Uncharacterized membrane protein | Methanomethylovorans_hollandica_DSM_15978 |
| WP_015323480_1 | PotE | Amino acid transporter | Methanomethylovorans_hollandica_DSM_15978 |
| WP_015323483_1 | MtbC1 | Methanogenic corrinoid protein MtbC1 | Methanomethylovorans_hollandica_DSM_15978 |
| WP_015323484_1 | - | Transcriptional regulator, contains HTH domain | Methanomethylovorans_hollandica_DSM_15978 |
| WP_015323485_1 | - | Transcriptional regulator, contains HTH domain | Methanomethylovorans_hollandica_DSM_15978 |
| WP_015323486_1 | - | Signal transduction histidine kinase, contains PAS domains | Methanomethylovorans_hollandica_DSM_15978 |
| WP_015323487_1 | CheY | Rec and PAS domains | Methanomethylovorans_hollandica_DSM_15978 |
| WP_015323488_1 | PylD | (3R)-3-methyl-D-ornithyl-N6-L-lysine dehydrogenase, pyrrolysine biosynthesis enzyme | Methanomethylovorans_hollandica_DSM_15978 |
| WP_015323489_1 | PylC | (3R)-3-methyl-D-ornithine:L-lysine ligase, pyrrolysine biosynthesis protein | Methanomethylovorans_hollandica_DSM_15978 |
| WP_015323490_1 | PylB/HcgA | (2R,3R)-3-methylornithine synthase involved in pyrrolysine biosynthesis/Iron-guanylylpyridinol (FeGP) cofactor biosynthesis protein HcgA | Methanomethylovorans_hollandica_DSM_15978 |

| | | | |
|---|---|---|---|
| WP_015323491_1 | PylS | Pyrrolysyl-tRNA-synthetase | Methanomethylovorans_hollandica_DSM_15978 |
| WP_015323492_1 | HyuA | N-methylhydantoinase A/acetone carboxylase, beta subunit | Methanomethylovorans_hollandica_DSM_15978 |
| WP_015323493_1 | HyuA | N-methylhydantoinase A/acetone carboxylase, beta subunit | Methanomethylovorans_hollandica_DSM_15978 |
| WP_015323494_1 | - | Uncharacterized protein, DUF1638 family | Methanomethylovorans_hollandica_DSM_15978 |
| WP_015323495_1 | - | Sensory protein, contains PAS and GAF domains | Methanomethylovorans_hollandica_DSM_15978 |
| WP_048830925_1 | MttB2 | Trimethylamine:corrinoid methyltransferase | Methanomethylovorans_hollandica_DSM_15978 |
| WP_048830927_1 | MttB1 | Trimethylamine:corrinoid methyltransferase | Methanomethylovorans_hollandica_DSM_15978 |
| WP_015323497_1 | MtbC1 | Methanogenic corrinoid protein MtbC1 | Methanomethylovorans_hollandica_DSM_15978 |
| WP_015323498_1 | RhaT | Permease of the drug/metabolite transporter (DMT) superfamily | Methanomethylovorans_hollandica_DSM_15978 |
| WP_015323499_1 | - | Uncharacterized protein | Methanomethylovorans_hollandica_DSM_15978 |
| WP_015323501_1 | MtbC1 | Methanogenic corrinoid protein MtbC1 | Methanomethylovorans_hollandica_DSM_15978 |
| WP_048830930_1 | MtbB | Dimethylamine methyltransferase | Methanomethylovorans_hollandica_DSM_15978 |
| WP_048830932_1 | MtbB | Dimethylamine methyltransferase | Methanomethylovorans_hollandica_DSM_15978 |
| WP_015323483_1 | MtbC1 | Methanogenic corrinoid protein MtbC1 | Methanomethylovorans_hollandica_DSM_15978 |
| WP_048830922_1 | MtmB | Monomethylamine methyltransferase | Methanomethylovorans_hollandica_DSM_15978 |
| WP_083885671_1 | MtmB | Monomethylamine methyltransferase | Methanomethylovorans_hollandica_DSM_15978 |
| WP_015323504_1 | MtmB | Monomethylamine methyltransferase | Methanomethylovorans_hollandica_DSM_15978 |
| WP_015323476_1 | MtbA | Methylcobalamin:coenzyme M methyltransferase | Methanomethylovorans_hollandica_DSM_15978 |
| WP_015323505_1 | TYW1 | Wybutosine (yW) biosynthesis enzyme, Fe-S oxidoreductase | Methanomethylovorans_hollandica_DSM_15978 |
| WP_015323506_1 | eRF1 | Peptide chain release factor eRF1 | Methanomethylovorans_hollandica_DSM_15978 |
| WP_015323507_1 | ArgS | Arginyl-tRNA synthetase | Methanomethylovorans_hollandica_DSM_15978 |
| WP_015323508_1 | CcmA | ABC-type multidrug transport system, ATPase component | Methanomethylovorans_hollandica_DSM_15978 |
| WP_015323509_1 | NatB | ABC-type Na+ efflux pump, permease component | Methanomethylovorans_hollandica_DSM_15978 |
| WP_015323510_1 | NatB | ABC-type Na+ efflux pump, permease component | Methanomethylovorans_hollandica_DSM_15978 |
| WP_015323511_1 | - | Uncharacterized protein, contains DUF11 domain | Methanomethylovorans_hollandica_DSM_15978 |
| WP_015323512_1 | - | Uncharacterized membrane protein | Methanomethylovorans_hollandica_DSM_15978 |
| WP_015323513_1 | - | Toxic component of toxin-antitoxin system, dsRBD-like fold, HicA family | Methanomethylovorans_hollandica_DSM_15978 |
| | | | Thermoplasmatales_archaeon_BRNA1 |
| WP_015492585_1 | GRS1 | Glycyl-tRNA synthetase (class II) | Thermoplasmatales_archaeon_BRNA1 |
| WP_015492586_1 | - | Protein containing two CBS domains (some fused to C-terminal double-stranded RNA-binding domain of RaiA family) | Thermoplasmatales_archaeon_BRNA1 |
| WP_015492587_1 | ValS | Valyl-tRNA synthetase | Thermoplasmatales_archaeon_BRNA1 |
| WP_015492588_1 | - | Rubredoxin | Thermoplasmatales_archaeon_BRNA1 |
| WP_015492589_1 | - | Rubredoxin | Thermoplasmatales_archaeon_BRNA1 |
| WP_015492590_1 | SrmB | Superfamily II DNA and RNA helicase | Thermoplasmatales_archaeon_BRNA1 |

| | | | |
|---|---|---|---|
| WP_015492591_1 | EbsC | Cys-tRNA(Pro)/Cys-tRNA(Cys) deacylase, ybaK family | Thermoplasmatales_archaeon_BRNA1 |
| WP_015492592_1 | - | Uncharacterized membrane protein, DUF1846 family | Thermoplasmatales_archaeon_BRNA1 |
| WP_048163897_1 | SrmB | Superfamily II DNA and RNA helicase | Thermoplasmatales_archaeon_BRNA1 |
| WP_015492595_1 | - | Uncharacterized Fe-S clusters-containing protein, contains DUF4445 domain | Thermoplasmatales_archaeon_BRNA1 |
| WP_015492596_1 | PylD | (3R)-3-methyl-D-ornithyl-N6-L-lysine dehydrogenase, pyrrolysine biosynthesis enzyme | Thermoplasmatales_archaeon_BRNA1 |
| WP_015492597_1 | PylC | (3R)-3-methyl-D-ornithine:L-lysine ligase, pyrrolysine biosynthesis protein | Thermoplasmatales_archaeon_BRNA1 |
| WP_015492598_1 | PylS | Pyrrolysyl-tRNA-synthetase | Thermoplasmatales_archaeon_BRNA1 |
| WP_015492601_1 | MtbC1 | Methanogenic corrinoid protein MtbC1 | Thermoplasmatales_archaeon_BRNA1 |
| WP_015492602_1 | EmrE | Membrane transporter of cations and cationic drugs | Thermoplasmatales_archaeon_BRNA1 |
| WP_048164390_1 | EmrE | Membrane transporter of cations and cationic drugs | Thermoplasmatales_archaeon_BRNA1 |
| WP_015492605_1 | - | Uncharacterized protein | Thermoplasmatales_archaeon_BRNA1 |
| WP_015492606_1 | RhaT | Permease of the drug/metabolite transporter (DMT) superfamily | Thermoplasmatales_archaeon_BRNA1 |
| WP_015492607_1 | MtbC1 | Methanogenic corrinoid protein MtbC1 | Thermoplasmatales_archaeon_BRNA1 |
| WP_083893799_1 | MttB1 | Trimethylamine:corrinoid methyltransferase | Thermoplasmatales_archaeon_BRNA1 |
| WP_083893801_1 | MttB2 | Trimethylamine:corrinoid methyltransferase | Thermoplasmatales_archaeon_BRNA1 |
| WP_015492610_1 | MtbC1 | Methanogenic corrinoid protein MtbC1 | Thermoplasmatales_archaeon_BRNA1 |
| WP_048163911_1 | #N/A | #N/A | Thermoplasmatales_archaeon_BRNA1 |
| WP_015492611_1 | FrvX | Peptidase M42 family protein | Thermoplasmatales_archaeon_BRNA1 |
| WP_015492612_1 | UbiE | Ubiquinone/menaquinone biosynthesis C-methylase UbiE | Thermoplasmatales_archaeon_BRNA1 |
| WP_015492613_1 | MenA | 1,4-dihydroxy-2-naphthoate octaprenyltransferase | Thermoplasmatales_archaeon_BRNA1 |
| WP_015492614_1 | - | Predicted Na+-dependent transporter | Thermoplasmatales_archaeon_BRNA1 |
| WP_015492615_1 | - | DNA repair photolyase | Thermoplasmatales_archaeon_BRNA1 |
| WP_015492616_1 | Ada | Methylated DNA-protein cysteine methyltransferase | Thermoplasmatales_archaeon_BRNA1 |
| WP_015492617_1 | AlkD | 3-methyladenine DNA glycosylase AlkD | Thermoplasmatales_archaeon_BRNA1 |
| WP_015492618_1 | - | Uncharacterized protein | Thermoplasmatales_archaeon_BRNA1 |
| WP_081633107_1 | - | Superfamily I DNA/RNA helicase, contains Vsr family nuclease domain | Candidatus_Methanomassiliicoccus_intestinalis_Issoire-Mx1 |
| WP_020448765_1 | AlkD | 3-methyladenine DNA glycosylase AlkD | Candidatus_Methanomassiliicoccus_intestinalis_Issoire-Mx1 |
| WP_020448766_1 | - | DNA repair photolyase | Candidatus_Methanomassiliicoccus_intestinalis_Issoire-Mx1 |
| WP_020448767_1 | #N/A | #N/A | Candidatus_Methanomassiliicoccus_intestinalis_Issoire-Mx1 |
| WP_048134366_1 | 0 | 4-Hydroxybutyryl-CoA synthetase (AMP-forming) | Candidatus_Methanomassiliicoccus_intestinalis_Issoire-Mx1 |
| WP_020448769_1 | PorG | Pyruvate:ferredoxin oxidoreductase or related 2-oxoacid:ferredoxin oxidoreductase, gamma subunit | Candidatus_Methanomassiliicoccus_intestinalis_Issoire-Mx1 |
| WP_020448770_1 | - | Indolepyruvate ferredoxin oxidoreductase, alpha and beta subunit | Candidatus_Methanomassiliicoccus_intestinalis_Issoire-Mx1 |

| | | | |
|---|---|---|---|
| WP_020448771_1 | - | Uncharacterized secreted protein, contains kinase-interacting SIMPL domain | Candidatus_Methanomassiliicoccus_intestinalis_Issoire-Mx1 |
| WP_020448772_1 | MtbC1 | Methanogenic corrinoid protein MtbC1 | Candidatus_Methanomassiliicoccus_intestinalis_Issoire-Mx1 |
| WP_020448773_1 | - | Uncharacterized Fe-S clusters-containing protein, contains DUF4445 domain | Candidatus_Methanomassiliicoccus_intestinalis_Issoire-Mx1 |
| WP_081633108_1 | PylD | (3R)-3-methyl-D-ornithyl-N6-L-lysine dehydrogenase, pyrrolysine biosynthesis enzyme | Candidatus_Methanomassiliicoccus_intestinalis_Issoire-Mx1 |
| WP_020448775_1 | PylC | (3R)-3-methyl-D-ornithine:L-lysine ligase, pyrrolysine biosynthesis protein | Candidatus_Methanomassiliicoccus_intestinalis_Issoire-Mx1 |
| WP_020448776_1 | PylB/HcgA | (2R,3R)-3-methylornithine synthase involved in pyrrolysine biosynthesis/Iron-guanylylpyridinol (FeGP) cofactor biosynthesis protein HcgA | Candidatus_Methanomassiliicoccus_intestinalis_Issoire-Mx1 |
| WP_020448777_1 | PylS | Pyrrolysyl-tRNA-synthetase | Candidatus_Methanomassiliicoccus_intestinalis_Issoire-Mx1 |
| WP_020448778_1 | MtbC1 | Methanogenic corrinoid protein MtbC1 | Candidatus_Methanomassiliicoccus_intestinalis_Issoire-Mx1 |
| WP_048134372_1 | AmtB | Ammonia permease | Candidatus_Methanomassiliicoccus_intestinalis_Issoire-Mx1 |
| WP_020448780_1 | PotE | Amino acid transporter | Candidatus_Methanomassiliicoccus_intestinalis_Issoire-Mx1 |
| WP_081633170_1 | - | Uncharacterized protein | Candidatus_Methanomassiliicoccus_intestinalis_Issoire-Mx1 |
| WP_020448782_1 | MtbC1 | Methanogenic corrinoid protein MtbC1 | Candidatus_Methanomassiliicoccus_intestinalis_Issoire-Mx1 |
| WP_020448787_1 | MtbC1 | Methanogenic corrinoid protein MtbC1 | Candidatus_Methanomassiliicoccus_intestinalis_Issoire-Mx1 |
| WP_020448788_1 | NorV | Flavorubredoxin | Candidatus_Methanomassiliicoccus_intestinalis_Issoire-Mx1 |
| WP_048133942_1 | #N/A | #N/A | Candidatus_Methanomassiliicoccus_intestinalis_Issoire-Mx1 |
| WP_020448789_1 | NrdD | Oxygen-sensitive ribonucleoside-triphosphate reductase | Candidatus_Methanomassiliicoccus_intestinalis_Issoire-Mx1 |
| WP_081633109_1 | - | Uncharacterized membrane protein, a putative transporter component | Candidatus_Methanomassiliicoccus_intestinalis_Issoire-Mx1 |
| | | | Candidatus_Methanoplasma_termitum_MpT1 |
| WP_048111096_1 | - | Transposase | Candidatus_Methanoplasma_termitum_MpT1 |
| WP_048111882_1 | MenH | 2-succinyl-6-hydroxy-2,4-cyclohexadiene-1-carboxylate synthase MenH and related esterases, alpha/beta hydrolase fold | Candidatus_Methanoplasma_termitum_MpT1 |
| WP_048111884_1 | - | Fe-S-cluster containining protein | Candidatus_Methanoplasma_termitum_MpT1 |
| WP_048111886_1 | #N/A | #N/A | Candidatus_Methanoplasma_termitum_MpT1 |
| WP_082007231_1 | - | SAM-dependent methyltransferase | Candidatus_Methanoplasma_termitum_MpT1 |
| WP_082007309_1 | 0 | 4-Hydroxybutyryl-CoA synthetase (AMP-forming) | Candidatus_Methanoplasma_termitum_MpT1 |
| WP_048111893_1 | - | ACT domain containing protein | Candidatus_Methanoplasma_termitum_MpT1 |
| WP_048111894_1 | SelB | Selenocysteine-specific translation elongation factor or SelB-II domain | Candidatus_Methanoplasma_termitum_MpT1 |
| WP_048111896_1 | 0 | 4-Hydroxybutyryl-CoA synthetase (AMP-forming) | Candidatus_Methanoplasma_termitum_MpT1 |
| WP_048111898_1 | WrbA | Multimeric flavodoxin WrbA | Candidatus_Methanoplasma_termitum_MpT1 |
| WP_048111900_1 | PylD | (3R)-3-methyl-D-ornithyl-N6-L-lysine dehydrogenase, pyrrolysine biosynthesis enzyme | Candidatus_Methanoplasma_termitum_MpT1 |
| WP_052399354_1 | PylC | (3R)-3-methyl-D-ornithine:L-lysine ligase, pyrrolysine biosynthesis protein | Candidatus_Methanoplasma_termitum_MpT1 |
| WP_048111905_1 | PylB/HcgA | (2R,3R)-3-methylornithine synthase involved in pyrrolysine biosynthesis/Iron- | Candidatus_Methanoplasma_termitum_MpT1 |

| | | | |
|---|---|---|---|
| | | guanylylpyridinol (FeGP) cofactor biosynthesis protein HcgA | |
| WP_048111907_1 | PylS | Pyrrolysyl-tRNA-synthetase | Candidatus_Methanoplasma_termitum_MpT1 |
| WP_048111909_1 | #N/A | #N/A | Candidatus_Methanoplasma_termitum_MpT1 |
| WP_048113723_1 | AmtB | Ammonia permease | Candidatus_Methanoplasma_termitum_MpT1 |
| WP_048111918_1 | MtbC1 | Methanogenic corrinoid protein MtbC1 | Candidatus_Methanoplasma_termitum_MpT1 |
| WP_052399259_1 | - | TPR repeats containing protein | Candidatus_Methanoplasma_termitum_MpT1 |
| WP_052399260_1 | - | MFS family permease | Candidatus_Methanoplasma_termitum_MpT1 |
| WP_048111919_1 | - | Uncharacterized Fe-S center protein | Candidatus_Methanoplasma_termitum_MpT1 |
| WP_048113739_1 | SpeD | S-adenosylmethionine decarboxylase/arginine decarboxylase | Candidatus_Methanoplasma_termitum_MpT1 |
| WP_048111921_1 | - | Uncharacterized protein | Candidatus_Methanoplasma_termitum_MpT1 |
| WP_048111925_1 | - | Predicted pilin/flagellin | Candidatus_Methanoplasma_termitum_MpT1 |
| WP_048111927_1 | - | Predicted pilin/flagellin | Candidatus_Methanoplasma_termitum_MpT1 |
| | | | Methanosarcina_thermophila_TM-1 |
| WP_048167680_1 | SecD | Preprotein translocase subunit SecD | Methanosarcina_thermophila_TM-1 |
| WP_048167681_1 | RFCS | Clamp loader ATPase, small subunit | Methanosarcina_thermophila_TM-1 |
| WP_048167682_1 | #N/A | #N/A | Methanosarcina_thermophila_TM-1 |
| WP_048167683_1 | AcrB | Cation/multidrug efflux pump | Methanosarcina_thermophila_TM-1 |
| WP_048167684_1 | - | Uncharacterized membrane protein | Methanosarcina_thermophila_TM-1 |
| WP_048167685_1 | PotE | Amino acid transporter | Methanosarcina_thermophila_TM-1 |
| WP_048167688_1 | MtbC1 | Methanogenic corrinoid protein MtbC1 | Methanosarcina_thermophila_TM-1 |
| WP_048167689_1 | MtbA | Methylcobalamin:coenzyme M methyltransferase | Methanosarcina_thermophila_TM-1 |
| WP_048167690_1 | - | SAM-dependent methyltransferase | Methanosarcina_thermophila_TM-1 |
| WP_048167691_1 | - | Uncharacterized Fe-S clusters-containing protein, contains DUF4445 domain | Methanosarcina_thermophila_TM-1 |
| WP_048167692_1 | PylD | (3R)-3-methyl-D-ornithyl-N6-L-lysine dehydrogenase, pyrrolysine biosynthesis enzyme | Methanosarcina_thermophila_TM-1 |
| WP_048167693_1 | PylC | (3R)-3-methyl-D-ornithine:L-lysine ligase, pyrrolysine biosynthesis protein | Methanosarcina_thermophila_TM-1 |
| WP_048167694_1 | PylB/HcgA | (2R,3R)-3-methylornithine synthase involved in pyrrolysine biosynthesis/Iron-guanylylpyridinol (FeGP) cofactor biosynthesis protein HcgA | Methanosarcina_thermophila_TM-1 |
| WP_048167695_1 | PylS | Pyrrolysyl-tRNA-synthetase | Methanosarcina_thermophila_TM-1 |
| WP_048167696_1 | - | Uncharacterized protein, DUF1638 family | Methanosarcina_thermophila_TM-1 |
| WP_048167697_1 | Ftn | Ferritin | Methanosarcina_thermophila_TM-1 |
| WP_048167698_1 | UspA | Nucleotide-binding protein, UspA family | Methanosarcina_thermophila_TM-1 |
| WP_048167699_1 | UspA | Nucleotide-binding protein, UspA family | Methanosarcina_thermophila_TM-1 |
| WP_048167700_1 | - | Uncharacterized protein | Methanosarcina_thermophila_TM-1 |
| WP_048167701_1 | YcaO | Ribosomal protein S12 methylthiotransferase accessory factor YcaO | Methanosarcina_thermophila_TM-1 |

| | | | |
|---|---|---|---|
| WP_048167702_1 | - | Uncharacterized protein, DUF2892 family | Methanosarcina_thermophila_TM-1 |
| WP_082086883_1 | MgtA | Cation transport ATPase | Methanosarcina_thermophila_TM-1 |
| WP_082086884_1 | ZntA | Cation transport ATPase | Methanosarcina_thermophila_TM-1 |
| WP_048167703_1 | - | Uncharacterized protein | Methanosarcina_thermophila_TM-1 |
| | | | Methanosarcina_vacuolata_Z-761 |
| WP_048123145_1 | - | Uncharacterized protein | Methanosarcina_vacuolata_Z-761 |
| WP_048123147_1 | UspA | Nucleotide-binding protein, UspA family | Methanosarcina_vacuolata_Z-761 |
| WP_048123149_1 | UspA | Nucleotide-binding protein, UspA family | Methanosarcina_vacuolata_Z-761 |
| WP_048124320_1 | Ftn | Ferritin | Methanosarcina_vacuolata_Z-761 |
| WP_048123151_1 | - | Predicted DNA-binding protein | Methanosarcina_vacuolata_Z-761 |
| WP_048123153_1 | SqhC | Squalene cyclase | Methanosarcina_vacuolata_Z-761 |
| WP_048123155_1 | - | Uncharacterized protein, DUF1638 family | Methanosarcina_vacuolata_Z-761 |
| WP_048123158_1 | - | Uncharacterized protein, DUF1638 family | Methanosarcina_vacuolata_Z-761 |
| WP_048123161_1 | AcrR | Transcriptional regulator, TetR/AcrR family | Methanosarcina_vacuolata_Z-761 |
| WP_048123163_1 | #N/A | #N/A | Methanosarcina_vacuolata_Z-761 |
| WP_048123165_1 | PylD | (3R)-3-methyl-D-ornithyl-N6-L-lysine dehydrogenase, pyrrolysine biosynthesis enzyme | Methanosarcina_vacuolata_Z-761 |
| WP_048123167_1 | PylC | (3R)-3-methyl-D-ornithine:L-lysine ligase, pyrrolysine biosynthesis protein | Methanosarcina_vacuolata_Z-761 |
| WP_048123169_1 | PylB/HcgA | (2R,3R)-3-methylornithine synthase involved in pyrrolysine biosynthesis/Iron-guanylylpyridinol (FeGP) cofactor biosynthesis protein HcgA | Methanosarcina_vacuolata_Z-761 |
| WP_048123171_1 | PylS | Pyrrolysyl-tRNA-synthetase | Methanosarcina_vacuolata_Z-761 |
| WP_048123173_1 | - | Uncharacterized Fe-S clusters-containing protein, contains DUF4445 domain | Methanosarcina_vacuolata_Z-761 |
| WP_048123175_1 | #N/A | #N/A | Methanosarcina_vacuolata_Z-761 |
| WP_048123177_1 | MtbA | Methylcobalamin:coenzyme M methyltransferase | Methanosarcina_vacuolata_Z-761 |
| WP_082091188_1 | - | Pentapeptide repeats containing protein | Methanosarcina_vacuolata_Z-761 |
| WP_048124322_1 | MtbC1 | Methanogenic corrinoid protein MtbC1 | Methanosarcina_vacuolata_Z-761 |
| WP_048123179_1 | MtmB | Monomethylamine methyltransferase | Methanosarcina_vacuolata_Z-761 |
| WP_082091189_1 | MtmB | Monomethylamine methyltransferase | Methanosarcina_vacuolata_Z-761 |
| WP_048123184_1 | PotE | Amino acid transporter | Methanosarcina_vacuolata_Z-761 |
| WP_048123186_1 | - | Uncharacterized membrane protein | Methanosarcina_vacuolata_Z-761 |
| WP_048123188_1 | AcrB | Cation/multidrug efflux pump | Methanosarcina_vacuolata_Z-761 |
| WP_048123190_1 | RFCS | Clamp loader ATPase, small subunit | Methanosarcina_vacuolata_Z-761 |
| WP_048123191_1 | SecD | Preprotein translocase subunit SecD | Methanosarcina_vacuolata_Z-761 |
| WP_048123193_1 | SecF | Preprotein translocase subunit SecF | Methanosarcina_vacuolata_Z-761 |
| WP_048123195_1 | ComEB | Deoxycytidylate deaminase | Methanosarcina_vacuolata_Z-761 |

| | | | |
|---|---|---|---|
| WP_048123197_1 | - | Cupin domain containing protein | Methanosarcina_vacuolata_Z-761 |
| WP_048123199_1 | CofE | F(420)-0:gamma-glutamyl ligase, F420 coenzyme biosynthesis enzyme | Methanosarcina_vacuolata_Z-761 |
| WP_048123201_1 | - | Uncharacterized protein | Methanosarcina_vacuolata_Z-761 |
| | | | Methanosarcina_sp_Kolksee |
| WP_048157953_1 | - | Uncharacterized protein | Methanosarcina_sp_Kolksee |
| WP_048123147_1 | UspA | Nucleotide-binding protein, UspA family | Methanosarcina_sp_Kolksee |
| WP_048123149_1 | UspA | Nucleotide-binding protein, UspA family | Methanosarcina_sp_Kolksee |
| WP_048158454_1 | Ftn | Ferritin | Methanosarcina_sp_Kolksee |
| WP_048157954_1 | - | Predicted DNA-binding protein | Methanosarcina_sp_Kolksee |
| WP_048123153_1 | SqhC | Squalene cyclase | Methanosarcina_sp_Kolksee |
| WP_048157955_1 | - | Uncharacterized protein, DUF1638 family | Methanosarcina_sp_Kolksee |
| WP_048157956_1 | - | Uncharacterized protein, DUF1638 family | Methanosarcina_sp_Kolksee |
| WP_048157957_1 | AcrR | Transcriptional regulator, TetR/AcrR family | Methanosarcina_sp_Kolksee |
| WP_048157958_1 | #N/A | #N/A | Methanosarcina_sp_Kolksee |
| WP_048157959_1 | PylD | (3R)-3-methyl-D-ornithyl-N6-L-lysine dehydrogenase, pyrrolysine biosynthesis enzyme | Methanosarcina_sp_Kolksee |
| WP_048157960_1 | PylC | (3R)-3-methyl-D-ornithine:L-lysine ligase, pyrrolysine biosynthesis protein | Methanosarcina_sp_Kolksee |
| WP_048157961_1 | PylB/HcgA | (2R,3R)-3-methylornithine synthase involved in pyrrolysine biosynthesis/Iron-guanylylpyridinol (FeGP) cofactor biosynthesis protein HcgA | Methanosarcina_sp_Kolksee |
| WP_048157962_1 | PylS | Pyrrolysyl-tRNA-synthetase | Methanosarcina_sp_Kolksee |
| WP_048157963_1 | - | Uncharacterized Fe-S clusters-containing protein, contains DUF4445 domain | Methanosarcina_sp_Kolksee |
| WP_048123175_1 | #N/A | #N/A | Methanosarcina_sp_Kolksee |
| WP_048157966_1 | MtbA | Methylcobalamin:coenzyme M methyltransferase | Methanosarcina_sp_Kolksee |
| WP_082091188_1 | - | Pentapeptide repeats containing protein | Methanosarcina_sp_Kolksee |
| WP_048158455_1 | MtbC1 | Methanogenic corrinoid protein MtbC1 | Methanosarcina_sp_Kolksee |
| WP_048123179_1 | MtmB | Monomethylamine methyltransferase | Methanosarcina_sp_Kolksee |
| WP_082091189_1 | MtmB | Monomethylamine methyltransferase | Methanosarcina_sp_Kolksee |
| WP_048157967_1 | PotE | Amino acid transporter | Methanosarcina_sp_Kolksee |
| WP_048123186_1 | - | Uncharacterized membrane protein | Methanosarcina_sp_Kolksee |
| WP_048123188_1 | AcrB | Cation/multidrug efflux pump | Methanosarcina_sp_Kolksee |
| WP_048157968_1 | RFCS | Clamp loader ATPase, small subunit | Methanosarcina_sp_Kolksee |
| WP_048123191_1 | SecD | Preprotein translocase subunit SecD | Methanosarcina_sp_Kolksee |
| WP_048123193_1 | SecF | Preprotein translocase subunit SecF | Methanosarcina_sp_Kolksee |
| WP_048123195_1 | ComEB | Deoxycytidylate deaminase | Methanosarcina_sp_Kolksee |
| WP_048157969_1 | - | Cupin domain containing protein | Methanosarcina_sp_Kolksee |

| | | | |
|---|---|---|---|
| WP_048157970_1 | CofE | F(420)-0:gamma-glutamyl ligase, F420 coenzyme biosynthesis enzyme | Methanosarcina_sp_Kolksee |
| WP_048157971_1 | - | Uncharacterized protein | Methanosarcina_sp_Kolksee |
| | | | Methanosarcina_sp_WWM596 |
| WP_048129991_1 | - | Uncharacterized membrane protein | Methanosarcina_sp_WWM596 |
| WP_048129992_1 | - | Uncharacterized protein | Methanosarcina_sp_WWM596 |
| WP_048129993_1 | UspA | Nucleotide-binding protein, UspA family | Methanosarcina_sp_WWM596 |
| WP_048159482_1 | Ftn | Ferritin | Methanosarcina_sp_WWM596 |
| WP_048125153_1 | - | Transposase | Methanosarcina_sp_WWM596 |
| WP_048159483_1 | - | Homolog of Wybutosine (yW) biosynthesis enzyme, Fe-S oxidoreductase | Methanosarcina_sp_WWM596 |
| WP_048159484_1 | - | Uncharacterized protein | Methanosarcina_sp_WWM596 |
| WP_048129997_1 | - | Uncharacterized protein | Methanosarcina_sp_WWM596 |
| WP_048159485_1 | - | Predicted DNA-binding protein | Methanosarcina_sp_WWM596 |
| WP_048159487_1 | SqhC | Squalene cyclase | Methanosarcina_sp_WWM596 |
| WP_048130001_1 | PylS | Pyrrolysyl-tRNA-synthetase | Methanosarcina_sp_WWM596 |
| WP_048130002_1 | PylB/HcgA | (2R,3R)-3-methylornithine synthase involved in pyrrolysine biosynthesis/Iron-guanylylpyridinol (FeGP) cofactor biosynthesis protein HcgA | Methanosarcina_sp_WWM596 |
| WP_048130003_1 | PylC | (3R)-3-methyl-D-ornithine:L-lysine ligase, pyrrolysine biosynthesis protein | Methanosarcina_sp_WWM596 |
| WP_048130004_1 | PylD | (3R)-3-methyl-D-ornithyl-N6-L-lysine dehydrogenase, pyrrolysine biosynthesis enzyme | Methanosarcina_sp_WWM596 |
| WP_048130005_1 | - | Uncharacterized Fe-S clusters-containing protein, contains DUF4445 domain | Methanosarcina_sp_WWM596 |
| WP_048130006_1 | #N/A | #N/A | Methanosarcina_sp_WWM596 |
| WP_048130007_1 | MtbA | Methylcobalamin:coenzyme M methyltransferase | Methanosarcina_sp_WWM596 |
| WP_048130008_1 | MtbC1 | Methanogenic corrinoid protein MtbC1 | Methanosarcina_sp_WWM596 |
| WP_048126964_1 | MtmB | Monomethylamine methyltransferase | Methanosarcina_sp_WWM596 |
| WP_048159488_1 | MtmB | Monomethylamine methyltransferase | Methanosarcina_sp_WWM596 |
| WP_048130012_1 | PotE | Amino acid transporter | Methanosarcina_sp_WWM596 |
| WP_048130013_1 | - | Uncharacterized membrane protein | Methanosarcina_sp_WWM596 |
| WP_048130014_1 | AcrB | Cation/multidrug efflux pump | Methanosarcina_sp_WWM596 |
| WP_048159489_1 | RFCS | Clamp loader ATPase, small subunit | Methanosarcina_sp_WWM596 |
| WP_048130016_1 | SecD | Preprotein translocase subunit SecD | Methanosarcina_sp_WWM596 |
| WP_048130017_1 | SecF | Preprotein translocase subunit SecF | Methanosarcina_sp_WWM596 |
| WP_048130018_1 | ComEB | Deoxycytidylate deaminase | Methanosarcina_sp_WWM596 |
| WP_048130019_1 | CofE | F(420)-0:gamma-glutamyl ligase, F420 coenzyme biosynthesis enzyme | Methanosarcina_sp_WWM596 |
| WP_048130020_1 | - | Uncharacterized protein | Methanosarcina_sp_WWM596 |
| WP_048159490_1 | #N/A | #N/A | Methanosarcina_sp_WWM596 |

| | | | |
|---|---|---|---|
| | | | Methanosarcina_barkeri_str_Wiesmoor |
| WP_011305876_1 | SecF | Preprotein translocase subunit SecF | Methanosarcina_barkeri_str_Wiesmoor |
| WP_011305875_1 | SecD | Preprotein translocase subunit SecD | Methanosarcina_barkeri_str_Wiesmoor |
| WP_011305874_1 | RFCS | Clamp loader ATPase, small subunit | Methanosarcina_barkeri_str_Wiesmoor |
| WP_011305873_1 | AcrB | Cation/multidrug efflux pump | Methanosarcina_barkeri_str_Wiesmoor |
| WP_011305872_1 | - | Uncharacterized membrane protein | Methanosarcina_barkeri_str_Wiesmoor |
| WP_011305871_1 | PotE | Amino acid transporter | Methanosarcina_barkeri_str_Wiesmoor |
| WP_011305870_1 | MtbC1 | Methanogenic corrinoid protein MtbC1 | Methanosarcina_barkeri_str_Wiesmoor |
| WP_011305868_1 | MtbC1 | Methanogenic corrinoid protein MtbC1 | Methanosarcina_barkeri_str_Wiesmoor |
| WP_011305867_1 | MtbA | Methylcobalamin:coenzyme M methyltransferase | Methanosarcina_barkeri_str_Wiesmoor |
| WP_011305866_1 | - | Uncharacterized Fe-S clusters-containing protein, contains DUF4445 domain | Methanosarcina_barkeri_str_Wiesmoor |
| WP_011305865_1 | PylS | Pyrrolysyl-tRNA-synthetase | Methanosarcina_barkeri_str_Wiesmoor |
| WP_011305864_1 | PylB/HcgA | (2R,3R)-3-methylornithine synthase involved in pyrrolysine biosynthesis/Iron-guanylylpyridinol (FeGP) cofactor biosynthesis protein HcgA | Methanosarcina_barkeri_str_Wiesmoor |
| WP_048102615_1 | PylC | (3R)-3-methyl-D-ornithine:L-lysine ligase, pyrrolysine biosynthesis protein | Methanosarcina_barkeri_str_Wiesmoor |
| WP_048102612_1 | PylC | (3R)-3-methyl-D-ornithine:L-lysine ligase, pyrrolysine biosynthesis protein | Methanosarcina_barkeri_str_Wiesmoor |
| WP_048103181_1 | PylD | (3R)-3-methyl-D-ornithyl-N6-L-lysine dehydrogenase, pyrrolysine biosynthesis enzyme | Methanosarcina_barkeri_str_Wiesmoor |
| WP_011305861_1 | AcrR | Transcriptional regulator, TetR/AcrR family | Methanosarcina_barkeri_str_Wiesmoor |
| WP_011305860_1 | - | Uncharacterized protein, DUF1638 family | Methanosarcina_barkeri_str_Wiesmoor |
| WP_011305859_1 | - | Uncharacterized protein, DUF1638 family | Methanosarcina_barkeri_str_Wiesmoor |
| WP_048102607_1 | SqhC | Squalene cyclase | Methanosarcina_barkeri_str_Wiesmoor |
| WP_048102603_1 | - | Predicted DNA-binding protein | Methanosarcina_barkeri_str_Wiesmoor |
| WP_011305856_1 | - | Uncharacterized protein | Methanosarcina_barkeri_str_Wiesmoor |
| WP_011305855_1 | - | Homolog of Wybutosine (yW) biosynthesis enzyme, Fe-S oxidoreductase | Methanosarcina_barkeri_str_Wiesmoor |
| WP_048102601_1 | #N/A | #N/A | Methanosarcina_barkeri_str_Wiesmoor |
| WP_011305854_1 | Ftn | Ferritin | Methanosarcina_barkeri_str_Wiesmoor |
| WP_011305853_1 | UspA | Nucleotide-binding protein,  UspA family | Methanosarcina_barkeri_str_Wiesmoor |
| | | | Methanosarcina_sp_WH1 |
| WP_048129990_1 | - | Uncharacterized protein | Methanosarcina_sp_WH1 |
| WP_048129991_1 | - | Uncharacterized membrane protein | Methanosarcina_sp_WH1 |
| WP_048129992_1 | - | Uncharacterized protein | Methanosarcina_sp_WH1 |
| WP_048129993_1 | UspA | Nucleotide-binding protein,  UspA family | Methanosarcina_sp_WH1 |
| WP_048129994_1 | Ftn | Ferritin | Methanosarcina_sp_WH1 |
| WP_048129995_1 | - | Homolog of Wybutosine (yW) biosynthesis enzyme, Fe-S oxidoreductase | Methanosarcina_sp_WH1 |

| | | | |
|---|---|---|---|
| WP_048129996_1 | - | Uncharacterized protein | Methanosarcina_sp_WH1 |
| WP_048129997_1 | - | Uncharacterized protein | Methanosarcina_sp_WH1 |
| WP_048129998_1 | - | Predicted DNA-binding protein | Methanosarcina_sp_WH1 |
| WP_048130000_1 | SqhC | Squalene cyclase | Methanosarcina_sp_WH1 |
| WP_048130001_1 | PylS | Pyrrolysyl-tRNA-synthetase | Methanosarcina_sp_WH1 |
| WP_048130002_1 | PylB/HcgA | (2R,3R)-3-methylornithine synthase involved in pyrrolysine biosynthesis/Iron-guanylylpyridinol (FeGP) cofactor biosynthesis protein HcgA | Methanosarcina_sp_WH1 |
| WP_048130003_1 | PylC | (3R)-3-methyl-D-ornithine:L-lysine ligase, pyrrolysine biosynthesis protein | Methanosarcina_sp_WH1 |
| WP_048130004_1 | PylD | (3R)-3-methyl-D-ornithyl-N6-L-lysine dehydrogenase, pyrrolysine biosynthesis enzyme | Methanosarcina_sp_WH1 |
| WP_048130005_1 | - | Uncharacterized Fe-S clusters-containing protein, contains DUF4445 domain | Methanosarcina_sp_WH1 |
| WP_048130006_1 | #N/A | #N/A | Methanosarcina_sp_WH1 |
| WP_048130007_1 | MtbA | Methylcobalamin:coenzyme M methyltransferase | Methanosarcina_sp_WH1 |
| WP_048130008_1 | MtbC1 | Methanogenic corrinoid protein MtbC1 | Methanosarcina_sp_WH1 |
| WP_048126964_1 | MtmB | Monomethylamine methyltransferase | Methanosarcina_sp_WH1 |
| WP_048126962_1 | MtmB | Monomethylamine methyltransferase | Methanosarcina_sp_WH1 |
| WP_048130009_1 | MtbC1 | Methanogenic corrinoid protein MtbC1 | Methanosarcina_sp_WH1 |
| WP_048130010_1 | MtmB | Monomethylamine methyltransferase | Methanosarcina_sp_WH1 |
| WP_048130011_1 | MtmB | Monomethylamine methyltransferase | Methanosarcina_sp_WH1 |
| WP_048130012_1 | PotE | Amino acid transporter | Methanosarcina_sp_WH1 |
| WP_048130013_1 | - | Uncharacterized membrane protein | Methanosarcina_sp_WH1 |
| WP_048130014_1 | AcrB | Cation/multidrug efflux pump | Methanosarcina_sp_WH1 |
| WP_048130015_1 | RFCS | Clamp loader ATPase, small subunit | Methanosarcina_sp_WH1 |
| WP_048130016_1 | SecD | Preprotein translocase subunit SecD | Methanosarcina_sp_WH1 |
| WP_048130017_1 | SecF | Preprotein translocase subunit SecF | Methanosarcina_sp_WH1 |
| WP_048130018_1 | ComEB | Deoxycytidylate deaminase | Methanosarcina_sp_WH1 |
| WP_048130019_1 | CofE | F(420)-0:gamma-glutamyl ligase, F420 coenzyme biosynthesis enzyme | Methanosarcina_sp_WH1 |
| WP_048130020_1 | - | Uncharacterized protein | Methanosarcina_sp_WH1 |
| WP_048130021_1 | IbpA | Molecular chaperone (HSP20 family) | Methanosarcina_sp_WH1 |
| | | | Methanosarcina_sp_MTP4 |
| WP_048183944_1 | - | Uncharacterized protein | Methanosarcina_sp_MTP4 |
| WP_048181959_1 | UspA | Nucleotide-binding protein, UspA family | Methanosarcina_sp_MTP4 |
| WP_048181961_1 | UspA | Nucleotide-binding protein, UspA family | Methanosarcina_sp_MTP4 |
| WP_048181964_1 | Ftn | Ferritin | Methanosarcina_sp_MTP4 |
| WP_048181967_1 | #N/A | #N/A | Methanosarcina_sp_MTP4 |

| | | | |
|---|---|---|---|
| WP_048181969_1 | Ftn | Ferritin | Methanosarcina_sp_MTP4 |
| WP_048181973_1 | - | Homolog of Wybutosine (yW) biosynthesis enzyme, Fe-S oxidoreductase | Methanosarcina_sp_MTP4 |
| WP_082090674_1 | - | Uncharacterized protein | Methanosarcina_sp_MTP4 |
| WP_082090778_1 | - | Predicted DNA-binding protein | Methanosarcina_sp_MTP4 |
| WP_048181979_1 | - | Class II terpene cyclase family protein | Methanosarcina_sp_MTP4 |
| WP_048181983_1 | PylS | Pyrrolysyl-tRNA-synthetase | Methanosarcina_sp_MTP4 |
| WP_048183949_1 | PylB/HcgA | (2R,3R)-3-methylornithine synthase involved in pyrrolysine biosynthesis/Iron-guanylylpyridinol (FeGP) cofactor biosynthesis protein HcgA | Methanosarcina_sp_MTP4 |
| WP_082090675_1 | PylC | (3R)-3-methyl-D-ornithine:L-lysine ligase, pyrrolysine biosynthesis protein | Methanosarcina_sp_MTP4 |
| WP_048181985_1 | PylD | (3R)-3-methyl-D-ornithyl-N6-L-lysine dehydrogenase, pyrrolysine biosynthesis enzyme | Methanosarcina_sp_MTP4 |
| WP_048181988_1 | #N/A | #N/A | Methanosarcina_sp_MTP4 |
| WP_052718448_1 | - | Transcriptional regulator, contains HTH domain | Methanosarcina_sp_MTP4 |
| WP_048183957_1 | - | Formylglycine-generating sulfatase enzyme | Methanosarcina_sp_MTP4 |
| WP_048183960_1 | - | Uncharacterized Fe-S clusters-containing protein, contains DUF4445 domain | Methanosarcina_sp_MTP4 |
| WP_048181990_1 | MtbA | Methylcobalamin:coenzyme M methyltransferase | Methanosarcina_sp_MTP4 |
| WP_048181993_1 | MtbC1 | Methanogenic corrinoid protein MtbC1 | Methanosarcina_sp_MTP4 |
| WP_048178607_1 | MtmB | Monomethylamine methyltransferase | Methanosarcina_sp_MTP4 |
| WP_048178608_1 | MtmB | Monomethylamine methyltransferase | Methanosarcina_sp_MTP4 |
| WP_048181996_1 | PotE | Amino acid transporter | Methanosarcina_sp_MTP4 |
| WP_048183963_1 | - | Uncharacterized membrane protein | Methanosarcina_sp_MTP4 |
| WP_048183966_1 | AcrB | Cation/multidrug efflux pump | Methanosarcina_sp_MTP4 |
| WP_048181999_1 | RFCS | Clamp loader ATPase, small subunit | Methanosarcina_sp_MTP4 |
| WP_048182000_1 | SecD | Preprotein translocase subunit SecD | Methanosarcina_sp_MTP4 |
| WP_048182002_1 | SecF | Preprotein translocase subunit SecF | Methanosarcina_sp_MTP4 |
| WP_048182005_1 | ComEB | Deoxycytidylate deaminase | Methanosarcina_sp_MTP4 |
| WP_048182007_1 | CofE | F(420)-0:gamma-glutamyl ligase, F420 coenzyme biosynthesis enzyme | Methanosarcina_sp_MTP4 |
| WP_048182009_1 | - | Uncharacterized protein | Methanosarcina_sp_MTP4 |
| WP_048182012_1 | IbpA | Molecular chaperone (HSP20 family) | Methanosarcina_sp_MTP4 |
| | | | Methanosarcina_siciliae_C2J |
| WP_048169127_1 | IbpA | Molecular chaperone (HSP20 family) | Methanosarcina_siciliae_C2J |
| WP_048169129_1 | - | Uncharacterized protein | Methanosarcina_siciliae_C2J |
| WP_048178949_1 | CofE | F(420)-0:gamma-glutamyl ligase, F420 coenzyme biosynthesis enzyme | Methanosarcina_siciliae_C2J |
| WP_048178951_1 | ComEB | Deoxycytidylate deaminase | Methanosarcina_siciliae_C2J |
| WP_048169138_1 | SecF | Preprotein translocase subunit SecF | Methanosarcina_siciliae_C2J |

| | | | |
|---|---|---|---|
| WP_048178953_1 | SecD | Preprotein translocase subunit SecD | Methanosarcina_siciliae_C2J |
| WP_048169144_1 | RFCS | Clamp loader ATPase, small subunit | Methanosarcina_siciliae_C2J |
| WP_048169146_1 | AcrB | Cation/multidrug efflux pump | Methanosarcina_siciliae_C2J |
| WP_048169148_1 | - | Uncharacterized membrane protein | Methanosarcina_siciliae_C2J |
| WP_048178955_1 | PotE | Amino acid transporter | Methanosarcina_siciliae_C2J |
| WP_082092883_1 | MtmB | Monomethylamine methyltransferase | Methanosarcina_siciliae_C2J |
| WP_048169156_1 | MtbC1 | Methanogenic corrinoid protein MtbC1 | Methanosarcina_siciliae_C2J |
| WP_048178960_1 | #N/A | #N/A | Methanosarcina_siciliae_C2J |
| WP_048178962_1 | #N/A | #N/A | Methanosarcina_siciliae_C2J |
| WP_048178965_1 | #N/A | #N/A | Methanosarcina_siciliae_C2J |
| WP_048178969_1 | MtbC1 | Methanogenic corrinoid protein MtbC1 | Methanosarcina_siciliae_C2J |
| WP_048169167_1 | MtbA | Methylcobalamin:coenzyme M methyltransferase | Methanosarcina_siciliae_C2J |
| WP_048178972_1 | MdaB | NADPH-quinone reductase (modulator of drug activity B) | Methanosarcina_siciliae_C2J |
| WP_048178975_1 | #N/A | #N/A | Methanosarcina_siciliae_C2J |
| WP_048169173_1 | - | Uncharacterized Fe-S clusters-containing protein, contains DUF4445 domain | Methanosarcina_siciliae_C2J |
| WP_052727138_1 | WecB | UDP-N-acetylglucosamine 2-epimerase | Methanosarcina_siciliae_C2J |
| WP_082088829_1 | WecB | UDP-N-acetylglucosamine 2-epimerase | Methanosarcina_siciliae_C2J |
| WP_048178978_1 | CDA1 | Peptidoglycan/xylan/chitin deacetylase, PgdA/CDA1 family | Methanosarcina_siciliae_C2J |
| WP_048178980_1 | PylD | (3R)-3-methyl-D-ornithyl-N6-L-lysine dehydrogenase, pyrrolysine biosynthesis enzyme | Methanosarcina_siciliae_C2J |
| WP_048185492_1 | PylC | (3R)-3-methyl-D-ornithine:L-lysine ligase, pyrrolysine biosynthesis protein | Methanosarcina_siciliae_C2J |
| WP_048178982_1 | PylB/HcgA | (2R,3R)-3-methylornithine synthase involved in pyrrolysine biosynthesis/Iron-guanylylpyridinol (FeGP) cofactor biosynthesis protein HcgA | Methanosarcina_siciliae_C2J |
| WP_048178985_1 | PylS | Pyrrolysyl-tRNA-synthetase | Methanosarcina_siciliae_C2J |
| WP_048178986_1 | #N/A | #N/A | Methanosarcina_siciliae_C2J |
| WP_048178988_1 | - | Class II terpene cyclase family protein | Methanosarcina_siciliae_C2J |
| WP_048185494_1 | - | Predicted DNA-binding protein | Methanosarcina_siciliae_C2J |
| WP_082088830_1 | - | Uncharacterized protein | Methanosarcina_siciliae_C2J |
| WP_048169186_1 | - | Predicted DNA-binding protein | Methanosarcina_siciliae_C2J |
| WP_048185496_1 | - | Homolog of Wybutosine (yW) biosynthesis enzyme, Fe-S oxidoreductase | Methanosarcina_siciliae_C2J |
| WP_048169188_1 | Ftn | Ferritin | Methanosarcina_siciliae_C2J |
| WP_048178991_1 | UspA | Nucleotide-binding protein,  UspA family | Methanosarcina_siciliae_C2J |
| WP_048178993_1 | - | Uncharacterized protein | Methanosarcina_siciliae_C2J |
| WP_048178995_1 | - | Uncharacterized membrane protein | Methanosarcina_siciliae_C2J |
| | | | Methanosarcina_lacustris_Z-7289 |

| | | | |
|---|---|---|---|
| WP_048128716_1 | SecF | Preprotein translocase subunit SecF | Methanosarcina_lacustris_Z-7289 |
| WP_048128717_1 | SecD | Preprotein translocase subunit SecD | Methanosarcina_lacustris_Z-7289 |
| WP_048128718_1 | RFCS | Clamp loader ATPase, small subunit | Methanosarcina_lacustris_Z-7289 |
| WP_048128719_1 | AcrB | Cation/multidrug efflux pump | Methanosarcina_lacustris_Z-7289 |
| WP_048128721_1 | - | Uncharacterized membrane protein | Methanosarcina_lacustris_Z-7289 |
| WP_048128723_1 | PotE | Amino acid transporter | Methanosarcina_lacustris_Z-7289 |
| WP_048128728_1 | MtbC1 | Methanogenic corrinoid protein MtbC1 | Methanosarcina_lacustris_Z-7289 |
| WP_048128733_1 | MtbC1 | Methanogenic corrinoid protein MtbC1 | Methanosarcina_lacustris_Z-7289 |
| WP_048128735_1 | MtbA | Methylcobalamin:coenzyme M methyltransferase | Methanosarcina_lacustris_Z-7289 |
| WP_048128737_1 | - | Uncharacterized Fe-S clusters-containing protein, contains DUF4445 domain | Methanosarcina_lacustris_Z-7289 |
| WP_048128739_1 | PylD | (3R)-3-methyl-D-ornithyl-N6-L-lysine dehydrogenase, pyrrolysine biosynthesis enzyme | Methanosarcina_lacustris_Z-7289 |
| WP_048128740_1 | PylC | (3R)-3-methyl-D-ornithine:L-lysine ligase, pyrrolysine biosynthesis protein | Methanosarcina_lacustris_Z-7289 |
| WP_048128741_1 | PylB/HcgA | (2R,3R)-3-methylornithine synthase involved in pyrrolysine biosynthesis/Iron-guanylylpyridinol (FeGP) cofactor biosynthesis protein HcgA | Methanosarcina_lacustris_Z-7289 |
| WP_048128743_1 | PylS | Pyrrolysyl-tRNA-synthetase | Methanosarcina_lacustris_Z-7289 |
| WP_048128744_1 | #N/A | #N/A | Methanosarcina_lacustris_Z-7289 |
| WP_084630857_1 | - | Predicted DNA-binding protein | Methanosarcina_lacustris_Z-7289 |
| WP_084630860_1 | #N/A | #N/A | Methanosarcina_lacustris_Z-7289 |
| WP_048128752_1 | - | Uncharacterized protein | Methanosarcina_lacustris_Z-7289 |
| WP_048128754_1 | - | Homolog of Wybutosine (yW) biosynthesis enzyme, Fe-S oxidoreductase | Methanosarcina_lacustris_Z-7289 |
| WP_084630863_1 | - | Cytochrome b family protein | Methanosarcina_lacustris_Z-7289 |
| WP_048128759_1 | - | Pheromone shutdown protein TraB, contains GTxH motif | Methanosarcina_lacustris_Z-7289 |
| WP_048128763_1 | - | Zn-dependent protease fused to CBS domain | Methanosarcina_lacustris_Z-7289 |
| WP_048128765_1 | MfnA | L-tyrosine decarboxylase, PLP-dependent protein | Methanosarcina_lacustris_Z-7289 |
| WP_048128767_1 | #N/A | #N/A | Methanosarcina_lacustris_Z-7289 |
| | | | Methanosarcina_horonobensis_HB-1_JCM_15518 |
| WP_082089448_1 | - | Uncharacterized protein | Methanosarcina_horonobensis_HB-1_JCM_15518 |
| WP_048142676_1 | - | Uncharacterized protein, DUF2892 family | Methanosarcina_horonobensis_HB-1_JCM_15518 |
| WP_048142678_1 | YcaO | Ribosomal protein S12 methylthiotransferase accessory factor YcaO | Methanosarcina_horonobensis_HB-1_JCM_15518 |
| WP_048142680_1 | - | Uncharacterized protein | Methanosarcina_horonobensis_HB-1_JCM_15518 |
| WP_048142682_1 | - | Uncharacterized membrane protein | Methanosarcina_horonobensis_HB-1_JCM_15518 |
| WP_048142683_1 | - | Uncharacterized protein | Methanosarcina_horonobensis_HB-1_JCM_15518 |
| WP_048142685_1 | #N/A | #N/A | Methanosarcina_horonobensis_HB-1_JCM_15518 |

| | | | |
|---|---|---|---|
| WP_048142687_1 | - | Predicted DNA-binding protein | Methanosarcina_horonobensis_HB-1_JCM_15518 |
| WP_048142690_1 | #N/A | #N/A | Methanosarcina_horonobensis_HB-1_JCM_15518 |
| WP_048142692_1 | #N/A | #N/A | Methanosarcina_horonobensis_HB-1_JCM_15518 |
| WP_048142694_1 | PylS | Pyrrolysyl-tRNA-synthetase | Methanosarcina_horonobensis_HB-1_JCM_15518 |
| WP_048142696_1 | PylB/HcgA | (2R,3R)-3-methylornithine synthase involved in pyrrolysine biosynthesis/Iron-guanylylpyridinol (FeGP) cofactor biosynthesis protein HcgA | Methanosarcina_horonobensis_HB-1_JCM_15518 |
| WP_048142698_1 | PylC | (3R)-3-methyl-D-ornithine:L-lysine ligase, pyrrolysine biosynthesis protein | Methanosarcina_horonobensis_HB-1_JCM_15518 |
| WP_048142700_1 | PylD | (3R)-3-methyl-D-ornithyl-N6-L-lysine dehydrogenase, pyrrolysine biosynthesis enzyme | Methanosarcina_horonobensis_HB-1_JCM_15518 |
| WP_048142702_1 | Ppa | Inorganic pyrophosphatase | Methanosarcina_horonobensis_HB-1_JCM_15518 |
| WP_048142704_1 | - | Uncharacterized Fe-S clusters-containing protein, contains DUF4445 domain | Methanosarcina_horonobensis_HB-1_JCM_15518 |
| WP_048142706_1 | - | SAM-dependent methyltransferase | Methanosarcina_horonobensis_HB-1_JCM_15518 |
| WP_082089449_1 | MdaB | NADPH-quinone reductase (modulator of drug activity B) | Methanosarcina_horonobensis_HB-1_JCM_15518 |
| WP_048142710_1 | MtbA | Methylcobalamin:coenzyme M methyltransferase | Methanosarcina_horonobensis_HB-1_JCM_15518 |
| WP_048142712_1 | MtbC1 | Methanogenic corrinoid protein MtbC1 | Methanosarcina_horonobensis_HB-1_JCM_15518 |
| WP_048142714_1 | MtmB | Monomethylamine methyltransferase | Methanosarcina_horonobensis_HB-1_JCM_15518 |
| WP_048142716_1 | MtmB | Monomethylamine methyltransferase | Methanosarcina_horonobensis_HB-1_JCM_15518 |
| WP_048142718_1 | PotE | Amino acid transporter | Methanosarcina_horonobensis_HB-1_JCM_15518 |
| WP_048142720_1 | - | Uncharacterized membrane protein | Methanosarcina_horonobensis_HB-1_JCM_15518 |
| WP_048142722_1 | AcrB | Cation/multidrug efflux pump | Methanosarcina_horonobensis_HB-1_JCM_15518 |
| WP_048142724_1 | #N/A | #N/A | Methanosarcina_horonobensis_HB-1_JCM_15518 |
| WP_048142725_1 | #N/A | #N/A | Methanosarcina_horonobensis_HB-1_JCM_15518 |
| WP_052730957_1 | AprE | Predicted multidomain toxin, contains peptidase S8 domain, delivery module and Rnase A domain | Methanosarcina_horonobensis_HB-1_JCM_15518 |
| WP_048142727_1 | RFCS | Clamp loader ATPase, small subunit | Methanosarcina_horonobensis_HB-1_JCM_15518 |
| WP_048142729_1 | SecD | Preprotein translocase subunit SecD | Methanosarcina_horonobensis_HB-1_JCM_15518 |
| WP_048142730_1 | SecF | Preprotein translocase subunit SecF | Methanosarcina_horonobensis_HB-1_JCM_15518 |
| WP_048142732_1 | ComEB | Deoxycytidylate deaminase | Methanosarcina_horonobensis_HB-1_JCM_15518 |
| | | | Methanococcoides_methylutens_MM1 |
| WP_048204546_1 | NPY1 | NTP pyrophosphohydrolase containing a Zn-finger, probably nucleic-acid-binding | Methanococcoides_methylutens_MM1 |
| WP_082087352_1 | MgtC | Mg2+ transport system protein | Methanococcoides_methylutens_MM1 |
| WP_048204547_1 | - | Rhodanese Homology Domain fused to Zn-dependent hydrolase of beta-lactamase superfamily | Methanococcoides_methylutens_MM1 |
| WP_048204548_1 | - | Predicted Fe-Mo cluster-binding protein, NifX family | Methanococcoides_methylutens_MM1 |
| WP_048204549_1 | - | Metal-dependent hydrolase of the beta-lactamase superfamily II | Methanococcoides_methylutens_MM1 |
| WP_048204550_1 | - | Uncharacterized metal-binding protein, DUF1847 family | Methanococcoides_methylutens_MM1 |

| | | | |
|---|---|---|---|
| WP_048204551_1 | - | MinD superfamily P-loop ATPase containing an inserted ferredoxin domain | Methanococcoides_methylutens_MM1 |
| WP_048204552_1 | - | MinD superfamily P-loop ATPase containing an inserted ferredoxin domain | Methanococcoides_methylutens_MM1 |
| WP_048204553_1 | - | Predicted Fe-Mo cluster-binding protein, NifX family | Methanococcoides_methylutens_MM1 |
| WP_048204554_1 | MtbA | Methylcobalamin:coenzyme M methyltransferase | Methanococcoides_methylutens_MM1 |
| WP_048204555_1 | PylD | (3R)-3-methyl-D-ornithyl-N6-L-lysine dehydrogenase, pyrrolysine biosynthesis enzyme | Methanococcoides_methylutens_MM1 |
| WP_048204556_1 | PylC | (3R)-3-methyl-D-ornithine:L-lysine ligase, pyrrolysine biosynthesis protein | Methanococcoides_methylutens_MM1 |
| WP_048204557_1 | PylB/HcgA | (2R,3R)-3-methylornithine synthase involved in pyrrolysine biosynthesis/Iron-guanylylpyridinol (FeGP) cofactor biosynthesis protein HcgA | Methanococcoides_methylutens_MM1 |
| WP_048204558_1 | PylS | Pyrrolysyl-tRNA-synthetase | Methanococcoides_methylutens_MM1 |
| WP_048206293_1 | HisC | Histidinol-phosphate/aromatic aminotransferase or cobyric acid decarboxylase | Methanococcoides_methylutens_MM1 |
| WP_048204559_1 | - | GTP:adenosylcobinamide-phosphate guanylyltransferase | Methanococcoides_methylutens_MM1 |
| WP_048204560_1 | HisC | Histidinol-phosphate/aromatic aminotransferase or cobyric acid decarboxylase | Methanococcoides_methylutens_MM1 |
| WP_048206294_1 | CbiB | Cobalamin biosynthesis protein CobD/CbiB | Methanococcoides_methylutens_MM1 |
| WP_048204561_1 | PgpA | Phosphatidylglycerophosphatase A fused to adenosylcobinamide amidohydrolase, CbiZ | Methanococcoides_methylutens_MM1 |
| WP_048204562_1 | CobS | Cobalamin-5-phosphate synthase | Methanococcoides_methylutens_MM1 |
| WP_048204563_1 | - | GTP:adenosylcobinamide-phosphate guanylyltransferase | Methanococcoides_methylutens_MM1 |
| WP_048204564_1 | - | Uncharacterized protein | Methanococcoides_methylutens_MM1 |
| WP_048204565_1 | KaiC | KaiC family ATPase implicated in signal transduction | Methanococcoides_methylutens_MM1 |
| WP_048204566_1 | - | Queuine tRNA-ribosyltransferase related protein | Methanococcoides_methylutens_MM1 |
| | | | methanogenic_archaeon_ISO4-H5 |
| WP_066075732_1 | - | DNA repair photolyase | methanogenic_archaeon_ISO4-H5 |
| WP_066075735_1 | - | Predicted Na+-dependent transporter | methanogenic_archaeon_ISO4-H5 |
| WP_066075739_1 | - | Cro/C1-type HTH DNA-binding protein | methanogenic_archaeon_ISO4-H5 |
| WP_066075742_1 | #N/A | #N/A | methanogenic_archaeon_ISO4-H5 |
| WP_066075745_1 | MenA | 1,4-dihydroxy-2-naphthoate octaprenyltransferase | methanogenic_archaeon_ISO4-H5 |
| WP_066075752_1 | UbiE | Ubiquinone/menaquinone biosynthesis C-methylase UbiE | methanogenic_archaeon_ISO4-H5 |
| WP_066075755_1 | FrvX | Peptidase M42 family protein | methanogenic_archaeon_ISO4-H5 |
| WP_066075758_1 | #N/A | #N/A | methanogenic_archaeon_ISO4-H5 |
| WP_066075761_1 | - | Uncharacterized Fe-S clusters-containing protein, contains DUF4445 domain | methanogenic_archaeon_ISO4-H5 |
| WP_066075764_1 | SrmB | Superfamily II DNA and RNA helicase | methanogenic_archaeon_ISO4-H5 |
| WP_066075767_1 | PylD | (3R)-3-methyl-D-ornithyl-N6-L-lysine dehydrogenase, pyrrolysine biosynthesis enzyme | methanogenic_archaeon_ISO4-H5 |
| WP_066077272_1 | PylC | (3R)-3-methyl-D-ornithine:L-lysine ligase, pyrrolysine biosynthesis protein | methanogenic_archaeon_ISO4-H5 |

| | | | |
|---|---|---|---|
| WP_066075770_1 | PylB/HcgA | (2R,3R)-3-methylornithine synthase involved in pyrrolysine biosynthesis/Iron-guanylylpyridinol (FeGP) cofactor biosynthesis protein HcgA | methanogenic_archaeon_ISO4-H5 |
| WP_066075773_1 | PylS | Pyrrolysyl-tRNA-synthetase | methanogenic_archaeon_ISO4-H5 |
| WP_066077279_1 | MtbC1 | Methanogenic corrinoid protein MtbC1 | methanogenic_archaeon_ISO4-H5 |
| WP_082730147_1 | MtbC1 | Methanogenic corrinoid protein MtbC1 | methanogenic_archaeon_ISO4-H5 |
| WP_066075784_1 | PotE | Amino acid transporter | methanogenic_archaeon_ISO4-H5 |
| WP_066077287_1 | MtbC1 | Methanogenic corrinoid protein MtbC1 | methanogenic_archaeon_ISO4-H5 |
| WP_066075790_1 | - | Zn-ribbons containing membrane protein | methanogenic_archaeon_ISO4-H5 |
| WP_066075793_1 | - | Uncharacterized membrane protein, DUF1846 family | methanogenic_archaeon_ISO4-H5 |
| WP_066075795_1 | EbsC | Cys-tRNA(Pro)/Cys-tRNA(Cys) deacylase, ybaK family | methanogenic_archaeon_ISO4-H5 |
| WP_066075800_1 | SrmB | Superfamily II DNA and RNA helicase | methanogenic_archaeon_ISO4-H5 |
| WP_066075804_1 | WrbA | Multimeric flavodoxin WrbA | methanogenic_archaeon_ISO4-H5 |
| WP_066075807_1 | ValS | Valyl-tRNA synthetase | methanogenic_archaeon_ISO4-H5 |
| | | | Methanohalophilus_halophilus_Z-7982 |
| WP_072562258_1 | Rnz | Ribonuclease Z, beta-lactamase superfamily hydrolase | Methanohalophilus_halophilus_Z-7982 |
| WP_072560740_1 | RPS8A | Ribosomal protein S8E | Methanohalophilus_halophilus_Z-7982 |
| WP_072560742_1 | - | DNA-binding transcriptional regulator, Lrp family | Methanohalophilus_halophilus_Z-7982 |
| WP_072560744_1 | - | Aspartate/tyrosine/aromatic aminotransferase | Methanohalophilus_halophilus_Z-7982 |
| WP_072560746_1 | LabA | NYN domain, predicted PIN-related RNAse, tRNA/rRNA maturation | Methanohalophilus_halophilus_Z-7982 |
| WP_072560747_1 | - | MFS family permease | Methanohalophilus_halophilus_Z-7982 |
| WP_072560749_1 | Sta1 | Viral transcriptional activator, contains HTH domain | Methanohalophilus_halophilus_Z-7982 |
| WP_072560751_1 | - | Queuine tRNA-ribosyltransferase related protein | Methanohalophilus_halophilus_Z-7982 |
| WP_083432991_1 | - | Signal transduction histidine kinase, contains REC and PAS domains | Methanohalophilus_halophilus_Z-7982 |
| WP_072560753_1 | HisC | Histidinol-phosphate/aromatic aminotransferase or cobyric acid decarboxylase | Methanohalophilus_halophilus_Z-7982 |
| WP_072560754_1 | PylS | Pyrrolysyl-tRNA-synthetase | Methanohalophilus_halophilus_Z-7982 |
| WP_072560756_1 | PylB/HcgA | (2R,3R)-3-methylornithine synthase involved in pyrrolysine biosynthesis/Iron-guanylylpyridinol (FeGP) cofactor biosynthesis protein HcgA | Methanohalophilus_halophilus_Z-7982 |
| WP_072560758_1 | PylC | (3R)-3-methyl-D-ornithine:L-lysine ligase, pyrrolysine biosynthesis protein | Methanohalophilus_halophilus_Z-7982 |
| WP_072560760_1 | PylD | (3R)-3-methyl-D-ornithyl-N6-L-lysine dehydrogenase, pyrrolysine biosynthesis enzyme | Methanohalophilus_halophilus_Z-7982 |
| WP_072560761_1 | MtbA | Methylcobalamin:coenzyme M methyltransferase | Methanohalophilus_halophilus_Z-7982 |
| WP_072560763_1 | - | Predicted Fe-Mo cluster-binding protein, NifX family | Methanohalophilus_halophilus_Z-7982 |
| WP_072560765_1 | - | Predicted Fe-Mo cluster-binding protein, NifX family | Methanohalophilus_halophilus_Z-7982 |
| WP_072560767_1 | FrhB | Coenzyme F420-reducing hydrogenase, beta subunit fused to dissimilatory sulfite reductase | Methanohalophilus_halophilus_Z-7982 |

| | | | |
|---|---|---|---|
| WP_072560769_1 | - | MinD superfamily P-loop ATPase containing an inserted ferredoxin domain | Methanohalophilus_halophilus_Z-7982 |
| WP_072560771_1 | - | MinD superfamily P-loop ATPase containing an inserted ferredoxin domain | Methanohalophilus_halophilus_Z-7982 |
| WP_072560773_1 | - | Uncharacterized metal-binding protein, DUF1847 family | Methanohalophilus_halophilus_Z-7982 |
| WP_072560775_1 | - | Metal-dependent hydrolase of the beta-lactamase superfamily II | Methanohalophilus_halophilus_Z-7982 |
| WP_072560777_1 | - | Ferritin-like domain | Methanohalophilus_halophilus_Z-7982 |
| WP_072560778_1 | MgtC | Mg2+ transport system protein | Methanohalophilus_halophilus_Z-7982 |
| | | | Methanomassiliicoccus_luminyensis_B10 |
| WP_019176296_1 | MarR | Transcriptional regulator, MarR family | Methanomassiliicoccus_luminyensis_B10 |
| WP_019176297_1 | - | MFS family permease | Methanomassiliicoccus_luminyensis_B10 |
| WP_049796211_1 | #N/A | #N/A | Methanomassiliicoccus_luminyensis_B10 |
| WP_019176299_1 | - | Uncharacterized protein, DUF1638 family | Methanomassiliicoccus_luminyensis_B10 |
| WP_081579755_1 | ArsR | Transcriptional regulator containing HTH domain, ArsR family | Methanomassiliicoccus_luminyensis_B10 |
| WP_019176301_1 | Cdc48 | ATPase of the AAA+ class , CDC48 family | Methanomassiliicoccus_luminyensis_B10 |
| WP_019176302_1 | - | Predicted dinucleotide-binding enzyme | Methanomassiliicoccus_luminyensis_B10 |
| WP_019176303_1 | - | Predicted surface protease of transglutaminase family | Methanomassiliicoccus_luminyensis_B10 |
| WP_019176304_1 | AmtB | Ammonia permease | Methanomassiliicoccus_luminyensis_B10 |
| WP_026068678_1 | MtbC1 | Methanogenic corrinoid protein MtbC1 | Methanomassiliicoccus_luminyensis_B10 |
| WP_019176308_1 | PylS | Pyrrolysyl-tRNA-synthetase | Methanomassiliicoccus_luminyensis_B10 |
| WP_019176309_1 | PylB/HcgA | (2R,3R)-3-methylornithine synthase involved in pyrrolysine biosynthesis/Iron-guanylylpyridinol (FeGP) cofactor biosynthesis protein HcgA | Methanomassiliicoccus_luminyensis_B10 |
| WP_019176310_1 | PylC | (3R)-3-methyl-D-ornithine:L-lysine ligase, pyrrolysine biosynthesis protein | Methanomassiliicoccus_luminyensis_B10 |
| WP_081579756_1 | PylD | (3R)-3-methyl-D-ornithyl-N6-L-lysine dehydrogenase, pyrrolysine biosynthesis enzyme | Methanomassiliicoccus_luminyensis_B10 |
| WP_019176312_1 | - | Uncharacterized Fe-S clusters-containing protein, contains DUF4445 domain | Methanomassiliicoccus_luminyensis_B10 |
| WP_026068679_1 | MtbC1 | Methanogenic corrinoid protein MtbC1 | Methanomassiliicoccus_luminyensis_B10 |
| WP_019176314_1 | MtmB | Monomethylamine methyltransferase | Methanomassiliicoccus_luminyensis_B10 |
| WP_081579757_1 | MtmB | Monomethylamine methyltransferase | Methanomassiliicoccus_luminyensis_B10 |
| WP_019176316_1 | ArsR | Transcriptional regulator containing HTH domain, ArsR family | Methanomassiliicoccus_luminyensis_B10 |
| WP_081579765_1 | - | Predicted flavin-nucleotide-binding protein structurally related to pyridoxine 5'-phosphate oxidase | Methanomassiliicoccus_luminyensis_B10 |
| WP_019176318_1 | IbpA | Molecular chaperone (HSP20 family) | Methanomassiliicoccus_luminyensis_B10 |
| WP_019176319_1 | - | Membrane associated inactivated KaiC-like ATPase, DUF835 family | Methanomassiliicoccus_luminyensis_B10 |
| WP_019176321_1 | AcrR | Transcriptional regulator, TetR/AcrR family | Methanomassiliicoccus_luminyensis_B10 |
| WP_081579758_1 | - | Surface protein containing fasciclin-like repeats | Methanomassiliicoccus_luminyensis_B10 |
| WP_019176323_1 | TrxA | Thiol-disulfide isomerase or thioredoxin | Methanomassiliicoccus_luminyensis_B10 |
| WP_026068681_1 | YpwA | Zn-dependent carboxypeptidase, M32 family | Methanomassiliicoccus_luminyensis_B10 |

| | | | |
|---|---|---|---|
| WP_019176325_1 | TrkA | TrkA, K+ transport system, NAD-binding component | Methanomassiliicoccus_luminyensis_B10 |
| WP_049796203_1 | TrkG | Trk-type K+ transport system, membrane component | Methanomassiliicoccus_luminyensis_B10 |
| | | | Methanomassiliicoccus_luminyensis_B10 |
| WP_019178507_1 | #N/A | #N/A | Methanomassiliicoccus_luminyensis_B10 |
| WP_019178508_1 | QueG | Epoxyqueuosine reductase QueG (queuosine biosynthesis) | Methanomassiliicoccus_luminyensis_B10 |
| WP_019178509_1 | Lhr | Lhr-like helicase with C-terminal Zn finger domain | Methanomassiliicoccus_luminyensis_B10 |
| WP_019178510_1 | ARA1 | Aldo/keto reductase, related to diketogulonate reductase | Methanomassiliicoccus_luminyensis_B10 |
| WP_026069096_1 | SatP | Succinate-acetate transporter protein | Methanomassiliicoccus_luminyensis_B10 |
| WP_019178512_1 | #N/A | #N/A | Methanomassiliicoccus_luminyensis_B10 |
| WP_049796367_1 | #N/A | #N/A | Methanomassiliicoccus_luminyensis_B10 |
| WP_019178514_1 | UbiA | 4-hydroxybenzoate polyprenyltransferase or related prenyltransferase | Methanomassiliicoccus_luminyensis_B10 |
| WP_019178515_1 | - | Uncharacterized protein, DUF1638 family | Methanomassiliicoccus_luminyensis_B10 |
| WP_019178518_1 | MtbC1 | Methanogenic corrinoid protein MtbC1 | Methanomassiliicoccus_luminyensis_B10 |
| WP_026069099_1 | MttB2 | Trimethylamine:corrinoid methyltransferase | Methanomassiliicoccus_luminyensis_B10 |
| WP_081579989_1 | MttB1 | Trimethylamine:corrinoid methyltransferase | Methanomassiliicoccus_luminyensis_B10 |
| WP_026069100_1 | MtbC1 | Methanogenic corrinoid protein MtbC1 | Methanomassiliicoccus_luminyensis_B10 |
| WP_049796368_1 | RhaT | Permease of the drug/metabolite transporter (DMT) superfamily | Methanomassiliicoccus_luminyensis_B10 |
| WP_019178523_1 | - | Uncharacterized protein | Methanomassiliicoccus_luminyensis_B10 |
| WP_026069102_1 | MtbC1 | Methanogenic corrinoid protein MtbC1 | Methanomassiliicoccus_luminyensis_B10 |
| WP_026069103_1 | MtbB | Dimethylamine methyltransferase | Methanomassiliicoccus_luminyensis_B10 |
| WP_019178527_1 | MtbB | Dimethylamine methyltransferase | Methanomassiliicoccus_luminyensis_B10 |
| WP_081579990_1 | PotE | Amino acid transporter | Methanomassiliicoccus_luminyensis_B10 |
| WP_019178529_1 | PylS | Pyrrolysyl-tRNA-synthetase | Methanomassiliicoccus_luminyensis_B10 |
| WP_019178530_1 | PylB/HcgA | (2R,3R)-3-methylornithine synthase involved in pyrrolysine biosynthesis/Iron-guanylylpyridinol (FeGP) cofactor biosynthesis protein HcgA | Methanomassiliicoccus_luminyensis_B10 |
| WP_019178531_1 | PylC | (3R)-3-methyl-D-ornithine:L-lysine ligase, pyrrolysine biosynthesis protein | Methanomassiliicoccus_luminyensis_B10 |
| WP_019178532_1 | PylD | (3R)-3-methyl-D-ornithyl-N6-L-lysine dehydrogenase, pyrrolysine biosynthesis enzyme | Methanomassiliicoccus_luminyensis_B10 |
| WP_019178534_1 | - | Transcriptional regulator, contains N-terminal RHH domain | Methanomassiliicoccus_luminyensis_B10 |
| WP_019178535_1 | - | PAS domain | Methanomassiliicoccus_luminyensis_B10 |
| WP_019178536_1 | IbpA | Molecular chaperone (HSP20 family) | Methanomassiliicoccus_luminyensis_B10 |
| WP_019178537_1 | ACR3 | Arsenite efflux pump ACR3 or related permease | Methanomassiliicoccus_luminyensis_B10 |
| WP_019178538_1 | TspO | Tryptophan-rich sensory protein | Methanomassiliicoccus_luminyensis_B10 |
| WP_026069107_1 | #N/A | #N/A | Methanomassiliicoccus_luminyensis_B10 |
| WP_019178540_1 | HutI | Imidazolonepropionase or related amidohydrolase | Methanomassiliicoccus_luminyensis_B10 |

| | | | |
|---|---|---|---|
| WP_019178541_1 | DedA | Uncharacterized membrane-associated protein, DedA family | Methanomassiliicoccus_luminyensis_B10 |
| WP_019178542_1 | - | Uncharacterized membrane protein | Methanomassiliicoccus_luminyensis_B10 |
| WP_019178543_1 | ArsR | Transcriptional regulator containing HTH domain, ArsR family | Methanomassiliicoccus_luminyensis_B10 |
| | | | Methanolobus_tindarius_DSM_2278 |
| WP_023845203_1 | - | Zn-dependent protease fused to CBS domain | Methanolobus_tindarius_DSM_2278 |
| WP_023845204_1 | MfnA | L-tyrosine decarboxylase, PLP-dependent protein | Methanolobus_tindarius_DSM_2278 |
| WP_023845205_1 | KaiC | KaiC family ATPase implicated in signal transduction | Methanolobus_tindarius_DSM_2278 |
| WP_023845206_1 | CheF | Component of chemotaxis system associated with archaellum, contains CheF-like and HTH domain | Methanolobus_tindarius_DSM_2278 |
| WP_048135275_1 | - | PAS domain | Methanolobus_tindarius_DSM_2278 |
| WP_023845207_1 | CDC6 | Cdc6-related protein, AAA superfamily ATPase | Methanolobus_tindarius_DSM_2278 |
| WP_023845208_1 | LepB | Signal peptidase I | Methanolobus_tindarius_DSM_2278 |
| WP_023845209_1 | HYS2 | Archaeal DNA polymerase II, small subunit/DNA polymerase delta, subunit B | Methanolobus_tindarius_DSM_2278 |
| WP_048135277_1 | - | Predicted restriction endonuclease, Mrr-cat superfamily | Methanolobus_tindarius_DSM_2278 |
| WP_023845210_1 | - | Uncharacterized Fe-S clusters-containing protein, contains DUF4445 domain | Methanolobus_tindarius_DSM_2278 |
| WP_023845211_1 | PylD | (3R)-3-methyl-D-ornithyl-N6-L-lysine dehydrogenase, pyrrolysine biosynthesis enzyme | Methanolobus_tindarius_DSM_2278 |
| WP_023845212_1 | PylC | (3R)-3-methyl-D-ornithine:L-lysine ligase, pyrrolysine biosynthesis protein | Methanolobus_tindarius_DSM_2278 |
| WP_023845213_1 | PylB/HcgA | (2R,3R)-3-methylornithine synthase involved in pyrrolysine biosynthesis/Iron-guanylylpyridinol (FeGP) cofactor biosynthesis protein HcgA | Methanolobus_tindarius_DSM_2278 |
| WP_023845214_1 | PylS | Pyrrolysyl-tRNA-synthetase | Methanolobus_tindarius_DSM_2278 |
| WP_023845215_1 | CheY | Rec and PAS domains | Methanolobus_tindarius_DSM_2278 |
| WP_023845216_1 | - | Signal transduction histidine kinase, contains PAS domains | Methanolobus_tindarius_DSM_2278 |
| WP_023845217_1 | - | Transcriptional regulator, contains HTH domain | Methanolobus_tindarius_DSM_2278 |
| WP_023845218_1 | - | Transcriptional regulator, contains HTH domain | Methanolobus_tindarius_DSM_2278 |
| WP_023845219_1 | MtbC1 | Methanogenic corrinoid protein MtbC1 | Methanolobus_tindarius_DSM_2278 |
| WP_048135279_1 | MtmB | Monomethylamine methyltransferase | Methanolobus_tindarius_DSM_2278 |
| WP_048135281_1 | MtmB | Monomethylamine methyltransferase | Methanolobus_tindarius_DSM_2278 |
| WP_023845221_1 | PotE | Amino acid transporter | Methanolobus_tindarius_DSM_2278 |
| WP_023845222_1 | - | Uncharacterized membrane protein | Methanolobus_tindarius_DSM_2278 |
| WP_023845223_1 | AcrB | Cation/multidrug efflux pump | Methanolobus_tindarius_DSM_2278 |
| WP_023845224_1 | - | Desulfoferredoxin | Methanolobus_tindarius_DSM_2278 |
| WP_023845225_1 | MtbA | Methylcobalamin:coenzyme M methyltransferase | Methanolobus_tindarius_DSM_2278 |
| WP_048135282_1 | #N/A | #N/A | Methanolobus_tindarius_DSM_2278 |
| WP_023845229_1 | PotE | Amino acid transporter | Methanolobus_tindarius_DSM_2278 |
| WP_023845231_1 | MtbC1 | Methanogenic corrinoid protein MtbC1 | Methanolobus_tindarius_DSM_2278 |

| | | | |
|---|---|---|---|
| WP_048135283_1 | MtbB | Dimethylamine methyltransferase | Methanolobus_tindarius_DSM_2278 |
| WP_048135285_1 | MtbB | Dimethylamine methyltransferase | Methanolobus_tindarius_DSM_2278 |
| WP_023845233_1 | PotE | Amino acid transporter | Methanolobus_tindarius_DSM_2278 |
| WP_023845234_1 | - | Transcriptional regulator, contains HTH domain | Methanolobus_tindarius_DSM_2278 |
| WP_048135934_1 | CheY | Rec and PAS domains | Methanolobus_tindarius_DSM_2278 |
| WP_023845236_1 | - | Signal transduction histidine kinase, contains Cache and PAS domains | Methanolobus_tindarius_DSM_2278 |
| WP_023845237_1 | MtbC1 | Methanogenic corrinoid protein MtbC1 | Methanolobus_tindarius_DSM_2278 |
| WP_048135287_1 | MtmB | Monomethylamine methyltransferase | Methanolobus_tindarius_DSM_2278 |
| WP_084323991_1 | MtmB | Monomethylamine methyltransferase | Methanolobus_tindarius_DSM_2278 |
| WP_048135936_1 | FrvX | Peptidase M42 family protein | Methanolobus_tindarius_DSM_2278 |
| WP_023845240_1 | CydA | Cytochrome bd-type quinol oxidase, subunit I | Methanolobus_tindarius_DSM_2278 |
| WP_023845241_1 | AppB | Cytochrome bd-type quinol oxidase, subunit II | Methanolobus_tindarius_DSM_2278 |
| WP_023845242_1 | HyuA | N-methylhydantoinase A/acetone carboxylase, beta subunit | Methanolobus_tindarius_DSM_2278 |
| WP_023845243_1 | HyuA | N-methylhydantoinase A/acetone carboxylase, beta subunit | Methanolobus_tindarius_DSM_2278 |
| WP_048135290_1 | #N/A | #N/A | Methanolobus_tindarius_DSM_2278 |
| WP_023845244_1 | - | Sensory protein, contains PAS and GAF domains | Methanolobus_tindarius_DSM_2278 |
| WP_023845245_1 | HyuA | N-methylhydantoinase A/acetone carboxylase, beta subunit | Methanolobus_tindarius_DSM_2278 |
| WP_048135292_1 | MttB2 | Trimethylamine:corrinoid methyltransferase | Methanolobus_tindarius_DSM_2278 |
| WP_048135294_1 | MttB1 | Trimethylamine:corrinoid methyltransferase | Methanolobus_tindarius_DSM_2278 |
| WP_023845247_1 | MtbC1 | Methanogenic corrinoid protein MtbC1 | Methanolobus_tindarius_DSM_2278 |
| WP_023845248_1 | RhaT | Permease of the drug/metabolite transporter (DMT) superfamily | Methanolobus_tindarius_DSM_2278 |
| WP_023845249_1 | - | Uncharacterized protein | Methanolobus_tindarius_DSM_2278 |
| WP_023845251_1 | MtbC1 | Methanogenic corrinoid protein MtbC1 | Methanolobus_tindarius_DSM_2278 |
| WP_048135296_1 | MtbB | Dimethylamine methyltransferase | Methanolobus_tindarius_DSM_2278 |
| WP_084323992_1 | MtbB | Dimethylamine methyltransferase | Methanolobus_tindarius_DSM_2278 |
| WP_023845253_1 | MtbC1 | Methanogenic corrinoid protein MtbC1 | Methanolobus_tindarius_DSM_2278 |
| WP_048135298_1 | MtmB | Monomethylamine methyltransferase | Methanolobus_tindarius_DSM_2278 |
| WP_048135299_1 | MtmB | Monomethylamine methyltransferase | Methanolobus_tindarius_DSM_2278 |
| WP_023845255_1 | - | Uncharacterized membrane protein | Methanolobus_tindarius_DSM_2278 |
| WP_023845256_1 | MtmB | Monomethylamine methyltransferase | Methanolobus_tindarius_DSM_2278 |
| WP_023845257_1 | MtbA | Methylcobalamin:coenzyme M methyltransferase | Methanolobus_tindarius_DSM_2278 |
| WP_023845258_1 | TYW1 | Wybutosine (yW) biosynthesis enzyme, Fe-S oxidoreductase | Methanolobus_tindarius_DSM_2278 |
| WP_023845259_1 | eRF1 | Peptide chain release factor eRF1 | Methanolobus_tindarius_DSM_2278 |
| WP_023845260_1 | ArgS | Arginyl-tRNA synthetase | Methanolobus_tindarius_DSM_2278 |
| WP_023845261_1 | Trm5 | Wybutosine (yW) biosynthesis enzyme Trm5, tRNA (guanine) methyltransferase | Methanolobus_tindarius_DSM_2278 |

| | | | |
|---|---|---|---|
| WP_023845262_1 | OpuBB | ABC-type proline/glycine betaine transport system, permease component | Methanolobus_tindarius_DSM_2278 |
| WP_084324052_1 | OpuBB | ABC-type proline/glycine betaine transport system, permease component | Methanolobus_tindarius_DSM_2278 |
| WP_023845264_1 | OpuBA | ABC-type proline/glycine betaine transport system, ATPase component | Methanolobus_tindarius_DSM_2278 |
| WP_023845265_1 | OpuBC | Periplasmic glycine betaine/choline-binding (lipo)protein of an ABC-type transport system (osmoprotectant binding protein) | Methanolobus_tindarius_DSM_2278 |
| WP_023845266_1 | IlvB | Acetolactate synthase large subunit or other thiamine pyrophosphate-requiring enzyme | Methanolobus_tindarius_DSM_2278 |
| | | | Methermicoccus_shengliensis_DSM_18856 |
| WP_042686941_1 | SufC | Cysteine desulfurase activator ATPase | Methermicoccus_shengliensis_DSM_18856 |
| WP_084174137_1 | ZntA | TRASH/YHS-like protein, metallochaperone | Methermicoccus_shengliensis_DSM_18856 |
| WP_084174138_1 | DYS1 | Deoxyhypusine synthase | Methermicoccus_shengliensis_DSM_18856 |
| WP_042686889_1 | MtbC1 | Methanogenic corrinoid protein MtbC1 | Methermicoccus_shengliensis_DSM_18856 |
| WP_042686891_1 | - | Uncharacterized Fe-S clusters-containing protein, contains DUF4445 domain | Methermicoccus_shengliensis_DSM_18856 |
| WP_042686893_1 | - | CBS domain | Methermicoccus_shengliensis_DSM_18856 |
| WP_084174139_1 | - | Phytoene dehydrogenase or related enzyme | Methermicoccus_shengliensis_DSM_18856 |
| WP_084174140_1 | ACR3 | Arsenite efflux pump ACR3 or related permease | Methermicoccus_shengliensis_DSM_18856 |
| WP_084174141_1 | PotE | Amino acid transporter | Methermicoccus_shengliensis_DSM_18856 |
| WP_042686903_1 | MtbC1 | Methanogenic corrinoid protein MtbC1 | Methermicoccus_shengliensis_DSM_18856 |
| WP_042686905_1 | MtmB | Monomethylamine methyltransferase | Methermicoccus_shengliensis_DSM_18856 |
| WP_052353292_1 | - | Uncharacterized protein | Methermicoccus_shengliensis_DSM_18856 |
| WP_052353293_1 | RhaT | Permease of the drug/metabolite transporter (DMT) superfamily | Methermicoccus_shengliensis_DSM_18856 |
| WP_042686907_1 | MtbC1 | Methanogenic corrinoid protein MtbC1 | Methermicoccus_shengliensis_DSM_18856 |
| WP_084174142_1 | MttB1 | Trimethylamine:corrinoid methyltransferase | Methermicoccus_shengliensis_DSM_18856 |
| WP_042686911_1 | MttB2 | Trimethylamine:corrinoid methyltransferase | Methermicoccus_shengliensis_DSM_18856 |
| WP_042686948_1 | PylD | (3R)-3-methyl-D-ornithyl-N6-L-lysine dehydrogenase, pyrrolysine biosynthesis enzyme | Methermicoccus_shengliensis_DSM_18856 |
| WP_052353295_1 | PylC | (3R)-3-methyl-D-ornithine:L-lysine ligase, pyrrolysine biosynthesis protein | Methermicoccus_shengliensis_DSM_18856 |
| WP_052353296_1 | PylB/HcgA | (2R,3R)-3-methylornithine synthase involved in pyrrolysine biosynthesis/Iron-guanylylpyridinol (FeGP) cofactor biosynthesis protein HcgA | Methermicoccus_shengliensis_DSM_18856 |
| WP_042686913_1 | PylS | Pyrrolysyl-tRNA-synthetase | Methermicoccus_shengliensis_DSM_18856 |
| WP_042686915_1 | - | Transcriptional regulator, contains N-terminal RHH domain | Methermicoccus_shengliensis_DSM_18856 |
| WP_042686917_1 | #N/A | #N/A | Methermicoccus_shengliensis_DSM_18856 |
| WP_042686918_1 | CofE | F(420)-0:gamma-glutamyl ligase, F420 coenzyme biosynthesis enzyme | Methermicoccus_shengliensis_DSM_18856 |
| WP_042686920_1 | Tma20 | Predicted RNA-binding protein, contains PUA domain | Methermicoccus_shengliensis_DSM_18856 |
| WP_042686922_1 | LSM1 | Small nuclear ribonucleoprotein (snRNP) homolog | Methermicoccus_shengliensis_DSM_18856 |
| WP_084174143_1 | RPL37A | Ribosomal protein L37E | Methermicoccus_shengliensis_DSM_18856 |

| | | | |
|---|---|---|---|
| WP_042686954_1 | PurF | Glutamine phosphoribosylpyrophosphate amidotransferase | Methermicoccus_shengliensis_DSM_18856 |
| WP_042686923_1 | RPL21A | Ribosomal protein L21E | Methermicoccus_shengliensis_DSM_18856 |
| WP_042686925_1 | Rpo4 | DNA-directed RNA polymerase, subunit Rpo4/RpoF | Methermicoccus_shengliensis_DSM_18856 |
| WP_042686956_1 | - | Predicted RNA-binding protein | Methermicoccus_shengliensis_DSM_18856 |
| | | | Methanosarcina_soligelidi_SMA-21 |
| WP_080933756_1 | - | Uncharacterized protein | Methanosarcina_soligelidi_SMA-21 |
| WP_080503038_1 | - | Transposase, IS5 family | Methanosarcina_soligelidi_SMA-21 |
| WP_080503039_1 | #N/A | #N/A | Methanosarcina_soligelidi_SMA-21 |
| WP_011033379_1 | AcrB | Cation/multidrug efflux pump | Methanosarcina_soligelidi_SMA-21 |
| WP_011033380_1 | - | Uncharacterized membrane protein | Methanosarcina_soligelidi_SMA-21 |
| WP_011033381_1 | PotE | Amino acid transporter | Methanosarcina_soligelidi_SMA-21 |
| WP_048037606_1 | MtbC1 | Methanogenic corrinoid protein MtbC1 | Methanosarcina_soligelidi_SMA-21 |
| WP_048039091_1 | MtbA | Methylcobalamin:coenzyme M methyltransferase | Methanosarcina_soligelidi_SMA-21 |
| WP_048044826_1 | - | Uncharacterized Fe-S clusters-containing protein, contains DUF4445 domain | Methanosarcina_soligelidi_SMA-21 |
| WP_015411829_1 | Ppa | Inorganic pyrophosphatase | Methanosarcina_soligelidi_SMA-21 |
| WP_048050730_1 | PylD | (3R)-3-methyl-D-ornithyl-N6-L-lysine dehydrogenase, pyrrolysine biosynthesis enzyme | Methanosarcina_soligelidi_SMA-21 |
| WP_048050731_1 | PylC | (3R)-3-methyl-D-ornithine:L-lysine ligase, pyrrolysine biosynthesis protein | Methanosarcina_soligelidi_SMA-21 |
| WP_048050732_1 | PylB/HcgA | (2R,3R)-3-methylornithine synthase involved in pyrrolysine biosynthesis/Iron-guanylylpyridinol (FeGP) cofactor biosynthesis protein HcgA | Methanosarcina_soligelidi_SMA-21 |
| WP_048050733_1 | PylS | Pyrrolysyl-tRNA-synthetase | Methanosarcina_soligelidi_SMA-21 |
| WP_011033392_1 | #N/A | #N/A | Methanosarcina_soligelidi_SMA-21 |
| WP_048050734_1 | - | Class II terpene cyclase family protein | Methanosarcina_soligelidi_SMA-21 |
| WP_048037188_1 | - | Predicted DNA-binding protein | Methanosarcina_soligelidi_SMA-21 |
| WP_048050735_1 | - | Uncharacterized protein | Methanosarcina_soligelidi_SMA-21 |
| WP_015411833_1 | - | Homolog of Wybutosine (yW) biosynthesis enzyme, Fe-S oxidoreductase | Methanosarcina_soligelidi_SMA-21 |
| WP_048037168_1 | UspA | Nucleotide-binding protein, UspA family | Methanosarcina_soligelidi_SMA-21 |
| WP_080929520_1 | UspA | Nucleotide-binding protein, UspA family | Methanosarcina_soligelidi_SMA-21 |
| WP_048037167_1 | UspA | Nucleotide-binding protein, UspA family | Methanosarcina_soligelidi_SMA-21 |
| WP_015411836_1 | UspA | Nucleotide-binding protein, UspA family | Methanosarcina_soligelidi_SMA-21 |
| WP_015411837_1 | - | Uncharacterized protein | Methanosarcina_soligelidi_SMA-21 |
| | | | Methanosarcina_sp_2_H_T_1A_3 |
| WP_048136931_1 | YcaO | Ribosomal protein S12 methylthiotransferase accessory factor YcaO | Methanosarcina_sp_2_H_T_1A_3 |
| WP_048136933_1 | - | Uncharacterized protein | Methanosarcina_sp_2_H_T_1A_3 |
| WP_048136935_1 | - | Uncharacterized membrane protein | Methanosarcina_sp_2_H_T_1A_3 |

| | | | |
|---|---|---|---|
| WP_048136936_1 | - | Uncharacterized protein | Methanosarcina_sp_2_H_T_1A_3 |
| WP_048136938_1 | UspA | Nucleotide-binding protein, UspA family | Methanosarcina_sp_2_H_T_1A_3 |
| WP_048136940_1 | UspA | Nucleotide-binding protein, UspA family | Methanosarcina_sp_2_H_T_1A_3 |
| WP_048136942_1 | UspA | Nucleotide-binding protein, UspA family | Methanosarcina_sp_2_H_T_1A_3 |
| WP_048136944_1 | Ftn | Ferritin | Methanosarcina_sp_2_H_T_1A_3 |
| WP_048134875_1 | - | Predicted DNA-binding protein | Methanosarcina_sp_2_H_T_1A_3 |
| WP_048136946_1 | - | Class II terpene cyclase family protein | Methanosarcina_sp_2_H_T_1A_3 |
| WP_048136948_1 | PylS | Pyrrolysyl-tRNA-synthetase | Methanosarcina_sp_2_H_T_1A_3 |
| WP_048134872_1 | PylB/HcgA | (2R,3R)-3-methylornithine synthase involved in pyrrolysine biosynthesis/Iron-guanylylpyridinol (FeGP) cofactor biosynthesis protein HcgA | Methanosarcina_sp_2_H_T_1A_3 |
| WP_048136950_1 | PylC | (3R)-3-methyl-D-ornithine:L-lysine ligase, pyrrolysine biosynthesis protein | Methanosarcina_sp_2_H_T_1A_3 |
| WP_048136952_1 | PylD | (3R)-3-methyl-D-ornithyl-N6-L-lysine dehydrogenase, pyrrolysine biosynthesis enzyme | Methanosarcina_sp_2_H_T_1A_3 |
| WP_048136954_1 | - | Uncharacterized Fe-S clusters-containing protein, contains DUF4445 domain | Methanosarcina_sp_2_H_T_1A_3 |
| WP_048136956_1 | - | SAM-dependent methyltransferase | Methanosarcina_sp_2_H_T_1A_3 |
| WP_048136958_1 | MdaB | NADPH-quinone reductase (modulator of drug activity B) | Methanosarcina_sp_2_H_T_1A_3 |
| WP_048136960_1 | MtbA | Methylcobalamin:coenzyme M methyltransferase | Methanosarcina_sp_2_H_T_1A_3 |
| | | | Methanosarcina_sp_2_H_T_1A_6 |
| WP_048136931_1 | YcaO | Ribosomal protein S12 methylthiotransferase accessory factor YcaO | Methanosarcina_sp_2_H_T_1A_6 |
| WP_048136933_1 | - | Uncharacterized protein | Methanosarcina_sp_2_H_T_1A_6 |
| WP_048136935_1 | - | Uncharacterized membrane protein | Methanosarcina_sp_2_H_T_1A_6 |
| WP_048136936_1 | - | Uncharacterized protein | Methanosarcina_sp_2_H_T_1A_6 |
| WP_048136938_1 | UspA | Nucleotide-binding protein, UspA family | Methanosarcina_sp_2_H_T_1A_6 |
| WP_048136940_1 | UspA | Nucleotide-binding protein, UspA family | Methanosarcina_sp_2_H_T_1A_6 |
| WP_048136942_1 | UspA | Nucleotide-binding protein, UspA family | Methanosarcina_sp_2_H_T_1A_6 |
| WP_048136944_1 | Ftn | Ferritin | Methanosarcina_sp_2_H_T_1A_6 |
| WP_048134875_1 | - | Predicted DNA-binding protein | Methanosarcina_sp_2_H_T_1A_6 |
| WP_048136946_1 | - | Class II terpene cyclase family protein | Methanosarcina_sp_2_H_T_1A_6 |
| WP_048160059_1 | PylS | Pyrrolysyl-tRNA-synthetase | Methanosarcina_sp_2_H_T_1A_6 |
| WP_048134872_1 | PylB/HcgA | (2R,3R)-3-methylornithine synthase involved in pyrrolysine biosynthesis/Iron-guanylylpyridinol (FeGP) cofactor biosynthesis protein HcgA | Methanosarcina_sp_2_H_T_1A_6 |
| WP_048136950_1 | PylC | (3R)-3-methyl-D-ornithine:L-lysine ligase, pyrrolysine biosynthesis protein | Methanosarcina_sp_2_H_T_1A_6 |
| WP_048136952_1 | PylD | (3R)-3-methyl-D-ornithyl-N6-L-lysine dehydrogenase, pyrrolysine biosynthesis enzyme | Methanosarcina_sp_2_H_T_1A_6 |
| WP_048136954_1 | - | Uncharacterized Fe-S clusters-containing protein, contains DUF4445 domain | Methanosarcina_sp_2_H_T_1A_6 |

| | | | |
|---|---|---|---|
| WP_048136956_1 | - | SAM-dependent methyltransferase | Methanosarcina_sp_2_H_T_1A_6 |
| WP_048136958_1 | MdaB | NADPH-quinone reductase (modulator of drug activity B) | Methanosarcina_sp_2_H_T_1A_6 |
| WP_048136960_1 | MtbA | Methylcobalamin:coenzyme M methyltransferase | Methanosarcina_sp_2_H_T_1A_6 |
| WP_048144042_1 | MtbC1 | Methanogenic corrinoid protein MtbC1 | Methanosarcina_sp_2_H_T_1A_6 |
| WP_048133387_1 | MtmB | Monomethylamine methyltransferase | Methanosarcina_sp_2_H_T_1A_6 |
| WP_048141727_1 | MtmB | Monomethylamine methyltransferase | Methanosarcina_sp_2_H_T_1A_6 |
| WP_048142584_1 | PotE | Amino acid transporter | Methanosarcina_sp_2_H_T_1A_6 |
| WP_048142586_1 | - | Uncharacterized membrane protein | Methanosarcina_sp_2_H_T_1A_6 |
| WP_048142588_1 | AcrB | Cation/multidrug efflux pump | Methanosarcina_sp_2_H_T_1A_6 |
| WP_048142590_1 | RFCS | Clamp loader ATPase, small subunit | Methanosarcina_sp_2_H_T_1A_6 |
| WP_048142592_1 | SecD | Preprotein translocase subunit SecD | Methanosarcina_sp_2_H_T_1A_6 |
| WP_048142595_1 | SecF | Preprotein translocase subunit SecF | Methanosarcina_sp_2_H_T_1A_6 |
| WP_048142597_1 | ComEB | Deoxycytidylate deaminase | Methanosarcina_sp_2_H_T_1A_6 |
| WP_048142599_1 | CofE | F(420)-0:gamma-glutamyl ligase, F420 coenzyme biosynthesis enzyme | Methanosarcina_sp_2_H_T_1A_6 |
| WP_048142601_1 | - | Uncharacterized protein | Methanosarcina_sp_2_H_T_1A_6 |
| WP_048142603_1 | IbpA | Molecular chaperone (HSP20 family) | Methanosarcina_sp_2_H_T_1A_6 |
| | | | Methanosarcina_sp_2_H_T_1A_8 |
| WP_048136931_1 | YcaO | Ribosomal protein S12 methylthiotransferase accessory factor YcaO | Methanosarcina_sp_2_H_T_1A_8 |
| WP_048136933_1 | - | Uncharacterized protein | Methanosarcina_sp_2_H_T_1A_8 |
| WP_048136935_1 | - | Uncharacterized membrane protein | Methanosarcina_sp_2_H_T_1A_8 |
| WP_048136936_1 | - | Uncharacterized protein | Methanosarcina_sp_2_H_T_1A_8 |
| WP_048136938_1 | UspA | Nucleotide-binding protein, UspA family | Methanosarcina_sp_2_H_T_1A_8 |
| WP_048136940_1 | UspA | Nucleotide-binding protein, UspA family | Methanosarcina_sp_2_H_T_1A_8 |
| WP_048136942_1 | UspA | Nucleotide-binding protein, UspA family | Methanosarcina_sp_2_H_T_1A_8 |
| WP_048136944_1 | Ftn | Ferritin | Methanosarcina_sp_2_H_T_1A_8 |
| WP_048134875_1 | - | Predicted DNA-binding protein | Methanosarcina_sp_2_H_T_1A_8 |
| WP_048136946_1 | - | Class II terpene cyclase family protein | Methanosarcina_sp_2_H_T_1A_8 |
| WP_048136948_1 | PylS | Pyrrolysyl-tRNA-synthetase | Methanosarcina_sp_2_H_T_1A_8 |
| WP_048134872_1 | PylB/HcgA | (2R,3R)-3-methylornithine synthase involved in pyrrolysine biosynthesis/Iron-guanylylpyridinol (FeGP) cofactor biosynthesis protein HcgA | Methanosarcina_sp_2_H_T_1A_8 |
| WP_048136950_1 | PylC | (3R)-3-methyl-D-ornithine:L-lysine ligase, pyrrolysine biosynthesis protein | Methanosarcina_sp_2_H_T_1A_8 |
| WP_048136952_1 | PylD | (3R)-3-methyl-D-ornithyl-N6-L-lysine dehydrogenase, pyrrolysine biosynthesis enzyme | Methanosarcina_sp_2_H_T_1A_8 |
| WP_048136954_1 | - | Uncharacterized Fe-S clusters-containing protein, contains DUF4445 domain | Methanosarcina_sp_2_H_T_1A_8 |
| WP_048136956_1 | - | SAM-dependent methyltransferase | Methanosarcina_sp_2_H_T_1A_8 |

| | | | |
|---|---|---|---|
| WP_048136958_1 | MdaB | NADPH-quinone reductase (modulator of drug activity B) | Methanosarcina_sp_2_H_T_1A_8 |
| WP_048136960_1 | MtbA | Methylcobalamin:coenzyme M methyltransferase | Methanosarcina_sp_2_H_T_1A_8 |
| WP_048144042_1 | MtbC1 | Methanogenic corrinoid protein MtbC1 | Methanosarcina_sp_2_H_T_1A_8 |
| WP_048141725_1 | MtmB | Monomethylamine methyltransferase | Methanosarcina_sp_2_H_T_1A_8 |
| WP_048141727_1 | MtmB | Monomethylamine methyltransferase | Methanosarcina_sp_2_H_T_1A_8 |
| | | | Methanosarcina_flavescens_E03_2 |
| WP_054298905_1 | PylS | Pyrrolysyl-tRNA-synthetase | Methanosarcina_flavescens_E03_2 |
| WP_054298906_1 | PylB/HcgA | (2R,3R)-3-methylornithine synthase involved in pyrrolysine biosynthesis/Iron-guanylylpyridinol (FeGP) cofactor biosynthesis protein HcgA | Methanosarcina_flavescens_E03_2 |
| WP_054298907_1 | PylC | (3R)-3-methyl-D-ornithine:L-lysine ligase, pyrrolysine biosynthesis protein | Methanosarcina_flavescens_E03_2 |
| WP_054298908_1 | PylD | (3R)-3-methyl-D-ornithyl-N6-L-lysine dehydrogenase, pyrrolysine biosynthesis enzyme | Methanosarcina_flavescens_E03_2 |
| WP_054298909_1 | - | Uncharacterized Fe-S clusters-containing protein, contains DUF4445 domain | Methanosarcina_flavescens_E03_2 |
| WP_054298910_1 | - | SAM-dependent methyltransferase | Methanosarcina_flavescens_E03_2 |
| WP_054298911_1 | MtbA | Methylcobalamin:coenzyme M methyltransferase | Methanosarcina_flavescens_E03_2 |
| WP_054298912_1 | MtbC1 | Methanogenic corrinoid protein MtbC1 | Methanosarcina_flavescens_E03_2 |
| WP_054298913_1 | MtmB | Monomethylamine methyltransferase | Methanosarcina_flavescens_E03_2 |
| WP_054298914_1 | MtmB | Monomethylamine methyltransferase | Methanosarcina_flavescens_E03_2 |
| WP_082384229_1 | AmtB | Ammonia permease | Methanosarcina_flavescens_E03_2 |
| WP_054298915_1 | RFCS | Clamp loader ATPase, small subunit | Methanosarcina_flavescens_E03_2 |
| WP_054298916_1 | SecD | Preprotein translocase subunit SecD | Methanosarcina_flavescens_E03_2 |
| WP_054298917_1 | SecF | Preprotein translocase subunit SecF | Methanosarcina_flavescens_E03_2 |
| WP_054298918_1 | ComEB | Deoxycytidylate deaminase | Methanosarcina_flavescens_E03_2 |
| WP_054298919_1 | - | Cupin domain containing protein | Methanosarcina_flavescens_E03_2 |
| WP_054298920_1 | CofE | F(420)-0:gamma-glutamyl ligase, F420 coenzyme biosynthesis enzyme | Methanosarcina_flavescens_E03_2 |
| WP_054299028_1 | - | Uncharacterized protein | Methanosarcina_flavescens_E03_2 |
| WP_054298921_1 | MMT1 | Predicted Co/Zn/Cd cation transporter | Methanosarcina_flavescens_E03_2 |
| WP_054298922_1 | IbpA | Molecular chaperone (HSP20 family) | Methanosarcina_flavescens_E03_2 |
| | | | Methanonatronarchaeum_thermophilum_AMET1 |
| WP_086636630_1 | - | Predicted butyrate kinase | Methanonatronarchaeum_thermophilum_AMET1 |
| WP_086636631_1 | NapF | Ferredoxin | Methanonatronarchaeum_thermophilum_AMET1 |
| WP_086636632_1 | - | Signal transduction histidine kinase and HAMP domain | Methanonatronarchaeum_thermophilum_AMET1 |
| WP_086636633_1 | - | HEAT repeats containing protein | Methanonatronarchaeum_thermophilum_AMET1 |
| WP_086636634_1 | ThiN | Thiamine-phosphate synthase and thiamin biosynthesis regulator ThiR | Methanonatronarchaeum_thermophilum_AMET1 |
| WP_086636635_1 | HypE | Carbamoyl dehydratase HypE (hydrogenase maturation factor) | Methanonatronarchaeum_thermophilum_AMET1 |
| WP_086636636_1 | Lhr | Lhr-like helicase with C-terminal Zn finger domain | Methanonatronarchaeum_thermophilum_AMET1 |

| | | | |
|---|---|---|---|
| WP_086636637_1 | - | Signal transduction histidine kinase, contains PAS domain | Methanonatronarchaeum_thermophilum_AMET1 |
| WP_086636638_1 | Dfp | Phosphopantothenoylcysteine synthetase/decarboxylase | Methanonatronarchaeum_thermophilum_AMET1 |
| WP_086636639_1 | - | Signal transduction histidine kinase with PAS and PocR sensory domains | Methanonatronarchaeum_thermophilum_AMET1 |
| WP_086636640_1 | PylS | Pyrrolysyl-tRNA-synthetase | Methanonatronarchaeum_thermophilum_AMET1 |
| WP_086636641_1 | PylB/HcgA | (2R,3R)-3-methylornithine synthase involved in pyrrolysine biosynthesis/Iron-guanylylpyridinol (FeGP) cofactor biosynthesis protein HcgA | Methanonatronarchaeum_thermophilum_AMET1 |
| | | | Methanohalophilus_portucalensis_FDF-1T |
| WP_072358780_1 | - | MinD superfamily P-loop ATPase containing an inserted ferredoxin domain | Methanohalophilus_portucalensis_FDF-1T |
| WP_072358781_1 | - | MinD superfamily P-loop ATPase containing an inserted ferredoxin domain | Methanohalophilus_portucalensis_FDF-1T |
| WP_072358783_1 | FrhB | Coenzyme F420-reducing hydrogenase, beta subunit  fused to dissimilatory sulfite reductase | Methanohalophilus_portucalensis_FDF-1T |
| WP_072358785_1 | - | Predicted Fe-Mo cluster-binding protein, NifX family | Methanohalophilus_portucalensis_FDF-1T |
| WP_072358787_1 | - | Predicted Fe-Mo cluster-binding protein, NifX family | Methanohalophilus_portucalensis_FDF-1T |
| WP_072358789_1 | #N/A | #N/A | Methanohalophilus_portucalensis_FDF-1T |
| WP_072358791_1 | #N/A | #N/A | Methanohalophilus_portucalensis_FDF-1T |
| WP_072358793_1 | #N/A | #N/A | Methanohalophilus_portucalensis_FDF-1T |
| WP_072358795_1 | - | Transposase, IS5 family | Methanohalophilus_portucalensis_FDF-1T |
| WP_072358797_1 | MtbA | Methylcobalamin:coenzyme M methyltransferase | Methanohalophilus_portucalensis_FDF-1T |
| WP_072358799_1 | PylD | (3R)-3-methyl-D-ornithyl-N6-L-lysine dehydrogenase, pyrrolysine biosynthesis enzyme | Methanohalophilus_portucalensis_FDF-1T |
| WP_072358801_1 | PylC | (3R)-3-methyl-D-ornithine:L-lysine ligase, pyrrolysine biosynthesis protein | Methanohalophilus_portucalensis_FDF-1T |
| WP_099816343_1 | PylB/HcgA | (2R,3R)-3-methylornithine synthase involved in pyrrolysine biosynthesis/Iron-guanylylpyridinol (FeGP) cofactor biosynthesis protein HcgA | Methanohalophilus_portucalensis_FDF-1T |
| WP_072358806_1 | PylS | Pyrrolysyl-tRNA-synthetase | Methanohalophilus_portucalensis_FDF-1T |
| WP_072358808_1 | HisC | Histidinol-phosphate/aromatic aminotransferase or cobyric acid decarboxylase | Methanohalophilus_portucalensis_FDF-1T |
| WP_072358810_1 | - | Signal transduction histidine kinase, contains REC and PAS domains | Methanohalophilus_portucalensis_FDF-1T |
| WP_072358813_1 | - | Queuine tRNA-ribosyltransferase related protein | Methanohalophilus_portucalensis_FDF-1T |
| WP_072358815_1 | Sta1 | Viral transcriptional activator, contains HTH domain | Methanohalophilus_portucalensis_FDF-1T |
| WP_072358817_1 | - | MFS family permease | Methanohalophilus_portucalensis_FDF-1T |
| WP_072358819_1 | LabA | NYN domain, predicted PIN-related RNAse, tRNA/rRNA maturation | Methanohalophilus_portucalensis_FDF-1T |
| WP_072358821_1 | - | Aspartate/tyrosine/aromatic aminotransferase | Methanohalophilus_portucalensis_FDF-1T |
| WP_072358823_1 | - | DNA-binding transcriptional regulator, Lrp family | Methanohalophilus_portucalensis_FDF-1T |
| WP_072358825_1 | RPS8A | Ribosomal protein S8E | Methanohalophilus_portucalensis_FDF-1T |
| WP_072359597_1 | Rnz | Ribonuclease Z,  beta-lactamase superfamily hydrolase | Methanohalophilus_portucalensis_FDF-1T |
| | | | Methanohalophilus_euhalobius_DSM_10369 |
| WP_105460395_1 | #N/A | #N/A | Methanohalophilus_euhalobius_DSM_10369 |

| | | | |
|---|---|---|---|
| WP_105460396_1 | #N/A | #N/A | Methanohalophilus_euhalobius_DSM_10369 |
| WP_096713069_1 | - | Uncharacterized protein YjgD, DUF1641 family | Methanohalophilus_euhalobius_DSM_10369 |
| WP_105460397_1 | HcaD | NAD(FAD)-dependent dehydrogenase | Methanohalophilus_euhalobius_DSM_10369 |
| WP_096713071_1 | UspA | Nucleotide-binding protein,  UspA family | Methanohalophilus_euhalobius_DSM_10369 |
| WP_105460398_1 | - | Uncharacterized protein | Methanohalophilus_euhalobius_DSM_10369 |
| WP_105460399_1 | LeuC | Homoaconitate hydratase/3-isopropylmalate dehydratase large subunit family protein | Methanohalophilus_euhalobius_DSM_10369 |
| WP_105460400_1 | - | Transcriptional regulator MarR family, contains HTH domain | Methanohalophilus_euhalobius_DSM_10369 |
| WP_105460515_1 | SalX | ABC-type antimicrobial peptide transport system, ATPase component | Methanohalophilus_euhalobius_DSM_10369 |
| WP_105460401_1 | SalY | ABC-type antimicrobial peptide transport system, permease component | Methanohalophilus_euhalobius_DSM_10369 |
| WP_096712291_1 | PylS | Pyrrolysyl-tRNA-synthetase | Methanohalophilus_euhalobius_DSM_10369 |
| WP_105460516_1 | PylB/HcgA | (2R,3R)-3-methylornithine synthase involved in pyrrolysine biosynthesis/Iron-guanylylpyridinol (FeGP) cofactor biosynthesis protein HcgA | Methanohalophilus_euhalobius_DSM_10369 |
| WP_105460402_1 | PylC | (3R)-3-methyl-D-ornithine:L-lysine ligase, pyrrolysine biosynthesis protein | Methanohalophilus_euhalobius_DSM_10369 |
| WP_096712293_1 | PylD | (3R)-3-methyl-D-ornithyl-N6-L-lysine dehydrogenase, pyrrolysine biosynthesis enzyme | Methanohalophilus_euhalobius_DSM_10369 |
| WP_096712294_1 | MtbA | Methylcobalamin:coenzyme M methyltransferase | Methanohalophilus_euhalobius_DSM_10369 |
| WP_105460403_1 | - | Predicted Fe-Mo cluster-binding protein, NifX family | Methanohalophilus_euhalobius_DSM_10369 |
| WP_096712296_1 | - | Predicted Fe-Mo cluster-binding protein, NifX family | Methanohalophilus_euhalobius_DSM_10369 |
| WP_096712401_1 | - | MinD superfamily P-loop ATPase containing an inserted ferredoxin domain | Methanohalophilus_euhalobius_DSM_10369 |
| WP_105460404_1 | - | MinD superfamily P-loop ATPase containing an inserted ferredoxin domain | Methanohalophilus_euhalobius_DSM_10369 |
| WP_096712298_1 | - | Uncharacterized metal-binding protein, DUF1847 family | Methanohalophilus_euhalobius_DSM_10369 |
| WP_105460517_1 | - | Metal-dependent hydrolase of the beta-lactamase superfamily II | Methanohalophilus_euhalobius_DSM_10369 |
| WP_096712299_1 | - | Ferritin-like domain | Methanohalophilus_euhalobius_DSM_10369 |
| WP_105460405_1 | MgtC | Mg2+ transport system protein | Methanohalophilus_euhalobius_DSM_10369 |
| WP_096712301_1 | TroR | Mn-dependent transcriptional regulator (DtxR family) | Methanohalophilus_euhalobius_DSM_10369 |
| | | | Methanoculleus_sp_CAG_1088_ |
| CDF30930_1 | AlkD | 3-methyladenine DNA glycosylase AlkD | Methanoculleus_sp_CAG_1088_ |
| CDF30931_1 | Ada | Methylated DNA-protein cysteine methyltransferase | Methanoculleus_sp_CAG_1088_ |
| CDF30932_1 | - | DNA repair photolyase | Methanoculleus_sp_CAG_1088_ |
| CDF30933_1 | - | Predicted Na+-dependent transporter | Methanoculleus_sp_CAG_1088_ |
| CDF30934_1 | NapF | Flavodoxin fused to ferredoxin domain | Methanoculleus_sp_CAG_1088_ |
| CDF30935_1 | MenA | 1,4-dihydroxy-2-naphthoate octaprenyltransferase | Methanoculleus_sp_CAG_1088_ |
| CDF30936_1 | UbiE | Ubiquinone/menaquinone biosynthesis C-methylase UbiE | Methanoculleus_sp_CAG_1088_ |
| CDF30937_1 | FrvX | Peptidase M42 family protein | Methanoculleus_sp_CAG_1088_ |
| CDF30938_1 | PotE | Amino acid transporter | Methanoculleus_sp_CAG_1088_ |
| CDF30939_1 | MtbC1 | Methanogenic corrinoid protein MtbC1 | Methanoculleus_sp_CAG_1088_ |
| CDF30940_1 | MtbB | Dimethylamine methyltransferase | Methanoculleus_sp_CAG_1088_ |

| | | | |
|---|---|---|---|
| CDF30941_1 | MtbB | Dimethylamine methyltransferase | Methanoculleus_sp_CAG_1088_ |
| CDF30942_1 | MttB2 | Trimethylamine:corrinoid methyltransferase | Methanoculleus_sp_CAG_1088_ |
| CDF30943_1 | MttB1 | Trimethylamine:corrinoid methyltransferase | Methanoculleus_sp_CAG_1088_ |
| CDF30944_1 | MtbC1 | Methanogenic corrinoid protein MtbC1 | Methanoculleus_sp_CAG_1088_ |
| CDF30945_1 | RhaT | Permease of the drug/metabolite transporter (DMT) superfamily | Methanoculleus_sp_CAG_1088_ |
| CDF30946_1 | - | Uncharacterized protein | Methanoculleus_sp_CAG_1088_ |
| CDF30947_1 | #N/A | #N/A | Methanoculleus_sp_CAG_1088_ |
| CDF30948_1 | MtbC1 | Methanogenic corrinoid protein MtbC1 | Methanoculleus_sp_CAG_1088_ |
| CDF30949_1 | MtmB | Monomethylamine methyltransferase | Methanoculleus_sp_CAG_1088_ |
| CDF30950_1 | MtmB | Monomethylamine methyltransferase | Methanoculleus_sp_CAG_1088_ |
| CDF30951_1 | MtmB | Monomethylamine methyltransferase | Methanoculleus_sp_CAG_1088_ |
| CDF30952_1 | MtmB | Monomethylamine methyltransferase | Methanoculleus_sp_CAG_1088_ |
| CDF30953_1 | PylS | Pyrrolysyl-tRNA-synthetase | Methanoculleus_sp_CAG_1088_ |
| CDF30954_1 | PylC | (3R)-3-methyl-D-ornithine:L-lysine ligase, pyrrolysine biosynthesis protein | Methanoculleus_sp_CAG_1088_ |
| CDF30955_1 | PylD | (3R)-3-methyl-D-ornithyl-N6-L-lysine dehydrogenase, pyrrolysine biosynthesis enzyme | Methanoculleus_sp_CAG_1088_ |
| CDF30956_1 | - | Uncharacterized Fe-S clusters-containing protein, contains DUF4445 domain | Methanoculleus_sp_CAG_1088_ |
| CDF30957_1 | SrmB | Superfamily II DNA and RNA helicase | Methanoculleus_sp_CAG_1088_ |
| CDF30958_1 | - | AAA family ATPase | Methanoculleus_sp_CAG_1088_ |
| CDF30959_1 | - | AAA family ATPase | Methanoculleus_sp_CAG_1088_ |
| CDF30960_1 | #N/A | #N/A | Methanoculleus_sp_CAG_1088_ |
| CDF30961_1 | #N/A | #N/A | Methanoculleus_sp_CAG_1088_ |
| CDF30962_1 | #N/A | #N/A | Methanoculleus_sp_CAG_1088_ |
| CDF30963_1 | #N/A | #N/A | Methanoculleus_sp_CAG_1088_ |
| CDF30964_1 | - | Uncharacterized membrane protein, DUF1846 family | Methanoculleus_sp_CAG_1088_ |
| CDF30965_1 | EbsC | Cys-tRNA(Pro)/Cys-tRNA(Cys) deacylase, ybaK family | Methanoculleus_sp_CAG_1088_ |
| | | | Methanosarcina_sp_795_ |
| ALK06575_1 | - | Uncharacterized protein | Methanosarcina_sp_795_ |
| ALK04952_1 | CofE | F(420)-0:gamma-glutamyl ligase, F420 coenzyme biosynthesis enzyme | Methanosarcina_sp_795_ |
| ALK04953_1 | - | Cupin domain containing protein | Methanosarcina_sp_795_ |
| ALK04954_1 | ComEB | Deoxycytidylate deaminase | Methanosarcina_sp_795_ |
| ALK04955_1 | SecF | Preprotein translocase subunit SecF | Methanosarcina_sp_795_ |
| ALK04956_1 | SecD | Preprotein translocase subunit SecD | Methanosarcina_sp_795_ |
| ALK04957_1 | #N/A | #N/A | Methanosarcina_sp_795_ |
| ALK04958_1 | AcrB | Cation/multidrug efflux pump | Methanosarcina_sp_795_ |
| ALK04959_1 | - | Uncharacterized membrane protein | Methanosarcina_sp_795_ |
| ALK04960_1 | PotE | Amino acid transporter | Methanosarcina_sp_795_ |
| ALK04961_1 | MtmB | Monomethylamine methyltransferase | Methanosarcina_sp_795_ |
| ALK04962_1 | MtmB | Monomethylamine methyltransferase | Methanosarcina_sp_795_ |
| ALK04963_1 | MtbC1 | Methanogenic corrinoid protein MtbC1 | Methanosarcina_sp_795_ |
| ALK04964_1 | MtbA | Methylcobalamin:coenzyme M methyltransferase | Methanosarcina_sp_795_ |
| ALK04965_1 | - | SAM-dependent methyltransferase | Methanosarcina_sp_795_ |

| | | | |
|---|---|---|---|
| ALK04966_1 | - | Uncharacterized Fe-S clusters-containing protein, contains DUF4445 domain | Methanosarcina_sp_795_ |
| ALK04967_1 | PylD | (3R)-3-methyl-D-ornithyl-N6-L-lysine dehydrogenase, pyrrolysine biosynthesis enzyme | Methanosarcina_sp_795_ |
| ALK04968_1 | PylC | (3R)-3-methyl-D-ornithine:L-lysine ligase, pyrrolysine biosynthesis protein | Methanosarcina_sp_795_ |
| ALK04969_1 | PylB/HcgA | (2R,3R)-3-methylornithine synthase involved in pyrrolysine biosynthesis/Iron-guanylylpyridinol (FeGP) cofactor biosynthesis protein HcgA | Methanosarcina_sp_795_ |
| ALK04970_1 | PylS | Pyrrolysyl-tRNA-synthetase | Methanosarcina_sp_795_ |
| ALK04971_1 | - | Uncharacterized protein, DUF1638 family | Methanosarcina_sp_795_ |
| ALK04972_1 | Ftn | Ferritin | Methanosarcina_sp_795_ |
| ALK04973_1 | UspA | Nucleotide-binding protein, UspA family | Methanosarcina_sp_795_ |
| ALK04974_1 | UspA | Nucleotide-binding protein, UspA family | Methanosarcina_sp_795_ |
| ALK04975_1 | - | Uncharacterized protein | Methanosarcina_sp_795_ |
| ALK04976_1 | YcaO | Ribosomal protein S12 methylthiotransferase accessory factor YcaO | Methanosarcina_sp_795_ |
| ALK04977_1 | - | Uncharacterized protein, DUF2892 family | Methanosarcina_sp_795_ |
| ALK04978_1 | - | Uncharacterized protein | Methanosarcina_sp_795_ |
| ALK04979_1 | #N/A | #N/A | Methanosarcina_sp_795_ |
| ALK04980_1 | #N/A | #N/A | Methanosarcina_sp_795_ |
| | | | Euryarchaeota_archaeon_55_53_ |
| KUK04045_1 | RsmA | 16S rRNA A1518 and A1519 N6-dimethyltransferase RsmA/KsgA/DIM1 | Euryarchaeota_archaeon_55_53_ |
| KUK04046_1 | - | Predicted RNA-binding protein | Euryarchaeota_archaeon_55_53_ |
| KUK04047_1 | Rpo4 | DNA-directed RNA polymerase, subunit Rpo4/RpoF | Euryarchaeota_archaeon_55_53_ |
| KUK04048_1 | RPL21A | Ribosomal protein L21E | Euryarchaeota_archaeon_55_53_ |
| KUK04049_1 | PurF | Glutamine phosphoribosylpyrophosphate amidotransferase | Euryarchaeota_archaeon_55_53_ |
| KUK04050_1 | LSM1 | Small nuclear ribonucleoprotein (snRNP) homolog | Euryarchaeota_archaeon_55_53_ |
| KUK04051_1 | Tma20 | Predicted RNA-binding protein, contains PUA domain | Euryarchaeota_archaeon_55_53_ |
| KUK04052_1 | CofE | F(420)-0:gamma-glutamyl ligase, F420 coenzyme biosynthesis enzyme | Euryarchaeota_archaeon_55_53_ |
| KUK04053_1 | #N/A | #N/A | Euryarchaeota_archaeon_55_53_ |
| KUK04054_1 | - | Transcriptional regulator, contains N-terminal RHH domain | Euryarchaeota_archaeon_55_53_ |
| KUK04055_1 | PylS | Pyrrolysyl-tRNA-synthetase | Euryarchaeota_archaeon_55_53_ |
| KUK04056_1 | PylB/HcgA | (2R,3R)-3-methylornithine synthase involved in pyrrolysine biosynthesis/Iron-guanylylpyridinol (FeGP) cofactor biosynthesis protein HcgA | Euryarchaeota_archaeon_55_53_ |
| KUK04057_1 | PylC | (3R)-3-methyl-D-ornithine:L-lysine ligase, pyrrolysine biosynthesis protein | Euryarchaeota_archaeon_55_53_ |
| KUK04058_1 | PylD | (3R)-3-methyl-D-ornithyl-N6-L-lysine dehydrogenase, pyrrolysine biosynthesis enzyme | Euryarchaeota_archaeon_55_53_ |
| KUK04059_1 | MttB2 | Trimethylamine:corrinoid methyltransferase | Euryarchaeota_archaeon_55_53_ |
| KUK04060_1 | MttB1 | Trimethylamine:corrinoid methyltransferase | Euryarchaeota_archaeon_55_53_ |
| KUK04061_1 | MtbC1 | Methanogenic corrinoid protein MtbC1 | Euryarchaeota_archaeon_55_53_ |
| KUK04062_1 | RhaT | Permease of the drug/metabolite transporter (DMT) superfamily | Euryarchaeota_archaeon_55_53_ |
| KUK04063_1 | - | Uncharacterized protein | Euryarchaeota_archaeon_55_53_ |

| | | | |
|---|---|---|---|
| KUK04064_1 | - | Uncharacterized protein | Euryarchaeota_archaeon_55_53_ |
| KUK04065_1 | MtmB | Monomethylamine methyltransferase | Euryarchaeota_archaeon_55_53_ |
| | | | Methanosarcinales_archeaon_56_1174_ |
| KUK29526_1 | PylS | Pyrrolysyl-tRNA-synthetase | Methanosarcinales_archeaon_56_1174_ |
| KUK29527_1 | - | Transcriptional regulator, contains N-terminal RHH domain | Methanosarcinales_archeaon_56_1174_ |
| KUK29528_1 | #N/A | #N/A | Methanosarcinales_archeaon_56_1174_ |
| KUK29529_1 | CofE | F(420)-0:gamma-glutamyl ligase, F420 coenzyme biosynthesis enzyme | Methanosarcinales_archeaon_56_1174_ |
| KUK29530_1 | Tma20 | Predicted RNA-binding protein, contains PUA domain | Methanosarcinales_archeaon_56_1174_ |
| KUK29531_1 | LSM1 | Small nuclear ribonucleoprotein (snRNP) homolog | Methanosarcinales_archeaon_56_1174_ |
| KUK29532_1 | PurF | Glutamine phosphoribosylpyrophosphate amidotransferase | Methanosarcinales_archeaon_56_1174_ |
| KUK29533_1 | RPL21A | Ribosomal protein L21E | Methanosarcinales_archeaon_56_1174_ |
| KUK29534_1 | Rpo4 | DNA-directed RNA polymerase, subunit Rpo4/RpoF | Methanosarcinales_archeaon_56_1174_ |
| KUK29535_1 | - | Predicted RNA-binding protein | Methanosarcinales_archeaon_56_1174_ |
| KUK29536_1 | RsmA | 16S rRNA A1518 and A1519 N6-dimethyltransferase RsmA/KsgA/DIM1 | Methanosarcinales_archeaon_56_1174_ |
| | | | Methanohalophilus_sp_DAL1_ |
| OBZ34602_1 | MgtC | Mg2+ transport system protein | Methanohalophilus_sp_DAL1_ |
| OBZ34603_1 | - | Ferritin-like domain | Methanohalophilus_sp_DAL1_ |
| OBZ34619_1 | - | Metal-dependent hydrolase of the beta-lactamase superfamily II | Methanohalophilus_sp_DAL1_ |
| OBZ34604_1 | - | Uncharacterized metal-binding protein, DUF1847 family | Methanohalophilus_sp_DAL1_ |
| OBZ34605_1 | - | MinD superfamily P-loop ATPase containing an inserted ferredoxin domain | Methanohalophilus_sp_DAL1_ |
| OBZ34620_1 | - | MinD superfamily P-loop ATPase containing an inserted ferredoxin domain | Methanohalophilus_sp_DAL1_ |
| OBZ34606_1 | - | Predicted Fe-Mo cluster-binding protein, NifX family | Methanohalophilus_sp_DAL1_ |
| OBZ34607_1 | - | Predicted Fe-Mo cluster-binding protein, NifX family | Methanohalophilus_sp_DAL1_ |
| OBZ34608_1 | - | Transposase, IS5 family | Methanohalophilus_sp_DAL1_ |
| OBZ34609_1 | MtbA | Methylcobalamin:coenzyme M methyltransferase | Methanohalophilus_sp_DAL1_ |
| OBZ34610_1 | PylD | (3R)-3-methyl-D-ornithyl-N6-L-lysine dehydrogenase, pyrrolysine biosynthesis enzyme | Methanohalophilus_sp_DAL1_ |
| OBZ34611_1 | PylC | (3R)-3-methyl-D-ornithine:L-lysine ligase, pyrrolysine biosynthesis protein | Methanohalophilus_sp_DAL1_ |
| OBZ34612_1 | PylB/HcgA | (2R,3R)-3-methylornithine synthase involved in pyrrolysine biosynthesis/Iron-guanylylpyridinol (FeGP) cofactor biosynthesis protein HcgA | Methanohalophilus_sp_DAL1_ |
| OBZ34613_1 | PylS | Pyrrolysyl-tRNA-synthetase | Methanohalophilus_sp_DAL1_ |
| | | | Methanohalophilus_sp_DAL1_ |
| OBZ34498_1 | PylD | (3R)-3-methyl-D-ornithyl-N6-L-lysine dehydrogenase, pyrrolysine biosynthesis enzyme | Methanohalophilus_sp_DAL1_ |
| OBZ34499_1 | PylC | (3R)-3-methyl-D-ornithine:L-lysine ligase, pyrrolysine biosynthesis protein | Methanohalophilus_sp_DAL1_ |
| OBZ34500_1 | PylB/HcgA | (2R,3R)-3-methylornithine synthase involved in pyrrolysine biosynthesis/Iron-guanylylpyridinol (FeGP) cofactor biosynthesis protein HcgA | Methanohalophilus_sp_DAL1_ |
| OBZ34501_1 | PylS | Pyrrolysyl-tRNA-synthetase | Methanohalophilus_sp_DAL1_ |
| | | | Methanohalophilus_sp_2-GBenrich_ |

| | | | |
|---|---|---|---|
| ODV50254_1 | SalX | ABC-type antimicrobial peptide transport system, ATPase component | Methanohalophilus_sp_2-GBenrich_ |
| ODV50255_1 | - | S-layer domain | Methanohalophilus_sp_2-GBenrich_ |
| ODV50256_1 | SalY | ABC-type antimicrobial peptide transport system, permease component | Methanohalophilus_sp_2-GBenrich_ |
| ODV50257_1 | PylS | Pyrrolysyl-tRNA-synthetase | Methanohalophilus_sp_2-GBenrich_ |
| ODV50258_1 | PylB/HcgA | (2R,3R)-3-methylornithine synthase involved in pyrrolysine biosynthesis/Iron-guanylylpyridinol (FeGP) cofactor biosynthesis protein HcgA | Methanohalophilus_sp_2-GBenrich_ |
| ODV50259_1 | PylC | (3R)-3-methyl-D-ornithine:L-lysine ligase, pyrrolysine biosynthesis protein | Methanohalophilus_sp_2-GBenrich_ |
| ODV50260_1 | PylD | (3R)-3-methyl-D-ornithyl-N6-L-lysine dehydrogenase, pyrrolysine biosynthesis enzyme | Methanohalophilus_sp_2-GBenrich_ |
| ODV50261_1 | MtbA | Methylcobalamin:coenzyme M methyltransferase | Methanohalophilus_sp_2-GBenrich_ |
| ODV50262_1 | - | Predicted Fe-Mo cluster-binding protein, NifX family | Methanohalophilus_sp_2-GBenrich_ |
| ODV50263_1 | - | Predicted Fe-Mo cluster-binding protein, NifX family | Methanohalophilus_sp_2-GBenrich_ |
| ODV50264_1 | - | MinD superfamily P-loop ATPase containing an inserted ferredoxin domain | Methanohalophilus_sp_2-GBenrich_ |
| ODV50265_1 | - | MinD superfamily P-loop ATPase containing an inserted ferredoxin domain | Methanohalophilus_sp_2-GBenrich_ |
| ODV50266_1 | - | Uncharacterized metal-binding protein, DUF1847 family | Methanohalophilus_sp_2-GBenrich_ |
| ODV50267_1 | - | Metal-dependent hydrolase of the beta-lactamase superfamily II | Methanohalophilus_sp_2-GBenrich_ |
| ODV50268_1 | - | Ferritin-like domain | Methanohalophilus_sp_2-GBenrich_ |
| ODV50269_1 | MgtC | Mg2+ transport system protein | Methanohalophilus_sp_2-GBenrich_ |
| ODV50270_1 | TroR | Mn-dependent transcriptional regulator (DtxR family) | Methanohalophilus_sp_2-GBenrich_ |
| | | | Candidatus_Methanohalarchaeum_thermophilum_ |
| OKY77535_1 | #N/A | #N/A | Candidatus_Methanohalarchaeum_thermophilum_ |
| OKY77536_1 | MtbC1 | Methanogenic corrinoid protein MtbC1 | Candidatus_Methanohalarchaeum_thermophilum_ |
| OKY77537_1 | MtmB | Monomethylamine methyltransferase | Candidatus_Methanohalarchaeum_thermophilum_ |
| OKY77538_1 | MtmB | Monomethylamine methyltransferase | Candidatus_Methanohalarchaeum_thermophilum_ |
| OKY77539_1 | PylD | (3R)-3-methyl-D-ornithyl-N6-L-lysine dehydrogenase, pyrrolysine biosynthesis enzyme | Candidatus_Methanohalarchaeum_thermophilum_ |
| OKY77540_1 | PylC | (3R)-3-methyl-D-ornithine:L-lysine ligase, pyrrolysine biosynthesis protein | Candidatus_Methanohalarchaeum_thermophilum_ |
| OKY77541_1 | - | Uncharacterized protein | Candidatus_Methanohalarchaeum_thermophilum_ |
| OKY77542_1 | - | Uncharacterized protein | Candidatus_Methanohalarchaeum_thermophilum_ |
| OKY77543_1 | RhaT | Permease of the drug/metabolite transporter (DMT) superfamily | Candidatus_Methanohalarchaeum_thermophilum_ |
| OKY77544_1 | SbcC | ATPase involved in DNA repair, SbcC | Candidatus_Methanohalarchaeum_thermophilum_ |
| OKY77545_1 | - | PAS domain | Candidatus_Methanohalarchaeum_thermophilum_ |
| OKY77546_1 | MtbC1 | Methanogenic corrinoid protein MtbC1 | Candidatus_Methanohalarchaeum_thermophilum_ |
| OKY77547_1 | MtbB | Dimethylamine methyltransferase | Candidatus_Methanohalarchaeum_thermophilum_ |
| OKY77548_1 | MtbB | Dimethylamine methyltransferase | Candidatus_Methanohalarchaeum_thermophilum_ |
| OKY77549_1 | MttB1 | Trimethylamine:corrinoid methyltransferase | Candidatus_Methanohalarchaeum_thermophilum_ |
| OKY77550_1 | MttB2 | Trimethylamine:corrinoid methyltransferase | Candidatus_Methanohalarchaeum_thermophilum_ |
| OKY77551_1 | PylB/HcgA | (2R,3R)-3-methylornithine synthase involved in pyrrolysine biosynthesis/Iron-guanylylpyridinol (FeGP) cofactor biosynthesis protein HcgA | Candidatus_Methanohalarchaeum_thermophilum_ |
| OKY77552_1 | PylS | Pyrrolysyl-tRNA-synthetase | Candidatus_Methanohalarchaeum_thermophilum_ |

| | | | |
|---|---|---|---|
| OKY77553_1 | MtbC1 | Methanogenic corrinoid protein MtbC1 | Candidatus_Methanohalarchaeum_thermophilum_ |
| OKY77554_1 | HxlR | DNA-binding transcriptional regulator, HxlR family | Candidatus_Methanohalarchaeum_thermophilum_ |
| OKY77555_1 | #N/A | #N/A | Candidatus_Methanohalarchaeum_thermophilum_ |
| OKY77556_1 | - | Minimal nucleotidyltransferase | Candidatus_Methanohalarchaeum_thermophilum_ |
| OKY77557_1 | - | HEPN domain containing protein | Candidatus_Methanohalarchaeum_thermophilum_ |
| OKY77558_1 | #N/A | #N/A | Candidatus_Methanohalarchaeum_thermophilum_ |
| OKY77559_1 | LeuB | Isocitrate/isopropylmalate dehydrogenase | Candidatus_Methanohalarchaeum_thermophilum_ |
| OKY77560_1 | LeuD | 3-isopropylmalate dehydratase small subunit | Candidatus_Methanohalarchaeum_thermophilum_ |
| OKY77561_1 | LeuC | Homoaconitate hydratase/3-isopropylmalate dehydratase large subunit family protein | Candidatus_Methanohalarchaeum_thermophilum_ |
| OKY77562_1 | - | Transposable element, IS605 OrfB family, contains RNAse H fold nuclease and Zn finger domains | Candidatus_Methanohalarchaeum_thermophilum_ |
| | | | Candidatus_Methanohalarchaeum_thermophilum_ |
| OKY79085_1 | LepB | Signal peptidase I | Candidatus_Methanohalarchaeum_thermophilum_ |
| OKY79086_1 | - | M73 family secreted endopeptidase | Candidatus_Methanohalarchaeum_thermophilum_ |
| OKY79087_1 | - | M73 family secreted endopeptidase | Candidatus_Methanohalarchaeum_thermophilum_ |
| OKY79088_1 | ArsR | Transcriptional regulator containing HTH domain, ArsR family | Candidatus_Methanohalarchaeum_thermophilum_ |
| OKY79089_1 | - | Transcriptional regulator, contains HTH domain | Candidatus_Methanohalarchaeum_thermophilum_ |
| OKY79090_1 | - | Transposable element, IS605 OrfB family, contains RNAse H fold nuclease and Zn finger domains | Candidatus_Methanohalarchaeum_thermophilum_ |
| OKY79091_1 | #N/A | #N/A | Candidatus_Methanohalarchaeum_thermophilum_ |
| OKY79092_1 | RAYT | REP element-mobilizing transposase RayT | Candidatus_Methanohalarchaeum_thermophilum_ |
| OKY79093_1 | - | Radical SAM superfamily enzyme | Candidatus_Methanohalarchaeum_thermophilum_ |
| OKY79094_1 | - | Uncharacterized protein | Candidatus_Methanohalarchaeum_thermophilum_ |
| OKY79095_1 | PylB/HcgA | (2R,3R)-3-methylornithine synthase involved in pyrrolysine biosynthesis/Iron-guanylylpyridinol (FeGP) cofactor biosynthesis protein HcgA | Candidatus_Methanohalarchaeum_thermophilum_ |
| OKY79096_1 | PylS | Pyrrolysyl-tRNA-synthetase | Candidatus_Methanohalarchaeum_thermophilum_ |
| OKY79097_1 | AmtB | Ammonia permease | Candidatus_Methanohalarchaeum_thermophilum_ |
| OKY79098_1 | MtmB | Monomethylamine methyltransferase | Candidatus_Methanohalarchaeum_thermophilum_ |
| OKY79099_1 | MtmB | Monomethylamine methyltransferase | Candidatus_Methanohalarchaeum_thermophilum_ |
| OKY79100_1 | MtbC1 | Methanogenic corrinoid protein MtbC1 | Candidatus_Methanohalarchaeum_thermophilum_ |
| OKY79101_1 | HxlR | DNA-binding transcriptional regulator, HxlR family | Candidatus_Methanohalarchaeum_thermophilum_ |
| OKY79102_1 | #N/A | #N/A | Candidatus_Methanohalarchaeum_thermophilum_ |
| OKY79103_1 | - | Putative selenium binding protein, beta/alpha-propeller fold | Candidatus_Methanohalarchaeum_thermophilum_ |
| OKY79104_1 | #N/A | #N/A | Candidatus_Methanohalarchaeum_thermophilum_ |
| OKY79105_1 | #N/A | #N/A | Candidatus_Methanohalarchaeum_thermophilum_ |
| OKY79106_1 | ArsR | Transcriptional regulator containing HTH domain, ArsR family | Candidatus_Methanohalarchaeum_thermophilum_ |
| OKY79107_1 | CcmA | ABC-type multidrug transport system, ATPase component | Candidatus_Methanohalarchaeum_thermophilum_ |
| OKY79108_1 | - | ABC-type multidrug transport system, permease component | Candidatus_Methanohalarchaeum_thermophilum_ |
| OKY79109_1 | #N/A | #N/A | Candidatus_Methanohalarchaeum_thermophilum_ |
| | | | Methanosarcina_sp_2_H_A_1B_4 |

| | | | |
|---|---|---|---|
| WP_048169743_1 | - | Uncharacterized membrane protein | Methanosarcina_sp_2_H_A_1B_4 |
| WP_048169745_1 | - | Uncharacterized protein | Methanosarcina_sp_2_H_A_1B_4 |
| WP_048169746_1 | UspA | Nucleotide-binding protein, UspA family | Methanosarcina_sp_2_H_A_1B_4 |
| WP_048169749_1 | UspA | Nucleotide-binding protein, UspA family | Methanosarcina_sp_2_H_A_1B_4 |
| WP_048169750_1 | UspA | Nucleotide-binding protein, UspA family | Methanosarcina_sp_2_H_A_1B_4 |
| WP_048169752_1 | Ftn | Ferritin | Methanosarcina_sp_2_H_A_1B_4 |
| WP_048169754_1 | - | Homolog of Wybutosine (yW) biosynthesis enzyme, Fe-S oxidoreductase | Methanosarcina_sp_2_H_A_1B_4 |
| WP_048169756_1 | - | Uncharacterized protein | Methanosarcina_sp_2_H_A_1B_4 |
| WP_082107946_1 | - | Predicted DNA-binding protein | Methanosarcina_sp_2_H_A_1B_4 |
| WP_048169760_1 | #N/A | #N/A | Methanosarcina_sp_2_H_A_1B_4 |
| WP_048169762_1 | PylS | Pyrrolysyl-tRNA-synthetase | Methanosarcina_sp_2_H_A_1B_4 |
| WP_048169764_1 | PylB/HcgA | (2R,3R)-3-methylornithine synthase involved in pyrrolysine biosynthesis/Iron-guanylylpyridinol (FeGP) cofactor biosynthesis protein HcgA | Methanosarcina_sp_2_H_A_1B_4 |
| WP_048169765_1 | PylC | (3R)-3-methyl-D-ornithine:L-lysine ligase, pyrrolysine biosynthesis protein | Methanosarcina_sp_2_H_A_1B_4 |
| WP_048134870_1 | PylD | (3R)-3-methyl-D-ornithyl-N6-L-lysine dehydrogenase, pyrrolysine biosynthesis enzyme | Methanosarcina_sp_2_H_A_1B_4 |
| WP_048169767_1 | - | Uncharacterized Fe-S clusters-containing protein, contains DUF4445 domain | Methanosarcina_sp_2_H_A_1B_4 |
| WP_048169768_1 | MtbA | Methylcobalamin:coenzyme M methyltransferase | Methanosarcina_sp_2_H_A_1B_4 |
| WP_048169770_1 | MtbC1 | Methanogenic corrinoid protein MtbC1 | Methanosarcina_sp_2_H_A_1B_4 |
| WP_048169772_1 | MtmB | Monomethylamine methyltransferase | Methanosarcina_sp_2_H_A_1B_4 |
| WP_048133389_1 | MtmB | Monomethylamine methyltransferase | Methanosarcina_sp_2_H_A_1B_4 |
| WP_048169774_1 | PotE | Amino acid transporter | Methanosarcina_sp_2_H_A_1B_4 |
| WP_048169776_1 | - | Uncharacterized membrane protein | Methanosarcina_sp_2_H_A_1B_4 |
| WP_048128719_1 | AcrB | Cation/multidrug efflux pump | Methanosarcina_sp_2_H_A_1B_4 |
| WP_048169777_1 | RFCS | Clamp loader ATPase, small subunit | Methanosarcina_sp_2_H_A_1B_4 |
| WP_048169779_1 | SecD | Preprotein translocase subunit SecD | Methanosarcina_sp_2_H_A_1B_4 |
| WP_048132054_1 | SecF | Preprotein translocase subunit SecF | Methanosarcina_sp_2_H_A_1B_4 |
| WP_048160477_1 | ComEB | Deoxycytidylate deaminase | Methanosarcina_sp_2_H_A_1B_4 |
| WP_048169781_1 | CofE | F(420)-0:gamma-glutamyl ligase, F420 coenzyme biosynthesis enzyme | Methanosarcina_sp_2_H_A_1B_4 |
| WP_048160479_1 | - | Uncharacterized protein | Methanosarcina_sp_2_H_A_1B_4 |
| WP_048132046_1 | IbpA | Molecular chaperone (HSP20 family) | Methanosarcina_sp_2_H_A_1B_4 |

**Note:** The PylS proteins are highlighted.

**Table S5.** Summarization of targeted peptides via LC-MS/MS.

| Sequence | Peptide-Spectrum Matches (PSMs) | Identification | Exp Value |
|---|---|---|---|
| **PylRS2 variants sV1 (DSKN) and $tRNA^{Pyl}2$** | | | |
| VDVDQDATVGDALDALVGAHPALESR | 1 | Q | $1.1091 \times E^{-9}$ |
| VDVDKDATVGDALDALVGAHPALESR | 1 | K | $2.80524 \times E^{-9}$ |
| **PylRS2 variants sV6 (SGKN) and $tRNA^{Pyl}2$** | | | |
| TVRVDVDEDATVGDALDALVGAHPALESR | 4 | E | $8.2979 \times E^{-13}$ |
| VDVDEDATVGDALDALVGAHPALESR | 16 | E | $3.3882 \times E^{-9}$ |
| VDVDKDATVGDALDALVGAHPALESR | 4 | K | $2.2907 \times E^{-15}$ |
| VDVDQDATVGDALDALVGAHPALESR | 4 | Q | $6.9019 \times E^{-16}$ |
| VDVDVDATVGDALDALVGAHPALESR | 3 | V | $3.1913 \times E^{-13}$ |
| VDVDSDATVGDALDALVGAHPALESR | 3 | S | $7.2773 \times E^{-16}$ |
| VDVDNDATVGDALDALVGAHPALESR | 2 | N | $1.1245 \times E^{-11}$ |
| VDVDGDATVGDALDALVGAHPALESR | 2 | G | $2.3279 \times E^{-11}$ |
| VDVDHDATVGDALDALVGAHPALESR | 2 | H | $1.4688 \times E^{-10}$ |
| VDVDFDATVGDALDALVGAHPALESR | 2 | F | $1.5487 \times E^{-8}$ |
| VDVDMDATVGDALDALVGAHPALESR | 2 | M | $1.5739 \times E^{-8}$ |
| VDVDPDATVGDALDALVGAHPALESR | 1 | P | $3.5807 \times E^{-15}$ |
| VDVDADATVGDALDALVGAHPALESR | 1 | A | $1.4927 \times E^{-14}$ |

## Supplemental References


1. Allers, T., Ngo, H. P., Mevarech, M., and Lloyd, R. G. (2004) Development of additional selectable markers for the halophilic archaeon *Haloferax volcanii* based on the *leuB* and *trpA* genes. *Appl Environ Microbiol* **70**, 943-953
2. Zhou, G., Kowalczyk, D., Humbard, M., Rohatgi, S., and Maupin-Furlow, J. (2008) Proteasomal components required for cell growth and stress responses in the halophilic *Haloferax volcanii*. *J Bacteriol* **190**, 8096-8105


**Additional File 1**. PylS tree (Newick format)

```
(WP_013193804_1__Methanohalobium_evestigatum_Z-7303:0.2994823124,
(((WP_015323491_1__Methanomethylovorans_hollandica_DSM_15978:0.1625647465,
(WP_015053640_1__Methanolobus_psychrophilus_R15:0.0640917271,WP_023845214_1__Methanolobus_tindarius_DSM_2278:0
.1042520346)1:0.0712566252)1:0.0764482649,WP_013898320_1__Methanosalsum_zhilinae_DSM_4017:0.1728411498)0.996:0
.0469714590,
((WP_011500100_1__Methanococcoides_burtonii_DSM_6242:0.0790998328,WP_048204558_1__Methanococcoides_methylutens
_MM1:0.1152679723)1:0.1059839619,((((WP_048181983_1__Methanosarcina_sp_MTP4:0.0895180057,
(((((WP_011305865_1__Methanosarcina_barkeri_str_Wiesmoor:0.0377711779,
(WP_048123171_1__Methanosarcina_vacuolata_Z-761:0.0000009996,WP_048157962_1__Methanosarcina_sp_Kolksee:0.00460
41148)0.333:0.0000022652)1:0.0657074501,
(WP_048167695_1__Methanosarcina_thermophila_TM-1:0.0190598183,WP_054298905_1__Methanosarcina_flavescens_E03_2:
0.0379411794)0.865:0.0156311279)1:0.0446370957,WP_048178985_1__Methanosarcina_siciliae_C2J:0.0222126011)0.78:0
.0164889832,WP_011020213_1__Methanosarcina_acetivorans_C2A:0.0225855294)0.986:0.0299177983,
((WP_011033391_1__Methanosarcina_mazei_Go1:0.0000009996,WP_048050733_1__Methanosarcina_soligelidi_SMA-21:0.009
3249217)1:0.0210569060,WP_048142694_1__Methanosarcina_horonobensis_HB-1_JCM_15518:0.0172069267)0.983:0.0214676
410)1:0.0432954355)0.619:0.0056643849,
(WP_048169762_1__Methanosarcina_sp_2_H_A_1B_4:0.0044591725,WP_048160059_1__Methanosarcina_sp_2_H_T_1A_6:0.0046
342860)1:0.0180716002)1:0.0238395783,WP_048128743_1__Methanosarcina_lacustris_Z-7289:0.0140391815)0.509:0.0041
010863,WP_048130001_1__Methanosarcina_sp_WWM596:0.0050618330)1:0.1745675096)0.877:0.0391073908)0.996:0.0879056
189,(((WP_072560754_1__Methanohalophilus_halophilus_Z-7982:0.0041960241,
(WP_072358806_1__Methanohalophilus_portucalensis_FDF-1T:0.0087572135,
(OBZ34501_1__Methanohalophilus_sp_DAL1:0.0044520382,WP_096712291_1__Methanohalophilus_euhalobius_DSM_10369:0.0
043462336)1:0.0182384866)1:0.0183490479)0.928:0.0138255547,
(WP_013036758_1__Methanohalophilus_mahii_DSM_5219:0.0134497612,OBZ34613_1__Methanohalophilus_sp_DAL1:0.0228833
002)0.333:0.0000025403)1:0.1848098299,((((WP_066075773_1__methanogenic_archaeon_ISO4-H5:0.3017886419,
(WP_015505008_1__C_Methanomethylophilus_alvus_Mx1201:0.0475160191,WP_015492598_1__Thermoplasmatales_archaeon_B
RNA1:0.1984540654)1:0.2053661894)0.938:0.1210200282,WP_048111907_1__C_Methanoplasma_termitum_MpT1:0.2170805126
)1:0.3474366568,
((WP_042686913_1__Methermicoccus_shengliensis_DSM_18856:0.4280133234,WP_019178529_1__Methanomassiliicoccus_lum
inyensis_B10:0.3702713048)0.456:0.0442201124,
(WP_019176308_1__Methanomassiliicoccus_luminyensis_B10:0.4455402308,WP_020448777_1__C_Methanomassiliicoccus_in
testinalis_Issoi:0.3752143935)0.851:0.1043272773)0.861:0.0800718371)1:0.3690444254,
(WP_086636640_1__Methanonatronarchaeum_thermophilum_AMET1:0.4483155932,
(OKY79096_1__C_Methanohalarchaeum_thermophilum:0.5003916787,OKY77552_1__C_Methanohalarchaeum_thermophilum:0.47
62101644)0.999:0.2390852175)1:0.5595916843)1:0.8669441079)0.82:0.0982908753);
```
